# Investigations into the structure–activity relationship in gemini QACs based on biphenyl and oxydiphenyl linker†

**DOI:** 10.1039/d0ra08900a

**Published:** 2021-01-18

**Authors:** Anatoly N. Vereshchagin, Nikita A. Frolov, Valeria Yu Konyuhova, Ekaterina A. Kapelistaya, Karl A. Hansford, Mikhail P. Egorov

**Affiliations:** N. D. Zelinsky Institute of Organic Chemistry, Russian Academy of Sciences 47 Leninsky Procpekt 119991 Moscow Russia vereshchagin@ioc.ac.ru; Institute for Molecular Bioscience, The University of Queensland Brisbane Queensland 4072 Australia

## Abstract

Eighteen novel gemini quaternary ammonium compounds were synthesized to examine the effect of linker nature, aliphatic chain length and their relative position on antibacterial and antifungal activity. The synthesized compounds showed strong bacteriostatic activity against a panel of both Gram-positive and Gram-negative bacteria, including methicillin-resistant *Staphylococcus aureus* (MRSA) and two fungi. Some of these compounds exhibited a wider and more potent antimicrobial spectrum than commonly-used antiseptics, such as benzalkonium chloride (BAC), cetylpyridinium chloride (CPC), chlorhexidine digluconate (CHG) and octenidine dihydrochloride (OCT).

## Abstract 2

The synthesis and biological evaluations of new gemini QACs possessing high antibacterial and antifungal activity were performed.

## Graphical abstract

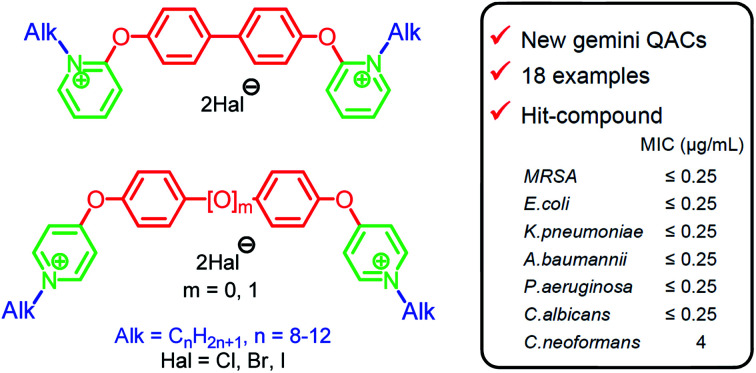

## Introduction

Quaternary ammonium compounds (QACs) are commonly used in medicine and industry. These cationic surfactants are applied as preservatives, antiseptics and disinfectants.^1^ The first observations of antimicrobial activity among QAC were published in 1916,^2^ however full potential of this class of agents wasn't realized until 1930s, when benzalkonium chloride (BAC) and cetyl pyridinium chloride (CPC) were discovered.^3^

**Fig. 1:**
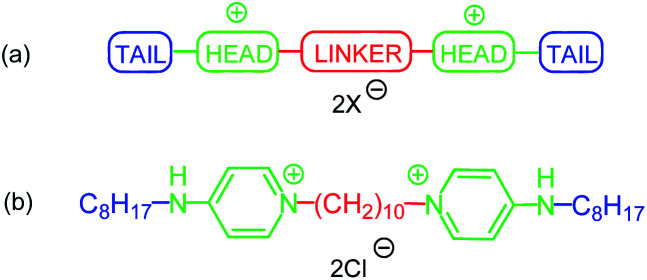
(a) General structure of cationic gemini surfactants, (b) structure of octenidine.

Symmetrical bis-quaternary ammonium compounds (bis-QACs), or gemini QACs, are built of two monomeric QAC molecules linked by a spacer (Fig. 1a).^4,5^ Bis-QACs activity against microorganisms is generally stronger comparing to corresponding monomeric compounds and depends on structure of the gemini molecule.^4*c*,4*g*,5*d*^ Within a range of bis-QACs, cationic gemini bispyridinium salts play important role being broadly used as biocides. They possess strong antimicrobial effect on Gram-positive and Gram-negative bacteria, fungi and some viruses, even in very low concentrations.^6^ Usually they consist of two pyridine-containing heads, that are substituted with aliphatic, alkenilic or alkynilic chain (tail) in *meta-* or *para-*positions to a spacer. One of the most effective antiseptics – octenidine dihydrochloride – can be an example (Fig. 1b).

**Fig. 2:**
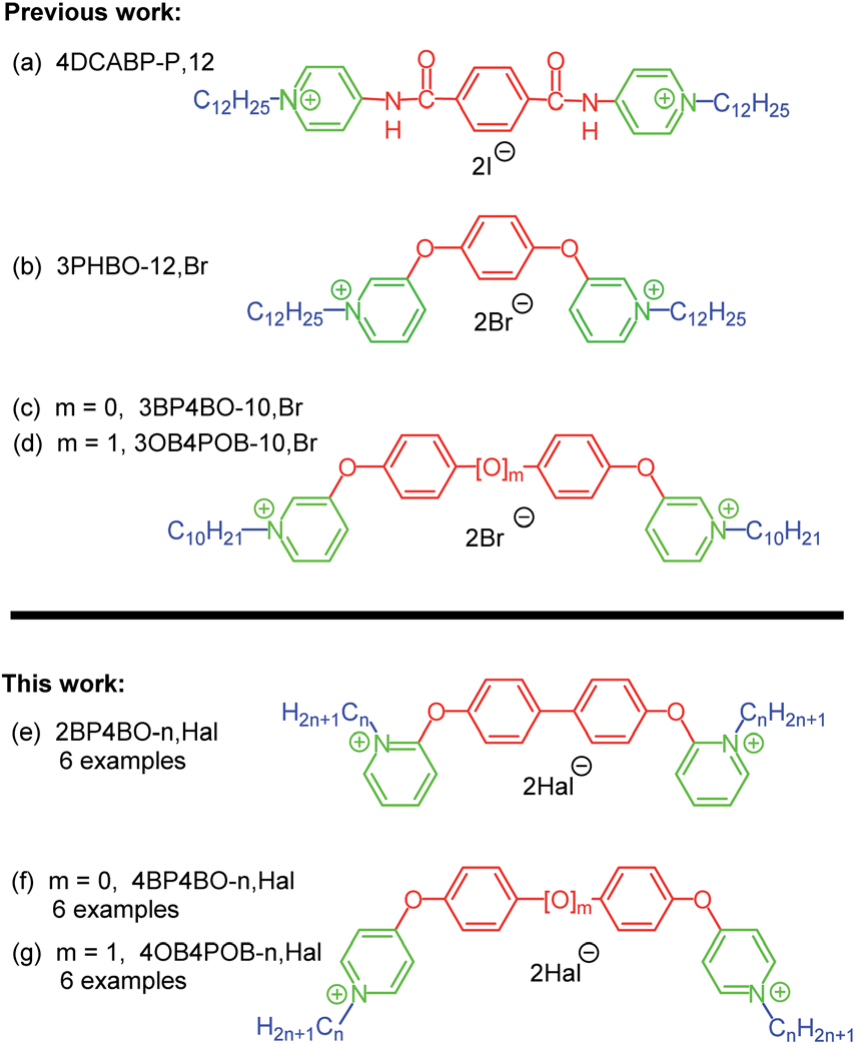
Structure of cationic gemini surfactants with benzene containing linker.

Structure–activity relation studies appear to be one of modern interdisciplinary approaches in organic chemistry.^7^ Spacers' nature is known to have significant impact on certain effects of bis-QACs’ including biocidal.^4*h*^ Over the past decades plenty of spacer variations in bispyridinium salts have been obtained and showed antibacterial, antifungal and antimalarial activity.^6,8^ Amongst others, bis-QACs with benzene ring as a spacer were synthesized, for instance 4DCABP-P,12 (Fig. 2a)^6*b*^ or 3PHBO-12,Br (Fig. 2b).^8*e*^ These compounds have same activity with octenidine (MIC and MBC), but better in terms of cytotoxicity (normal human epidermal keratinocytes).^9^

Recently we have got structural analogues of 3PHBO-12, that contain either biphenyl-4,4′-diol^10*a*^ (3BP4BO-*n*,Hal; Fig. 2c), or 4,4′-oxydiphenol^10*b*^ (3OB4POB-*n*,Hal; Fig. 2d) spacers. MIC values of the hit-compounds against Gram-negative *Escherichia coli* (ATCC 25922), *Klebsiella pneumonia* (ATCC 700603), *Acinetobacter baumannii* (ATCC 19606) and *Pseudomonas aeruginosa* (ATCC 27853) were lower than that of 3PHBO-12, BAC and CHG.^10^ It is important that linkers in bis-QACs 3BP4BO-*n*,Hal and 3OB4POB-*n*,Hal are in *meta-*positions to aliphatic tails. The new amphiphiles were obtained *via* reaction of dibromides (4,4′-dibromobiphenyl or 1,1′-oxybis(4-bromobenzene)) with 3-hydroxypyridine under basic conditions in presence of copper powder (Ullman-type reaction), followed by *N*-alkylation with alkyl halides of the preformed platforms 3BP4BO and 3OB4POB. Within this study we sought to examine the influence of different positions of the pyridinium head connection with the same aromatic linker on biological activity (Fig. 2e–g).

## Results and discussion

### Chemistry

#### Compound design

**Scheme 1:**
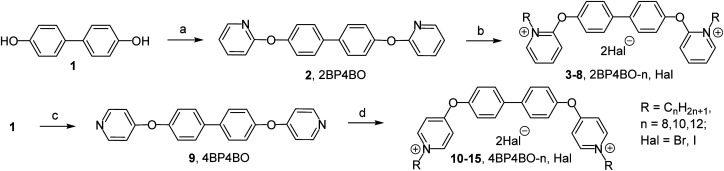
Reagents and conditions: (a) 2-bromopyridine, CuI(i), K_3_PO_4_, picolinic acid, DMSO, 90 °C, argon, 24 h, 77%; (b) RHal, acetonitrile, 82 °C, 7 days, 36–58%; (c) 4-chloropyridine hydrochloride, CuI(i), K_3_PO_4_, picolinic acid, DMSO, 140 °C, argon, 72 h, 85%; (d) RHal, acetonitrile, 82 °C, 72 h, 75–82%.

**Scheme 2:**
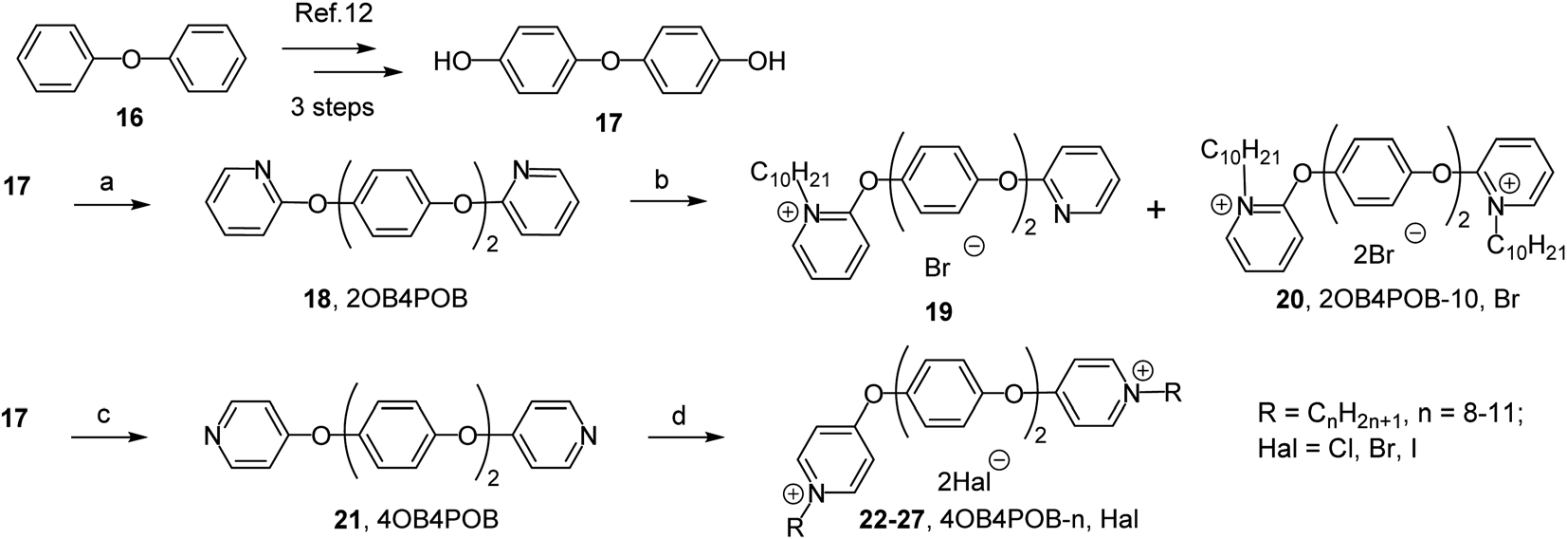
Reagents and conditions: (a) 2-bromopyridine, CuI(i), K_3_PO_4_, picolinic acid, DMSO, 90 °C, argon, 24 h, 77%; (b) RHal, acetonitrile, 82 °C, 7 days, 26–58%; (c) 4-chloropyridine hydrochloride, CuI(i), K_3_PO_4_, picolinic acid, DMSO, 140 °C, argon, 72 h, 85%; (d) RHal, acetonitrile, 82 °C, 72 h, 65–82%.

The aim was first to get *ortho*- and *para*-substituted derivatives with biphenyl-4,4′-diol (Scheme 1) and 4,4′-oxydiphenol (Scheme 2) spacers. Initially, the challenge was to synthesize in accordance with our previous work^10^ the platforms head-spacer-head: 2,2′-[biphenyl-4,4′-diylbis(oxy)]dipyridine (2BP4BO) 2 and 4,4′-[biphenyl-4,4′-diylbis(oxy)]dipyridine (4BP4BO) 9 from 2-hydroxy- and 4-hydroxypyridine respectively. But the approach did not lead to a desired result. We observed the reaction mass tarring. Afterwards an alternative method of head–spacer–head platforms synthesis was applied. We have got 2BP4BO 2 and 4BP4BO 9 from affordable biphenyl-4,4′-diol 1 and 2- and 4-halogenpyridines (Scheme 1) through Ullmann-type reaction.^11^ Bromopyridines are more reactive than chloropyridines. So, the conversion of diol 1 was 100% after 24 hours of heating in DMSO in inert atmosphere, and 2BP4BO 2 was obtained with 77% yield. When using 2-chloropyridine, full conversion of 1 under given conditions is achieved only after 72 hours and the yield of 2 was 62%. 4-Chloropyridine hydrochloride was used to obtain 4BP4BO 9. To accelerate the reaction its temperature was increased to 140 °C. 4BP4BO 9 was obtained with 85% yield after heating for 72 hours. Similarly, head-spacer-head platforms synthesis 2,2′-[oxybis(4,1-phenyleneoxy)]dipyridine (2OB4POB) 18 and 4,4′-[oxybis(4,1-phenyleneoxy)]dipyridine (4OB4POB) 21 containing a 4,4′-oxydiphenol spacer, have been carried out (Scheme 2). We performed the synthesis of commercially unavailable diol 17*via* three steps from biphenyl ether 16 according to known method: Friedel–Crafts acylation of biphenyl ether, oxidation of the diacetyl derivative by mCPBA, alkaline hydrolysis of the resulting diester.^12^ At the second step of novel gemini amphiphiles synthesis the platforms quaternization with alkyl halides was performed. It was found that the nitrogen position in the pyridinium head relative to the linker is crucial for *N*-alkylation.

Indeed the alkylation process of *ortho*-platform 2 is very slow even in the presence of alkyl halides excess. 2,2′-[Biphenyl-4,4′-diylbis(oxy)]bis(1-alkylpyridinium) dihalides 2BP4BO-*n*,Hal 3–8 were obtained with 36–58% yields. For 18 platform alkylation is nonselective. With continuous refluxing of 18 in acetonitrile in excess of alkylbromide a mixture of mono-QAC 19 and bis-QAC 20 was obtained at ratio 1 : 2 (by NMR). The poor reactivity of ‘*ortho*’ nitrogen is known. For example, various data shows that even with excess of alkyl halide orthoquat alkylation leads to either low yield mono-QAC formation^13*a*^ or does not occur at all.^13*b*^ Alkylation of *para*-platforms 9 and 21 proceeded noticeably better. After 72 hours of refluxing in acetonitrile, complete conversion of the starting compounds and the formation of bis-QACs 10–15 in 75–82% yields and 22–27 in 65–82% yields was observed.

### Biological evaluation

Next, we determined the influence of linkers, aliphatic tail length, their relative position and counterion's nature on antibacterial and antifungal activity. *In vitro* activity against a panel of five bacteria, including both Gram-positive [methicillin-resistant *Staphylococcus aureus* (MRSA), strain ATCC 43300] and Gram-negative (*Escherichia coli*, ATCC 25922; *Klebsiella pneumoniae*, ATCC 700603; *Acinetobacter baumannii*, ATCC 19606*; Pseudomonas aeruginosa*, ATCC 27853) strains, two fungi (*Candida albicans*, ATCC 90028; *Cryptococcus neoformans* var. *Grubii*, ATCC 208821), was evaluated for all synthesized *ortho*- and *para*-bis-QACs (Table 1). Cytotoxicity on human embryonic kidney cells (HEK-293, ATCC CRL-1573, CC_50_) and haemolytic activity on human red blood cells (RBC, HC_50_) were also tested (Table 1). Previously synthesized *meta-*bis-QACs containing biphenyl^10*a*^ (3BP4BO-10,Br) and oxydiphenyl^10*b*^ (3OB4POB-10,Br) linker and commonly-used antiseptics such as benzalkonium chloride (BAC), cetylpyridinium chloride (CPC), chlorhexidine digluconate (CHG) and octenidine dihydrochloride (OCT) were tested as reference compounds. Procedure and materials used in microbiological assays were performed by CO-ADD (the Community for Antimicrobial Drug Discovery).^14^

**Table 1:** MIC and cytotoxicity values (μg mL^−1^) for obtained bis-QACs

Compound	MIC^a^							Cytotoxicity^b^	
	Bacteria					Fungi		HEK-293 (CC_50_)	RBC (HC_50_)
	MRSA	*E. coli*	*K. pneumoniae*	*A. baumannii*	*P. aeruginosa*	*C. albicans*	*C. neoformans*		
3, 2BP4BO-8,Br	≤0.25	2	8	16	16	2	1	14.6	15.2
4, 2BP4BO-8,I	≤0.25	4	16	32	16	4	8	19.3	18.4
5, 2BP4BO-10,Br	≤0.25	16	32	16	16	≤0.25	≤0.25	12.4	2.7
6, 2BP4BO-10,I	0.5	32	32	32	32	0.5	≤0.25	7.8	5.7
7, 2BP4BO-12,Br	2	32	>32	>32	32	1	≤0.25	0.9	2.9
8, 2BP4BO-12,I	1	32	>32	>32	32	0.5	0.5	3.1	2.7
10, 4BP4BO-8,Br	≤0.25	≤0.25	16	16	16	1	1	11.6	27.0
11, 4BP4BO-8,I	≤0.25	≤0.25	16	16	16	1	1	>32	>32
12, 4BP4BO-10,Br	≤0.25	8	16	4	8	≤0.25	≤0.25	4.8	7.2
13, 4BP4BO-10,I	≤0.25	8	32	16	16	≤0.25	1	14.4	>32
14, 4BP4BO-12,Br	0.5	>32	>32	>32	>32	0.5	≤0.25	13.6	>32
15, 4BP4BO-12,I	1	32	>32	>32	32	2	0.5	16.6	>32
19 + 20, (2OB4POB-10,Br)	2	>32	>32	>32	>32	2	4	24.6	>32
22, 4OB4POB-8,Br	≤0.25	≤0.25	8	16	32	≤0.25	2	2.8	17.9
23, 4OB4POB-9,Br	≤0.25	≤0.25	≤0.25	≤0.25	≤0.25	≤0.25	4	1.2	11.9
24, 4OB4POB-10,Cl	≤0.25	4	32	16	16	≤0.25	≤0.25	8.5	26.0
25, 4OB4POB-10,Br	≤0.25	≤0.25	4	≤0.25	4	≤0.25	16	0.4	≤0.25
26, 4OB4POB-10,I	≤0.25	≤0.25	32	≤0.25	4	≤0.25	32	3.2	0.6
27, 4OB4POB-11,Br	≤0.25	4	>32	16	16	≤0.25	32	0.5	≤0.25
BAC	0.5	16	>32	32	>32	0.5	1	2.8	3.4
CPC	≤0.25	16	>32	>32	32	≤0.25	≤0.25	1.5	2.7
CHG	≤0.25	1	32	8	8	32	>32	>32	>32
OCT	≤0.25	≤0.25	≤0.25	≤0.25	0.25	≤0.25	8	1.6	4.2
3BP4BO-10,Br	≤0.25	1	8	2	4	≤0.25	≤0.25	3.1	16.9
3OB4POB-10,Br	≤0.25	1	4	2	4	≤0.25	≤0.25	3.2	4.3

a MRSA, methicillin-resistant *Staphylococcus aureus* (ATCC 43300); *E. coli*, *Escherichia coli* (ATCC 25922); *K. pneumonia*, *Klebsiella pneumonia* (ATCC 700603); *A. baumannii*, *Acinetobacter baumannii* (ATCC 19606); *P. aeruginosa*, *Pseudomonas aeruginosa* (ATCC 27853), *C. albicans*, *Candida albicans* (ATCC 90028); *C. neoformans*, *Cryptococcus neoformans* var. *Grubii* (ATCC 208821).

b HEK-293, human embryonic kidney cells (ATCC CRL-1573, CC_50_); RBC, human red blood cells (HC_50_). BAC – benzalkonium chloride, CPC – cetylpyridinium chloride, CHG – chlorhexidine digluconate, OCT – octenidine dihydrochloride.

A number of trends can be drawn based on the results of microbiological studies given in Table 1. The vast majority of QACs tested show high activity against MRSA and fungi (especially against *C. albicans*). Gram-negative bacteria are more resistant to new bis-QACs. The position of nitrogen atoms relative to the linker, as well as the length of the alkyl chain, are the determining factors of activity. *Ortho*-salts (3–8 and a mixture of 19 + 20) are significantly inferior in efficiency to both *para-* and *meta-*derivatives and in some cases are inactive. Bis-QACs with alkyl substituents in the C8–C10 range (compounds 10–13, 22–26) have the broadest spectrum of activity. These gemini amphiphiles are superior to BAC, CPC, and CHG in their activity against Gram-negative bacteria. Among the salts tested, a hit-compound is clearly visible. That is 4,4′-[oxybis(4,1-phenyleneoxy)]bis(1-nonylpyridinium)dibromide 23 containing an oxydiphenyl linker and a C_9_H_19_ alkyl substituent. This compound is not inferior to OCT in bacteriostatic effect on the entire spectrum of the studied strains. As well as OCT, it is toxic against HEK-293 and outperforms OCT against RBC. The HC_50_ of 23 is four times higher than the HC_50_ of octenidine. There is no clear dependence of the influence of the counterion on activity (see amphiphiles 24–26). However, this dependence is observed in the series of *ortho-*, *meta*-, *para*-derivatives. In general, antibacterial activity decreases in the order *meta*- > *para*- > *ortho*- for bis-QACs containing a biphenyl linker and in the *para*- > *meta*- > *ortho*- order for bis-QACs containing an oxybiphenyl linker.

## Conclusions

Thus, a methodology for the simple synthesis of *ortho*, *meta*, and *para* bis-QACs containing biphenyl and oxybiphenyl linkers from available starting material was developed. The antibacterial and antifungal activity of the obtained compounds were studied on five pathogenic bacteria and two yeasts. The effect of the location of the linker relative to the pyridinium head, as well as the length of the alkyl substituent, on microbiological activity were established. In general, for bis-QACs containing a biphenyl linker, antibacterial activity decreases in the order *meta*- > *para*- > *ortho*-. For bis-QACs containing an oxybiphenyl linker, the antibacterial activity decreases in the *para*- > *meta*- > *ortho*- order. The gemini amphiphile 4OB4POB-9,Br, containing an oxydiphenyl linker and a C_9_H_19_ alkyl substituent were identified as a hit-compound. This bis-QAC is significantly superior to the widely used BAC, CPC, and CHG in bacteriostatic effect, comparable in activity to OCT, but superior to it in cytotoxicity to human red blood cells. These results indicate that 4OB4POB-9,Br can be successfully used as a new antiseptic.

## Experimental

### Chemistry

#### General experimental

Analytical thin layer chromatography (TLC) was performed with EM Science silica gel 60 F254 aluminium plates. Visualisation was carried out using a UV lamp (254 nm). Organic solutions were concentrated by rotary evaporation at 70–80 °C.

#### Materials

Unless otherwise noted, all purchased materials were used without purification. All standard solvents were purchased from Acros Organics.

#### Instrumentation

^1^H and ^13^C NMR spectra were recorded on a Bruker AM300 (300 MHz for ^1^H, 75 MHz for ^13^C) and Bruker DRX500 (500 MHz for ^1^H, 125 MHz for ^13^C) spectrometers at ambient temperature in DMSO-*d*_6_ and CDCl_3_. Chemical shifts are reported relative to residual solvent peaks and coupling constants (*J*) are given in hertz. Bis-QACs purities were confirmed by HPLC on a Stayer 0892 series HPLC system with Luna® 5 μm C18 100 Å, LC column 250 × 4.6 mm. Mobile phase: 85 : 15 MeCN/H_2_O (0.25 M NaClO_4_, 0.1% H_3_PO_4_). All melting points were determined on a Gallenkamp melting point apparatus in open capillaries and are uncorrected. Mass spectra were recorded on a Finnigan MAT INCOS 50 mass-spectrometer. IR spectra were recorded with a Bruker ALPHA-T FT-IR spectrometer in KBr pellets.

### Compound synthesis

#### Preparation of 2

##### 2,2′-[Biphenyl-4,4′-diylbis(oxy)]dipyridine (2BP4BO)

The mixture of 4,4′-dihydroxy-1,1′-biphenyl (1) (1.86 g, 10 mmol), 2-bromopyridine (3.16 g, 20 mmol), potassium phosphate (8.48 g, 40 mmol), copper(i) iodide (1.90 g, 10 mmol) and picolinic acid (0.25 g, 2 mmol) in dry DMSO (50 mL) was heated at 90 °C for 24 hours in argon atmosphere. Solvent was removed under reduced pressure, ethyl acetate (50 mL) was added to crude residue, and the mixture was heated to reflux for 1 hour. Then mixture was filtered off. The organic filtrate was concentrated under reduced pressure and the residue was purified by recrystallization from heptane to afford 2,2′-[biphenyl-4,4′-diylbis(oxy)]dipyridine (2) (2.62 g, 77% yield).

C_22_H_16_N_2_O_2_; *M*_w_ 340.4; white solid; mp 115–120 °C; ^1^H NMR (500 MHz, CDCl_3_): *δ* 6.99 (d, *J* = 8.2 Hz, 2H, 2CH_py_), 7.04 (t, *J* = 6.4 Hz, 2H, 2CH_py_), 7.23 (d, *J* = 8.5 Hz, 4H, CH_Ar_), 7.63 (d, *J* = 8.5 Hz, 4H, CH_Ar_), 7.73 (t, *J* = 8.2 Hz, 2H, 2CH_py_), 8.26 (d, *J* = 6.4 Hz, 2H, 2CH_py_) ppm; ^13^C NMR (125 MHz, CDCl_3_): *δ* 111.7 (2C), 119.2 (2C), 121.7 (4C), 127.9 (4C), 135.9 (2C), 140.3 (2C), 147.5 (2C), 153.4 (2C), 163.0 (2C) ppm.

#### Preparations of 3–8

Alkyl halide (10 mmol) was added to a solution of 2,2′-[biphenyl-4,4′-diylbis(oxy)]dipyridine (2) (0.34 g, 1 mmol) in acetonitrile (3 mL). The mixture was heated under reflux for 7 days, then allowed to cool to room temperature and filtered off. The solid was washed with 10 mL of cold acetone and dried to give a bis-QAC. The yields of bis-QACs 3–8 were 36–58% depend on alkyl halide.

##### 2,2′-[Biphenyl-4,4′-diylbis(oxy)]bis(1-octylpyridinium)dibromide (3, 2BP4BO-8,Br)

C_38_H_50_Br_2_N_2_O_2_; *M*_w_ 726.6; white solid (0.28 g, 38% yield); mp 123–125 °C; ^1^H NMR (300 MHz, DMSO-*d*_6_): *δ* 0.84 (t, *J* = 6.4 Hz, 6H, 2CH_3_), 1.18–1.51 (m, 20H, 10CH_2_), 1.91–2.07 (m, 4H, 2CH_2_), 4.68 (t, *J* = 6.8 Hz, 4H, 2CH_2_N^+^), 7.31 (d, *J* = 8.7 Hz, 2H, 2CH_py_), 7.65 (d, *J* = 8.4 Hz, 4H, CH_Ar_), 7.78 (t, *J* = 6.4 Hz, 2H, 2CH_py_), 8.01 (d, *J* = 8.4 Hz, 4H, CH_Ar_), 8.50 (t, *J* = 8.7 Hz, 2H, 2CH_py_), 9.04 (d, *J* = 6.4 Hz, 2H, 2CH_py_) ppm; ^13^C NMR (75 MHz, DMSO-*d*_6_): *δ* 13.9 (2C), 21.9 (2C), 25.6 (2C), 28.3 (2C), 28.4 (2C), 28.6 (2C), 31.1 (2C), 54.7 (2C), 113.5 (2C), 120.5 (2C), 121.6 (4C), 129.3 (4C), 138.1 (2C), 143.6 (2C), 148.4 (2C), 151.3 (2C), 158.8 (2C) ppm; *m*/*z* (%): 340 (100), 312 (14), 284 (7), 262 (6), 234 (4), 78 (8), 51 (3), 44 (2), 28 (4), 18 (6); *ν*_max_, (KBr): 3421, 2926, 2856, 1633, 1581, 1491, 1463, 1306, 1159, 781 cm^−1^.

##### 2,2′-[Biphenyl-4,4′-diylbis(oxy)]bis(1-octylpyridinium)diiodide (4, 2BP4BO-8,I)

C_38_H_50_I_2_N_2_O_2_; *M*_w_ 820.6; yellow solid (0.39 g, 48% yield); mp 194–198 °C; ^1^H NMR (300 MHz, DMSO-*d*_6_): *δ* 0.85 (t, *J* = 6.4 Hz, 6H, 2CH_3_), 1.18–1.47 (m, 20H, 10CH_2_), 1.91–2.05 (m, 4H, 2CH_2_), 4.64 (t, *J* = 6.8 Hz, 4H, 2CH_2_N^+^), 7.31 (d, *J* = 8.7 Hz, 2H, 2CH_py_), 7.63 (d, *J* = 8.4 Hz, 4H, CH_Ar_), 7.77 (t, *J* = 6.4 Hz, 2H, 2CH_py_), 8.00 (d, *J* = 8.4 Hz, 4H, CH_Ar_), 8.46 (t, *J* = 8.7 Hz, 2H, 2CH_py_), 8.92 (d, *J* = 6.4 Hz, 2H, 2CH_py_) ppm; ^13^C NMR (75 MHz, DMSO-*d*_6_): *δ* 13.9 (2C), 21.9 (2C), 25.6 (2C), 28.3 (2C), 28.4 (2C), 28.6 (2C), 31.1 (2C), 54.7 (2C), 113.5 (2C), 120.5 (2C), 121.6 (4C), 129.3 (4C), 138.1 (2C), 143.5 (2C), 148.4 (2C), 151.3 (2C), 158.8 (2C) ppm; *m*/*z* (%): 340 (100), 312 (16), 284 (9), 262 (8), 240 (10), 206 (8), 71 (41), 57 (26), 43 (24), 29 (10); *ν*_max_, (KBr): 3442, 2926, 2855, 1633, 1581, 1490, 1308, 1194, 1156, 773 cm^−1^.

##### 2,2′-[Biphenyl-4,4′-diylbis(oxy)]bis(1-decylpyridinium)dibromide (5, 2BP4BO-10,Br)

C_42_H_58_Br_2_N_2_O_2_; *M*_w_ 782.8; white solid (0.33 g, 42% yield); mp 174–175 °C; ^1^H NMR (300 MHz, DMSO-*d*_6_): *δ* 0.83 (t, *J* = 6.4 Hz, 6H, 2CH_3_), 1.18–1.49 (m, 28H, 14CH_2_), 1.91–2.05 (m, 4H, 2CH_2_), 4.64 (t, *J* = 6.8 Hz, 4H, 2CH_2_N^+^), 7.31 (d, *J* = 8.7 Hz, 2H, 2CH_py_), 7.63 (d, *J* = 8.4 Hz, 4H, CH_Ar_), 7.77 (t, *J* = 6.4 Hz, 2H, 2CH_py_), 8.00 (d, *J* = 8.4 Hz, 4H, CH_Ar_), 8.47 (t, *J* = 8.7 Hz, 2H, 2CH_py_), 8.95 (d, *J* = 6.4 Hz, 2H, 2CH_py_) ppm; ^13^C NMR (75 MHz, DMSO-*d*_6_): *δ* 13.9 (2C), 21.9 (2C), 25.6 (2C), 28.3 (2C), 28.6 (2C), 28.7 (4C), 28.8 (2C), 31.2 (2C), 54.7 (2C), 113.5 (2C), 120.5 (2C), 121.6 (4C), 129.3 (4C), 138.1 (2C), 143.6 (2C), 148.4 (2C), 151.3 (2C), 158.8 (2C) ppm; *m*/*z* (%): 340 (100), 312 (29), 284 (15), 262 (9), 234 (9), 137 (14), 78 (24), 71 (3), 55 (12), 41 (11); *ν*_max_, (KBr): 3433, 2923, 2854, 1632, 1579, 1491, 1463, 1302, 1155, 782 cm^−1^.

##### 2,2′-[Biphenyl-4,4′-diylbis(oxy)]bis(1-decylpyridinium)diiodide (6, 2BP4BO-10,I)

C_42_H_58_I_2_N_2_O_2_; *M*_w_ 876.8; yellow solid (0.35 g, 40% yield); mp 185–188 °C; ^1^H NMR (300 MHz, DMSO-*d*_6_): *δ* 0.83 (t, *J* = 6.4 Hz, 6H, 2CH_3_), 1.18–1.49 (m, 28H, 14CH_2_), 1.91–2.05 (m, 4H, 2CH_2_), 4.64 (t, *J* = 6.8 Hz, 4H, 2CH_2_N^+^), 7.31 (d, *J* = 8.7 Hz, 2H, 2CH_py_), 7.63 (d, *J* = 8.4 Hz, 4H, CH_Ar_), 7.76 (t, *J* = 6.4 Hz, 2H, 2CH_py_), 8.00 (d, *J* = 8.4 Hz, 4H, CH_Ar_), 8.46 (t, *J* = 8.7 Hz, 2H, 2CH_py_), 8.92 (d, *J* = 6.4 Hz, 2H, 2CH_py_) ppm; ^13^C NMR (75 MHz, DMSO-*d*_6_): *δ* 13.8 (2C), 21.9 (2C), 25.6 (2C), 28.3 (2C), 28.6 (C), 28.7 (4C), 28.8 (2C), 31.2 (2C), 54.7 (2C), 113.5 (2C), 120.5 (2C), 121.6 (4C), 129.3 (4C), 138.0 (2C), 143.5 (2C), 148.3 (2C), 151.2 (2C), 158.8 (2C) ppm; *m*/*z* (%): 340 (28), 268 (25), 155 (22), 141 (22), 127 (14), 85 (89), 71 (70), 57 (100), 43 (93), 29 (39); *ν*_max_, (KBr): 3427, 2925, 2853, 1634, 1581, 1490, 1462, 1307, 1156, 772 cm^−1^.

##### 2,2′-[Biphenyl-4,4′-diylbis(oxy)]bis(1-dodecylpyridinium)dibromide (7, 2BP4BO-12,Br)

C_46_H_66_Br_2_N_2_O_2_; *M*_w_ 838.9; white solid (0.3 g, 36% yield); mp 190–192 °C; ^1^H NMR (300 MHz, DMSO-*d*_6_): *δ* 0.83 (t, *J* = 6.4 Hz, 6H, 2CH_3_), 1.18–1.49 (m, 36H, 18CH_2_), 1.91–2.02 (m, 4H, 2CH_2_), 4.64 (t, *J* = 6.8 Hz, 4H, 2CH_2_N^+^), 7.30 (d, *J* = 8.7 Hz, 2H, 2CH_py_), 7.62 (d, *J* = 8.4 Hz, 4H, CH_Ar_), 7.76 (t, *J* = 6.4 Hz, 2H, 2CH_py_), 8.01 (d, *J* = 8.4 Hz, 4H, CH_Ar_), 8.47 (t, *J* = 8.7 Hz, 2H, 2CH_py_), 8.94 (d, *J* = 6.4 Hz, 2H, 2CH_py_) ppm; ^13^C NMR (75 MHz, DMSO-*d*_6_): *δ* 13.8 (2C), 22.0 (2C), 25.6 (2C), 28.3 (2C), 28.6 (C), 28.7 (2C), 28.8 (4C), 28.9 (4C), 31.2 (2C), 54.7 (2C), 113.5 (2C), 120.5 (2C), 121.6 (4C), 129.3 (4C), 138.0 (2C), 143.5 (2C), 148.3 (2C), 151.4 (2C), 158.7 (2C) ppm; *m*/*z* (%): 340 (74), 311 (9), 284 (6), 263 (3), 149 (8), 137 (56), 99 (3), 85 (4), 57 (10), 43 (100); *ν*_max_, (KBr): 3433, 2922, 2853, 1632, 1579, 1490, 1464, 1298, 1154, 782 cm^−1^.

##### 2,2′-[Biphenyl-4,4′-diylbis(oxy)]bis(1-dodecylpyridinium)diiodide (8, 2BP4BO-12,I)

C_46_H_66_I_2_N_2_O_2_; *M*_w_ 932.9; yellow solid (0.54 g, 58% yield); mp 233–234 °C; ^1^H NMR (300 MHz, DMSO-*d*_6_): *δ* 0.83 (t, *J* = 6.4 Hz, 6H, 2CH_3_), 1.18–1.49 (m, 36H, 18CH_2_), 1.91–2.02 (m, 4H, 2CH_2_), 4.63 (t, *J* = 6.8 Hz, 4H, 2CH_2_N^+^), 7.30 (d, *J* = 8.7 Hz, 2H, 2CH_py_), 7.62 (d, *J* = 8.4 Hz, 4H, CH_Ar_), 7.76 (t, *J* = 6.4 Hz, 2H, 2CH_py_), 8.00 (d, *J* = 8.4 Hz, 4H, CH_Ar_), 8.46 (t, *J* = 8.7 Hz, 2H, 2CH_py_), 8.92 (d, *J* = 6.4 Hz, 2H, 2CH_py_) ppm; ^13^C NMR (75 MHz, DMSO-*d*_6_): *δ* 13.8 (2C), 21.9 (2C), 25.6 (2C), 28.3 (2C), 28.6 (C), 28.7 (4C), 28.8 (4C), 28.9 (2C), 31.2 (2C), 54.5 (2C), 113.5 (2C), 120.5 (2C), 121.6 (4C), 129.3 (4C), 138.0 (2C), 143.5 (2C), 148.3 (2C), 151.2 (2C), 158.8 (2C) ppm; *m*/*z* (%): 340 (100), 312 (16), 284 (14), 169 (6), 155 (6), 127 (7), 85 (23), 71 (10), 57 (14), 43 (11); *ν*_max_, (KBr): 3444, 2924, 2853, 1634, 1581, 1491, 1462, 1306, 1157, 773 cm^−1^.

#### Preparation of 9

##### 4,4′-[Biphenyl-4,4′-diylbis(oxy)]dipyridine (4BP4BO)

The mixture of 4,4′-dihydroxy-1,1′-biphenyl (1.86 g, 10 mmol), 4-chloropyridine hydrochloride (3.00 g, 20 mmol), potassium phosphate (16.96 g, 80 mmol), copper(i) iodide (1.90 g, 10 mmol) and picolinic acid (0.25 g, 2 mmol) in dry DMSO (50 mL) was heated to 140 °C for 72 hours in argon atmosphere. Solvent was removed under reduced pressure, ethyl acetate (50 mL) was added to crude residue, and the mixture was heated to reflux for 1 hour. Then mixture was filtered. The organic filtrate was concentrated under reduced pressure and the residue was purified by recrystallization from heptane to afford 4,4′-[biphenyl-4,4′-diylbis(oxy)]dipyridine (9) (2.89 g, 85% yield).

C_22_H_16_N_2_O_2_; *M*_w_ 340.4; white solid; mp 138–142 °C; ^1^H NMR (300 MHz, CDCl_3_): *δ* 6.92 (d, *J* = 6.9 Hz, 4H, 4CH_py_), 7.20 (d, *J* = 8.4 Hz, 4H, 4CH_Ar_), 7.65 (d, *J* = 8.4 Hz, 4H, 4CH_Ar_), 8.52 (d, *J* = 6.9 Hz, 4H, 4CH_py_) ppm; ^13^C NMR (75 MHz, CDCl_3_): *δ* 121.0 (4C), 128.6 (4C), 136.4 (4C), 138.3 (2C), 151.7 (2C), 153.2 (4C), 163.7 (2C) ppm.

#### Preparations of 10–15

Alkyl halide (2.2 mmol) was added to a solution of 4,4′-[biphenyl-4,4′-diylbis(oxy)]dipyridine (9) (0.34 g, 1 mmol) in acetonitrile (3 mL). The mixture was heated under reflux for 72 h, then allowed to cool to room temperature and filtered off. The solid was washed with 10 mL of cold acetone and dried to give a bis-QAC. The yields of bis-QACs 10–15 were 36–58% depend on alkyl halide.

##### 4,4′-[Biphenyl-4,4′-diylbis(oxy)]bis(1-octylpyridinium)dibromide (10, 4BP4BO-8,Br)

C_38_H_50_Br_2_N_2_O_2_; *M*_w_ 726.6; white solid (0.55 g, 75% yield); mp 245–247 °C; ^1^H NMR (300 MHz, DMSO-*d*_6_): *δ* 0.85 (t, *J* = 6.6 Hz, 6H, 2CH_3_), 1.17–1.42 (m, 20H, 10CH_2_), 1.81–1.93 (m, 4H, 2CH_2_), 4.52 (t, *J* = 7.3 Hz, 4H, 2CH_2_N^+^), 7.49 (d, *J* = 8.4 Hz, 4H, 4CH_Ar_), 7.62 (d, *J* = 6.9 Hz, 4H, 4CH_py_), 7.95 (d, *J* = 8.4 Hz, 4H, 4CH_Ar_), 9.01 (d, *J* = 6.9 Hz, 4H, 4CH_py_) ppm; ^13^C NMR (75 MHz, DMSO-*d*_6_): *δ* 13.9 (2C), 22.1 (2C), 25.4 (2C), 28.4 (2C), 28.5 (2C), 30.7 (2C), 31.2 (2C), 59.1 (2C), 114.8 (4C), 121.6 (4C), 129.3 (4C), 137.8 (2C), 147.0 (4C), 151.9 (2C), 169.1 (2C) ppm; *m*/*z* (%): 340 (77), 263 (100), 185 (26), 157 (28), 135 (66), 128 (10), 71 (34), 57 (53), 51 (25), 43 (86); *ν*_max_, (KBr): 3454, 2927, 2854, 1642, 1487, 1284, 1201, 1007, 888, 845 cm^−1^.

##### 4,4′-[Biphenyl-4,4′-diylbis(oxy)]bis(1-octylpyridinium)diiodide (11, 4BP4BO-8,I)

C_38_H_50_I_2_N_2_O_2_; *M*_w_ 820.6; yellow solid (0.63 g, 77% yield); mp 174–177 °C; ^1^H NMR (300 MHz, DMSO-*d*_6_): *δ* 0.85 (t, *J* = 6.6 Hz, 6H, 2CH_3_), 1.17–1.42 (m, 20H, 10CH_2_), 1.79–1.93 (m, 4H, 2CH_2_), 4.49 (t, *J* = 7.3 Hz, 4H, 2CH_2_N^+^), 7.49 (d, *J* = 8.4 Hz, 4H, 4CH_Ar_), 7.62 (d, *J* = 6.9 Hz, 4H, 4CH_py_), 7.94 (d, *J* = 8.4 Hz, 4H, 4CH_Ar_), 8.95 (d, *J* = 6.9 Hz, 4H, 4CH_py_) ppm; ^13^C NMR (75 MHz, DMSO-*d*_6_): *δ* 13.8 (2C), 21.9 (2C), 25.2 (2C), 28.3 (2C), 28.4 (2C), 30.5 (2C), 31.0 (2C), 59.1 (2C), 114.8 (4C), 121.5 (4C), 129.2 (4C), 137.7 (2C), 146.8 (4C), 151.8 (2C), 169.0 (2C) ppm; *m*/*z* (%): 340 (79), 263 (63), 240 (12), 185 (20), 157 (25), 127 (16), 78 (25), 71 (68), 57 (89), 43 (100); *ν*_max_, (KBr): 3428, 2927, 2855, 1641, 1486, 1289, 1198, 1007, 889, 848 cm^−1^.

##### 4,4′-[Biphenyl-4,4′-diylbis(oxy)]bis(1-decylpyridinium)dibromide (12, 4BP4BO-10,Br)

C_42_H_58_Br_2_N_2_O_2_; *M*_w_ 782.8; white solid (0.52 g, 67% yield); mp 225–227 °C; ^1^H NMR (300 MHz, DMSO-*d*_6_): *δ* 0.85 (t, *J* = 6.6 Hz, 6H, 2CH_3_), 1.17–1.42 (m, 28H, 14CH_2_), 1.81–1.93 (m, 4H, 2CH_2_), 4.51 (t, *J* = 7.3 Hz, 4H, 2CH_2_N^+^), 7.49 (d, *J* = 8.4 Hz, 4H, 4CH_Ar_), 7.62 (d, *J* = 6.9 Hz, 4H, 4CH_py_), 7.95 (d, *J* = 8.4 Hz, 4H, 4CH_Ar_), 8.99 (d, *J* = 6.9 Hz, 4H, 4CH_py_) ppm; ^13^C NMR (75 MHz, DMSO-*d*_6_): *δ* 13.9 (2C), 22.0 (2C), 25.3 (2C), 28.3 (2C), 28.6 (2C), 28.7 (2C), 28.8 (2C), 30.6 (2C), 31.2 (2C), 59.1 (2C), 114.8 (4C), 121.5 (4C), 129.2 (4C), 137.7 (2C), 146.9 (4C), 151.8 (2C), 169.0 (2C) ppm; *m*/*z* (%): 340 (100), 263 (44), 185 (5), 157 (7), 137 (31), 85 (8), 78 (6), 69 (11), 57 (20), 43 (25); *ν*_max_, (KBr): 3451, 2923, 2854, 1640, 1486, 1289, 1200, 1006, 890, 844 cm^−1^.

##### 4,4′-[Biphenyl-4,4′-diylbis(oxy)]bis(1-decylpyridinium)diiodide (13, 4BP4BO-10,I)

C_42_H_58_I_2_N_2_O_2_; *M*_w_ 876.8; yellow solid (0.64 g, 73% yield); mp 232–236 °C; ^1^H NMR (300 MHz, DMSO-*d*_6_): *δ* 0.85 (t, *J* = 6.6 Hz, 6H, 2CH_3_), 1.17–1.42 (m, 28H, 14CH_2_), 1.81–1.93 (m, 4H, 2CH_2_), 4.49 (t, *J* = 7.3 Hz, 4H, 2CH_2_N^+^), 7.48 (d, *J* = 8.4 Hz, 4H, 4CH_Ar_), 7.62 (d, *J* = 6.9 Hz, 4H, 4CH_py_), 7.95 (d, *J* = 8.4 Hz, 4H, 4CH_Ar_), 8.94 (d, *J* = 6.9 Hz, 4H, 4CH_py_) ppm; ^13^C NMR (75 MHz, DMSO-*d*_6_): *δ* 13.9 (2C), 22.0 (2C), 25.2 (2C), 28.3 (2C), 28.6 (2C), 28.7 (2C), 28.8 (2C), 30.5 (2C), 31.2 (2C), 59.1 (2C), 114.8 (4C), 121.5 (4C), 129.2 (4C), 137.7 (2C), 146.8 (4C), 151.8 (2C), 169.0 (2C) ppm; *m*/*z* (%): 340 (94), 263 (73), 185 (11), 157 (18), 141 (12), 128 (12), 85 (52), 71 (53), 57 (100), 43 (98); *ν*_max_, (KBr): 3451, 2924, 2848, 1642, 1485, 1285, 1199, 1009, 889, 843 cm^−1^.

##### 4,4′-[Biphenyl-4,4′-diylbis(oxy)]bis(1-dodecylpyridinium)dibromide (14, 4BP4BO-12,Br)

C_46_H_66_Br_2_N_2_O_2_; *M*_w_ 838.9; white solid (0.59 g, 70% yield); mp 248–251 °C; ^1^H NMR (300 MHz, DMSO-*d*_6_): *δ* 0.85 (t, *J* = 6.6 Hz, 6H, 2CH_3_), 1.17–1.42 (m, 36H, 18CH_2_), 1.81–1.93 (m, 4H, 2CH_2_), 4.51 (t, *J* = 7.3 Hz, 4H, 2CH_2_N^+^), 7.49 (d, *J* = 8.4 Hz, 4H, 4CH_Ar_), 7.62 (d, *J* = 6.9 Hz, 4H, 4CH_py_), 7.95 (d, *J* = 8.4 Hz, 4H, 4CH_Ar_), 8.99 (d, *J* = 6.9 Hz, 4H, 4CH_py_) ppm; ^13^C NMR (75 MHz, DMSO-*d*_6_): *δ* 13.9 (2C), 22.0 (2C), 25.3 (2C), 28.4 (2C), 28.6 (2C), 28.7 (2C), 28.8 (4C), 28.9 (2C), 30.6 (2C), 31.2 (2C), 59.0 (2C), 114.7 (4C), 121.5 (4C), 129.2 (4C), 137.7 (2C), 146.9 (4C), 151.8 (2C), 169.0 (2C) ppm; 340 (100), 263 (52), 185 (6), 157 (13), 135 (5), 128 (10), 71 (34), 57 (53), 51 (15), 43 (86); *ν*_max_, (KBr): 3478, 2920, 2853, 1641, 1484, 1293, 1203, 1007, 889, 833 cm^−1^.

##### 4,4′-[Biphenyl-4,4′-diylbis(oxy)]bis(1-dodecylpyridinium)diiodide (15, 4BP4BO-12,I)

C_46_H_66_I_2_N_2_O_2_; *M*_w_ 932.9; yellow solid (0.76 g, 82% yield); mp 225–228 °C; ^1^H NMR (300 MHz, DMSO-*d*_6_): *δ* 0.85 (t, *J* = 6.6 Hz, 6H, 2CH_3_), 1.17–1.42 (m, 36H, 18CH_2_), 1.81–1.93 (m, 4H, 2CH_2_), 4.49 (t, *J* = 7.3 Hz, 4H, 2CH_2_N^+^), 7.49 (d, *J* = 8.4 Hz, 4H, 4CH_Ar_), 7.62 (d, *J* = 6.9 Hz, 4H, 4CH_py_), 7.95 (d, *J* = 8.4 Hz, 4H, 4CH_Ar_), 8.95 (d, *J* = 6.9 Hz, 4H, 4CH_py_) ppm; ^13^C NMR (75 MHz, DMSO-*d*_6_): *δ* 13.9 (2C), 22.0 (2C), 25.3 (2C), 28.3 (2C), 28.6 (2C), 28.7 (2C), 28.8 (4C), 28.9 (2C), 30.5 (2C), 31.2 (2C), 59.1 (2C), 114.8 (4C), 121.5 (4C), 129.2 (4C), 137.7 (2C), 146.8 (4C), 151.8 (2C), 169.0 (2C) ppm; *m*/*z* (%): 340 (15), 263 (40), 185 (9), 157 (11), 128 (12), 85 (36), 78 (11), 71 (34), 57 (85), 43 (100); *ν*_max_, (KBr): 3429, 2922, 2850, 1641, 1486, 1279, 1202, 1013, 887, 844 cm^−1^.

#### Preparation of 4,4′-diacetyldiphenyl ether

The mixture of diphenyl ether 16 (10.20 g, 60 mmol), acetyl chloride (9.42 g, 120 mmol) and DCM (50 mL) was slowly added to the suspension of aluminium chloride (9.42 g, 120 mmol) in DCM (50 mL) at 0 °C. After stirring for 1 d at rt, water (250 mL) was added. The resulting solution was extracted with chloroform (3 × 100 mL). The combined organic layers was washed with saturated aqueous sodium bicarbonate (50 mL) and dried over sodium sulfate. Then solvent was removed under reduced pressure to give 4,4′-diacetyldiphenyl ether (12.8 g, 84% yield).

C_16_H_14_O_3_; *M*_w_ 254.3; white solid; mp 99–101 °C; ^1^H NMR (300 MHz, CDCl_3_): *δ* 2.61 (s, 6H, 2CH_3_), 7.10 (d, *J* = 8.8 Hz, 4H, 4CH_Ar_), 8.01 (d, *J* = 8.8 Hz, 4H, 4CH_Ar_) ppm.

#### Preparation of 4,4′-diacetoxydiphenyl ether

The mixture of 4,4′-diacetyldiphenyl ether (7.62 g, 30 mmol), mCPBA (30.4 g, 176 mmol) and DCM (150 mL) was stirred for 5 h at rt. The resulting solution was filtered. Filtrate was washed with aqueous sodium bicarbonate (50 mL) and dried over sodium sulfate. Then solvent was removed under reduced pressure to give 4,4′-diacetoxydiphenyl ether (7.46 g, 87% yield).

C_16_H_14_O_5_; *M*_w_ 286.3; white solid; mp 109–110 °C; ^1^H NMR (300 MHz, CDCl_3_): *δ* 2.31 (s, 6H, 2CH_3_), 7.02 (d, *J* = 9.1 Hz, 4H, 4CH_Ar_), 7.07 (d, *J* = 9.1 Hz, 4H, 4CH_Ar_) ppm.

#### Preparation of 17

##### 4,4′-Dihydroxydiphenyl ether

The 25% solution of sodium hydroxide (2.0 g, 50 mmol) in water was slowly added to solution of 4,4′-diacetyldiphenyl ether (6.62 g, 23.2 mmol) in methanol (60 mL). After stirring for 2 h solvent was removed under reduced pressure. Then water was added to crude residue, and resulting solution was treated with hydrochloric acid until the end of precipitation. Then mixture was filtered. The solid remaining on the filter was washed with 100 mL of water, refluxed in hexane (100 mL), filtered off and dried to provide 4,4′-dihydroxydiphenyl ether (17) (4.22 g, 90% yield).

C_12_H_10_O_3_; *M*_w_ 202.21; white solid; mp 162–164 °C; ^1^H NMR (300 MHz, CDCl_3_): 6.71 (d, *J* = 9.0 Hz, 4H, 4CH_Ar_), 6.77 (d, *J* = 9.0 Hz, 4H, 4CH_Ar_), 9.16 (s, 1H, OH) ppm.

#### Preparation of 18

##### 2,2′-[Oxybis(4,1-phenyleneoxy)]dipyridine (2OB4POB)

The mixture of 4,4′-dihydroxydiphenyl ether (17) (2.02 g, 10 mmol), 2-bromopyridine (3.16 g, 20 mmol), potassium phosphate (8.48 g, 40 mmol), copper(i) iodide (1.90 g, 10 mmol) and picolinic acid (0.25 g, 2 mmol) in dry DMSO (50 mL) was heated to 90 °C for 24 hours in argon atmosphere. Solvent was removed under reduced pressure, ethyl acetate (50 mL) was added to crude residue, and the mixture was heated to reflux for 1 hour. Then mixture was filtered off. The organic filtrate was concentrated under reduced pressure and the residue was purified by recrystallization from heptane to afford 18 (2.37 g, 65% yield).

C_22_H_16_N_2_O_3_; *M*_w_ 356.4; white solid; mp 95–98 °C; ^1^H NMR (300 MHz, CDCl_3_): *δ* 6.93 (d, *J* = 8.5 Hz, 2H, 2CH_py_), 7.01 (t, *J* = 6.4 Hz, 2H, 2CH_py_), 7.08 (d, *J* = 9.1 Hz, 4H, CH_Ar_), 7.14 (d, *J* = 9.1 Hz, 4H, CH_Ar_), 7.70 (t, *J* = 8.5 Hz, 2H, 2CH_py_), 8.22 (d, *J* = 6.4 Hz, 2H, 2CH_py_) ppm; ^13^C NMR (75 MHz, DMSO-*d*_6_): *δ* 111.2 (2C), 118.9 (2C), 119.5 (4C), 122.7 (4C), 140.1 (2C), 147.3 (2C), 149.3 (2C), 153.4 (2C), 163.1 (2C) ppm.

#### Preparation of mixture 19 + 20

Decyl bromide (10 mmol) was added to a solution of 2,2′-[oxybis(4,1-phenyleneoxy)]dipyridine (18) (0.36 g, 1 mmol) in acetonitrile (3 mL). The mixture was heated under reflux for 7 days, then allowed to cool to room temperature and filtered off. The filtered solid was washed with 5 mL of cold acetone and dried to give a mixture of mono- 19 and bis-QAC 20 with ratio 1 : 2 (by NMR).

#### Preparation of 21

##### 4,4′-[Oxybis(4,1-phenyleneoxy)]dipyridine (4OB4POB)

The mixture of 4,4′-dihydroxydiphenyl ether (17) (2.02 g, 10 mmol), 4-chloropyridine hydrochloride (3.00 g, 20 mmol), potassium phosphate (16.96 g, 10 mmol) and picolinic acid (0.25 g, 2 mmol) in dry DMSO (50 mL) was heated to 140 °C for 72 hours in argon atmosphere. Solvent was removed under reduced pressure, ethyl acetate (50 mL) was added to crude residue, and the mixture was heated to reflux for 1 hour. Then mixture was filtered off. The organic filtrate was concentrated under reduced pressure and the residue was purified by recrystallization in heptane to afford 4,4′-[oxybis(4,1-phenyleneoxy)]dipyridine (21) (3.17 g, 89% yield).

C_22_H_16_N_2_O_3_; *M*_w_ 356.4; white solid; mp 104–106 °C; ^1^H NMR (300 MHz, CDCl_3_): *δ* 6.86 (d, *J* = 6.9 Hz, 4H, 4CH_py_), 7.11 (m, 8H, 8CH_Ar_), 8.50 (d, *J* = 6.9 Hz, 4H, 4CH_py_) ppm; ^13^C NMR (75 MHz, CDCl_3_): *δ* 121.0 (4C), 128.6 (4C), 136.4 (4C), 138.3 (2C), 151.7 (2C), 153.2 (4C), 163.7 (2C) ppm. ^13^C NMR (75 MHz, CDCl_3_): *δ* 112.0 (2C), 120.3 (4C), 122.3 (4C), 149.6 (2C), 151.5 (4C), 154.5 (4C), 164.9 (2C) ppm.

#### Preparations of 22–27

Alkyl halide (2.2 mmol) was added to a solution of 4,4′-[biphenyl-4,4′-diylbis(oxy)]dipyridin (21) (0.36 g, 1 mmol) in acetonitrile (3 mL). The mixture was heated under reflux for 72 h, then allowed to cool to room temperature and filtered off. The solid was washed with 10 mL of cold acetone and dried to give a bis-QAC. The yields of bis-QACs 22–27 were 65–82% depend on alkyl halide.

##### 4,4′-[Oxybis(4,1-phenyleneoxy)]bis(1-octylpyridinium)dibromide (22, 4OB4POB-8,Br)

C_38_H_50_Br_2_N_2_O_3_; *M*_w_ 742.6; white solid (0.56 g, 75% yield); mp 68–71 °C; ^1^H NMR (300 MHz, DMSO-*d*_6_): *δ* 0.85 (t, *J* = 6.6 Hz, 6H, 2CH_3_), 1.17–1.42 (m, 20H, 10CH_2_), 1.81–1.93 (m, 4H, 2CH_2_), 4.50 (t, *J* = 7.0 Hz, 4H, 2CH_2_N^+^), 7.29 (d, *J* = 8.4 Hz, 4H, 4CH_Ar_), 7.42 (d, *J* = 8.4 Hz, 4H, 4CH_Ar_), 7.61 (d, *J* = 6.9 Hz, 4H, 4CH_py_), 8.96 (d, *J* = 6.9 Hz, 4H, 4CH_py_) ppm; ^13^C NMR (75 MHz, DMSO-*d*_6_): *δ* 14.0 (2C), 22.1 (2C), 25.4 (2C), 28.5 (2C), 28.6 (2C), 30.7 (2C), 31.3 (2C), 59.1 (2C), 114.7 (4C), 121.0 (4C), 122.9 (4C), 147.0 (4C), 147.8 (2C), 155.1 (2C), 169.5 (2C) ppm; *m*/*z* (%): 356 (100), 279 (28), 171 (4), 137 (30), 85 (6), 78 (7), 69 (5), 55 (10), 43 (13), 29 (4); *ν*_max_, (KBr): 3423, 2926, 2855, 1641, 1486, 1294, 1241, 1182, 883, 852 cm^−1^.

##### 4,4′-[Oxybis(4,1-phenyleneoxy)]bis(1-nonylpyridinium)dibromide (23, 4OB4POB-9,Br)

C_40_H_54_Br_2_N_2_O_3_; *M*_w_ 770.7; white solid (0.55 g, 72% yield); mp 78–82 °C; ^1^H NMR (300 MHz, DMSO-*d*_6_): *δ* 0.85 (t, *J* = 6.6 Hz, 6H, 2CH_3_), 1.17–1.42 (m, 24H, 12CH_2_), 1.81–1.93 (m, 4H, 2CH_2_), 4.50 (t, *J* = 7.0 Hz, 4H, 2CH_2_N^+^), 7.29 (d, *J* = 8.4 Hz, 4H, 4CH_Ar_), 7.42 (d, *J* = 8.4 Hz, 4H, 4CH_Ar_), 7.61 (d, *J* = 6.9 Hz, 4H, 4CH_py_), 8.96 (d, *J* = 6.9 Hz, 4H, 4CH_py_) ppm; ^13^C NMR (75 MHz, DMSO-*d*_6_): *δ* 14.0 (2C), 22.1 (2C), 25.4 (2C), 28.5 (2C), 28.6 (2C), 28.8 (2C), 30.7 (2C), 31.3 (2C), 59.1 (2C), 114.7 (4C), 121.0 (4C), 122.9 (4C), 147.0 (4C), 147.8 (2C), 155.1 (2C), 169.5 (2C) ppm; *m*/*z* (%): 356 (100), 279 (29), 171 (4), 137 (22), 85 (6), 78 (3), 69 (7), 55 (10), 43 (13), 29 (8); *ν*_max_, (KBr): 3424, 2926, 2855, 1641, 1488, 1293, 1241, 1182, 883, 852 cm^−1^.

##### 4,4′-[Oxybis(4,1-phenyleneoxy)]bis(1-decylpyridinium)dibromide (24, 4OB4POB-10,Br)

C_42_H_58_Br_2_N_2_O_3_; *M*_w_ 798.8; white solid (0.60 g, 75% yield); mp 93–104 °C; ^1^H NMR (300 MHz, DMSO-*d*_6_): *δ* 0.85 (t, *J* = 6.6 Hz, 6H, 2H_3_), 1.17–1.42 (m, 28H, 14CH_2_), 1.81–1.93 (m, 4H, 2CH_2_), 4.50 (t, *J* = 7.0 Hz, 4H, 2CH_2_N^+^), 7.29 (d, *J* = 8.4 Hz, 4H, 4CH_Ar_), 7.42 (d, *J* = 8.4 Hz, 4H, 4CH_Ar_), 7.61 (d, *J* = 6.9 Hz, 4H, 4CH_py_), 8.96 (d, *J* = 6.9 Hz, 4H, 4CH_py_) ppm; ^13^C NMR (75 MHz, DMSO-*d*_6_): *δ* 14.0 (2C), 22.1 (2C), 25.4 (2C), 28.5 (2C), 28.7 (2C), 28.8 (2C), 28.9 (2C), 30.7 (2C), 31.3 (2C), 59.1 (2C), 114.7 (4C), 121.0 (4C), 122.9 (4C), 147.0 (4C), 147.8 (2C), 155.1 (2C), 169.5 (2C) ppm; *m*/*z* (%): 356 (100), 279 (29), 151 (6), 137 (36), 85 (6), 69 (5), 55 (8), 43 (11), 29 (7), 18 (6); *ν*_max_, (KBr): 3422, 2925, 2854, 1641, 1488, 1294, 1242, 1183, 883, 852 cm^−1^.

##### 4,4′-[Oxybis(4,1-phenyleneoxy)]bis(1-decylpyridinium)dichloride (25, 4OB4POB-10,Cl)

C_42_H_58_Cl_2_N_2_O; *M*_w_ 709.8; white solid (0.46 g, 65% yield); mp 90–93 °C; ^1^H NMR (300 MHz, DMSO-*d*_6_): *δ* 0.85 (t, *J* = 6.6 Hz, 6H, 2CH_3_), 1.17–1.42 (m, 28H, 14CH_2_), 1.81–1.93 (m, 4H, 2CH_2_), 4.50 (t, *J* = 7.0 Hz, 4H, 2CH_2_N^+^), 7.29 (d, *J* = 8.4 Hz, 4H, 4CH_Ar_), 7.42 (d, *J* = 8.4 Hz, 4H, 4CH_Ar_), 7.61 (d, *J* = 6.9 Hz, 4H, 4CH_py_), 8.96 (d, *J* = 6.9 Hz, 4H, 4CH_py_) ppm; ^13^C NMR (75 MHz, DMSO-*d*_6_): *δ* 14.0 (2C), 22.1 (2C), 25.4 (2C), 28.5 (2C), 28.7 (2C), 28.8 (2C), 28.9 (2C), 30.7 (2C), 31.3 (2C), 59.1 (2C), 114.7 (4C), 121.0 (4C), 122.9 (4C), 147.0 (4C), 147.8 (2C), 155.1 (2C), 169.5 (2C) ppm; *m*/*z* (%): 356 (100), 279 (25), 149 (10), 135 (34), 78 (9), 69 (8), 57 (19), 51 (13), 43 (25), 29 (9); *ν*_max_, (KBr): 3424, 2926, 2855, 1641, 1488, 1293, 1241, 1182, 883, 852 cm^−1^.

##### 4,4′-[Oxybis(4,1-phenyleneoxy)]bis(1-decylpyridinium)diiodide (26, 4OB4POB-10,I)

C_42_H_58_I_2_N_2_O_3_; *M*_w_ 892.8; yellow solid (0.73 g, 82% yield); mp 134–138 °C; ^1^H NMR (300 MHz, DMSO-*d*_6_): *δ* 0.85 (t, *J* = 6.6 Hz, 6H, 2CH_3_), 1.17–1.42 (m, 28H, 14CH_2_), 1.81–1.93 (m, 4H, 2CH_2_), 4.48 (t, *J* = 7.0 Hz, 4H, 2CH_2_N^+^), 7.29 (d, *J* = 8.4 Hz, 4H, 4CH_Ar_), 7.42 (d, *J* = 8.4 Hz, 4H, 4CH_Ar_), 7.59 (d, *J* = 6.9 Hz, 4H, 4CH_py_), 8.92 (d, *J* = 6.9 Hz, 4H, 4CH_py_) ppm; ^13^C NMR (75 MHz, DMSO-*d*_6_): *δ* 13.9 (2C), 22.0 (2C), 25.3 (2C), 28.3 (2C) 28.5 (2C), 28.7 (2C), 28.8 (2C), 30.5 (2C), 31.2 (2C), 59.1 (2C), 114.6 (4C), 120.8 (4C), 122.7 (4C), 146.7 (4C), 147.7 (2C), 154.9 (2C), 169.4 (2C) ppm; *m*/*z* (%): 356 (100), 279 (27), 268 (9), 155 (10), 141 (10), 85 (20), 71 (39), 57 (30), 43 (28), 29 (8); *ν*_max_, (KBr): 3442, 3032, 2922, 2853, 1641, 1489, 1297, 1242, 1183, 849 cm^−1^.

##### 4,4′-[Oxybis(4,1-phenyleneoxy)]bis(1-undecylpyridinium)dibromide (27, 4OB4POB-11,Br)

C_44_H_62_Br_2_N_2_O_3_; *M*_w_ 826.8; white solid (0.64 g, 78% yield); mp 87–94 °C; ^1^H NMR (300 MHz, DMSO-*d*_6_): *δ* 0.85 (t, *J* = 6.6 Hz, 6H, 2CH_3_), 1.17–1.35 (m, 32H, 16CH_2_), 1.81–1.93 (m, 4H, 2CH_2_), 4.50 (t, *J* = 7.0 Hz, 4H, 2CH_2_N^+^), 7.29 (d, *J* = 8.4 Hz, 4H, 4CH_Ar_), 7.42 (d, *J* = 8.4 Hz, 4H, 4CH_Ar_), 7.61 (d, *J* = 6.9 Hz, 4H, 4CH_py_), 8.96 (d, *J* = 6.9 Hz, 4H, 4CH_py_) ppm; ^13^C NMR (75 MHz, DMSO-*d*_6_): *δ* 13.8 (2C), 22.0 (2C), 25.2 (2C), 28.3 (2C), 28.6 (6C), 28.8 (2C), 30.5 (2C), 31.2 (2C), 59.0 (2C), 114.6 (4C), 120.8 (4C), 122.7 (4C), 146.8 (4C), 147.7 (2C), 154.9 (2C), 169.3 (2C) ppm; *m*/*z* (%): 356 (100), 279 (21), 149 (10), 135 (34), 78 (9), 69 (10), 57 (17), 51 (13), 43 (25), 29 (9); *ν*_max_, (KBr): 3424, 3024, 2924, 2853, 1640, 1488, 1294, 1242, 1181, 849 cm^−1^.

### Biology

#### Sample preparation

Samples were provided by the collaborator and stored frozen at −20 °C. Samples were prepared in DMSO and water to a final testing concentration of 32 μg mL^−1^ or 20 μM (unless otherwise indicated in the data sheet) and serially diluted 1 : 2 fold for 8 times. Each sample concentration was prepared in 384-well plates, non-binding surface plate (NBS; Corning 3640) for each bacterial/fungal strain, tissue-culture treated (TC-treated; Corning 3712/3764) black for mammalian cell types and polypropylene 384-well (PP; Corning 3657) for haemolysis assays, all in duplicate (*n* = 2), and keeping the final DMSO concentration to a maximum of 0.5%. All the sample preparation was done using liquid handling robots.

#### Antibacterial assay

##### Procedure

All bacteria were cultured in Cation-Adjusted Mueller Hinton Broth (CAMHB) at 37 °C overnight. A sample of each culture was then diluted 40-fold in fresh broth and incubated at 37 °C for 1.5–3 h. The resultant mid-log phase cultures were diluted (CFU per mL measured by OD_600_), then added to each well of the compound containing plates, giving a cell density of 5 × 10^5^ CFU per mL and a total volume of 50 μL. All the plates were covered and incubated at 37 °C for 18 h without shaking.

##### Analysis

Inhibition of bacterial growth was determined measuring absorbance at 600 nm (OD_600_), using a Tecan M1000 Pro monochromator plate reader. The percentage of growth inhibition was calculated for each well, using the negative control (media only) and positive control (bacteria without inhibitors) on the same plate as references. The percentage of growth inhibition was calculated for each well, using the negative control (media only) and positive control (bacteria without inhibitors) on the same plate. The MIC was determined as the lowest concentration at which the growth was fully inhibited, defined by an inhibition ≥ 80%. In addition, the maximal percentage of growth inhibition is reported as *D*_Max_, indicating any compounds with partial activity. Hits were classified by MIC ≤ 16 μg mL^−1^ or MIC ≤ 10 μM in either replicate (*n* = 2 on different plates).

#### Antifungal assay

##### Procedure

Fungi strains were cultured for 3 days on Yeast Extract-Peptone Dextrose (YPD) agar at 30 °C. A yeast suspension of 1 × 10^6^ to 5 × 10^6^ CFU per mL (as determined by OD_530_) was prepared from five colonies. The suspension was subsequently diluted and added to each well of the compound-containing plates giving a final cell density of fungi suspension of 2.5 × 10^3^ CFU per mL and a total volume of 50 μL. All plates were covered and incubated at 35 °C for 36 h without shaking.

##### Analysis

Growth inhibition of *C. albicans* was determined measuring absorbance at 630 nm (OD_630_), while the growth inhibition of *C. neoformans* was determined measuring the difference in absorbance between 600 and 570 nm (OD_600–570_), after the addition of resazurin (0.001% final concentration) and incubation at 35 °C for 2 h. The absorbance was measured using a Biotek Multiflo Synergy HTX plate reader. In both cases, the percentage of growth inhibition was calculated for each well, using the negative control (media only) and positive control (fungi without inhibitors) on the same plate. The MIC was determined as the lowest concentration at which the growth was fully inhibited, defined by an inhibition ≥ 80% for *C. albicans* and an inhibition ≥ 70% for *C. neoformans*. Due to a higher variance in growth and inhibition, a lower threshold was applied to the data for *C. neoformans*. In addition, the maximal percentage of growth inhibition is reported as *D*_Max_, indicating any compounds with marginal activity. Hits were classified by MIC ≤ 16 μg mL^−1^ or MIC ≤ 10 μM in either replicate (*n* = 2 on different plates).

#### Cytotoxicity assay

##### Procedure

HEK293 cells were counted manually in a Neubauer haemocytometer and then plated in the 384-well plates containing the compounds to give a density of 5000 cells per well in a final volume of 50 μL. DMEM supplemented with 10% FBS was used as growth media and the cells were incubated together with the compounds for 20 h at 37 °C in 5% CO_2_.

##### Analysis

Cytotoxicity (or cell viability) was measured by fluorescence, ex: 560/10 nm, em: 590/10 nm (*F*_560/590_), after addition of 5 μL of 25 μg mL^−1^ resazurin (2.3 μg mL^−1^ final concentration) and after incubation for further 3 h at 37 °C in 5% CO_2_. The fluorescence intensity was measured using a Tecan M1000 Pro monochromator plate reader, using automatic gain calculation. CC_50_ (concentration at 50% cytotoxicity) were calculated by curve fitting the inhibition values *vs.* log(concentration) using a sigmoidal dose–response function, with variable fitting values for bottom, top and slope. In addition, the maximal percentage of cytotoxicity is reported as *D*_Max_, indicating any compounds with partial cytotoxicity. The curve fitting was implemented using Pipeline Pilot's dose–response component, resulting in similar values to curve fitting tools such as GraphPad's Prism and IDBS's XlFit. Any value with > indicate sample with no activity (low *D*_Max_ value) or samples with CC_50_ values above the maximum tested concentration (higher *D*_Max_ value). Cytotoxic samples were classified by CC_50_ ≤ 32 μg mL^−1^ or CC_50_ ≤ 10 μM in either replicate (*n* = 2 on different plates). In addition, samples were flagged as partial cytotoxic if *D*_Max_ ≥ 50%, even with CC_50_ > the maximum tested concentration.

#### Haemolysis assay

##### Procedure

Human whole blood was washed three times with 3 volumes of 0.9% NaCl and then resuspended in same to a concentration of 0.5 × 10^8^ cells per mL, as determined by manual cell count in a Neubauer haemocytometer. The washed cells were then added to the 384-well compound-containing plates for a final volume of 50 μL. After a 10 min shake on a plate shaker the plates were then incubated for 1 h at 37 °C. After incubation, the plates were centrifuged at 1000*g* for 10 min to pellet cells and debris, 25 μL of the supernatant was then transferred to a polystyrene 384-well assay plate.

##### Analysis

Haemolysis was determined by measuring the supernatant absorbance at 405 mm (OD_405_). The absorbance was measured using a Tecan M1000 Pro monochromator plate reader. HC_10_ and HC_50_ (concentration at 10% and 50% haemolysis, respectively) were calculated by curve fitting the inhibition values *vs.* log(concentration) using a sigmoidal dose–response function with variable fitting values for top, bottom and slope. In addition, the maximal percentage of haemolysis is reported as *D*_Max_, indicating any compounds with partial haemolysis. The curve fitting was implemented using Pipeline Pilot's dose–response component, resulting in similar values to curve fitting tools such as GraphPad's Prism and IDBS's XlFit. Any value with > indicate sample with no activity (low *D*_Max_ value) or samples with HC_10_ values above the maximum tested concentration (higher *D*_Max_ value). Haemolysis samples were classified by HC_10_ ≤ 32 μg mL^−1^ or HC_10_ ≤ 10 μM in either replicate (*n* = 2 on different plates). In addition, samples were flagged as partial haemolytic if *D*_Max_ ≥ 50%, even with HC_10_ > the maximum tested concentration.

## Conflicts of interest

There are no conflicts to declare.

## Supplementary Material

RA-011-D0RA08900A-s001
