# Supplementary material for: Investigations into the structure–activity relationship in gemini QACs based on biphenyl and oxydiphenyl linker
Source: RSC Adv. 2021 Jan 18;11(6):3429–38. doi: 10.1039/d0ra08900a (PMC8693992; doi:10.1039/d0ra08900a)
Supplement: RA-011-D0RA08900A-s001 [file RA-011-D0RA08900A-s001.pdf]

Electronic Supplementary Material (ESI) for RSC Advances.
This journal is © The Royal Society of Chemistry 2020

Electronic Supplementary Materials

Investigations into the structure-activity relationship in gemini QACs based on biphenyl and oxydiphenyl linker

Anatoly N. Vereshchagin,* Nikita A. Frolov, Valeria Yu. Konyuhova, Ekaterina A. Kapelistaya, Karl A. Hansford, and Mikhail P. Egorov

NMR (^{1}H, ^{13}C), FT-IR and HPLC Spectra

3, 2BP4BO-8, Br

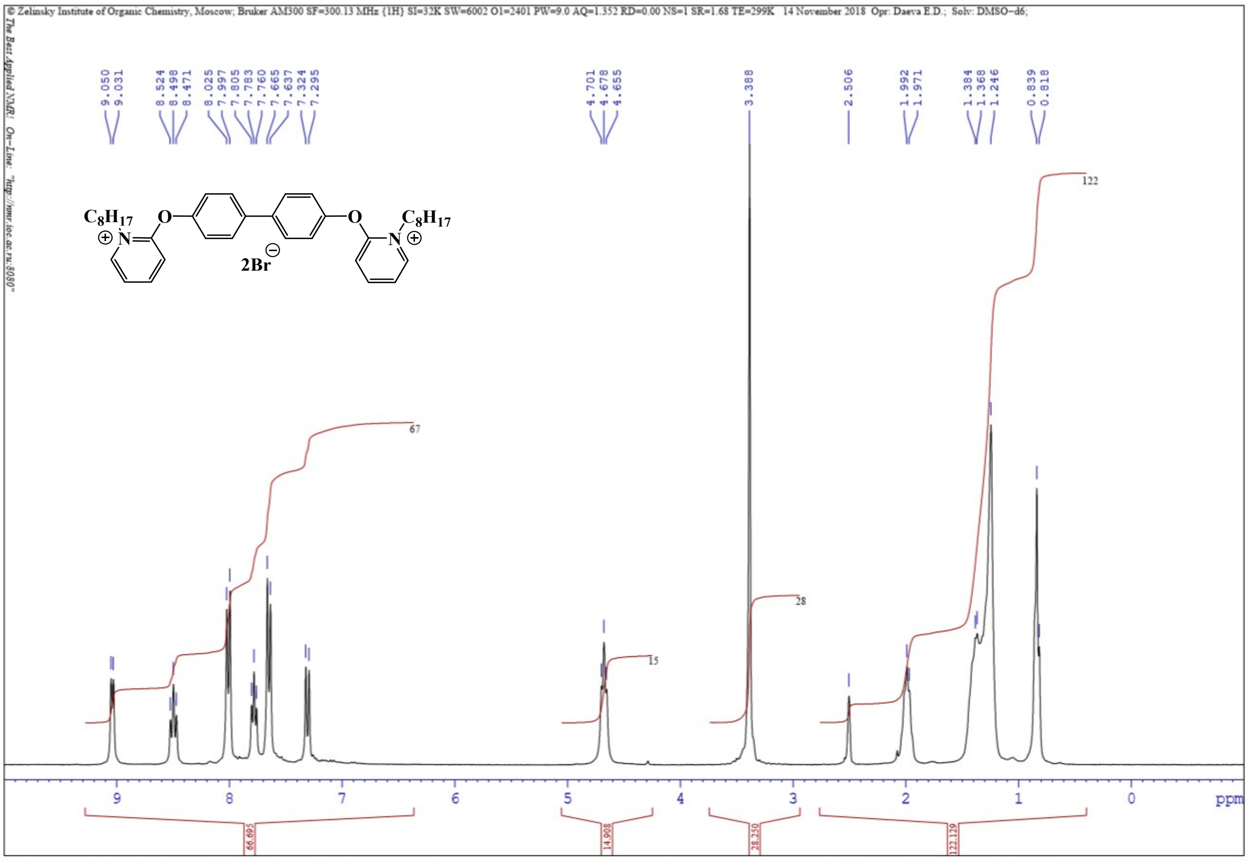

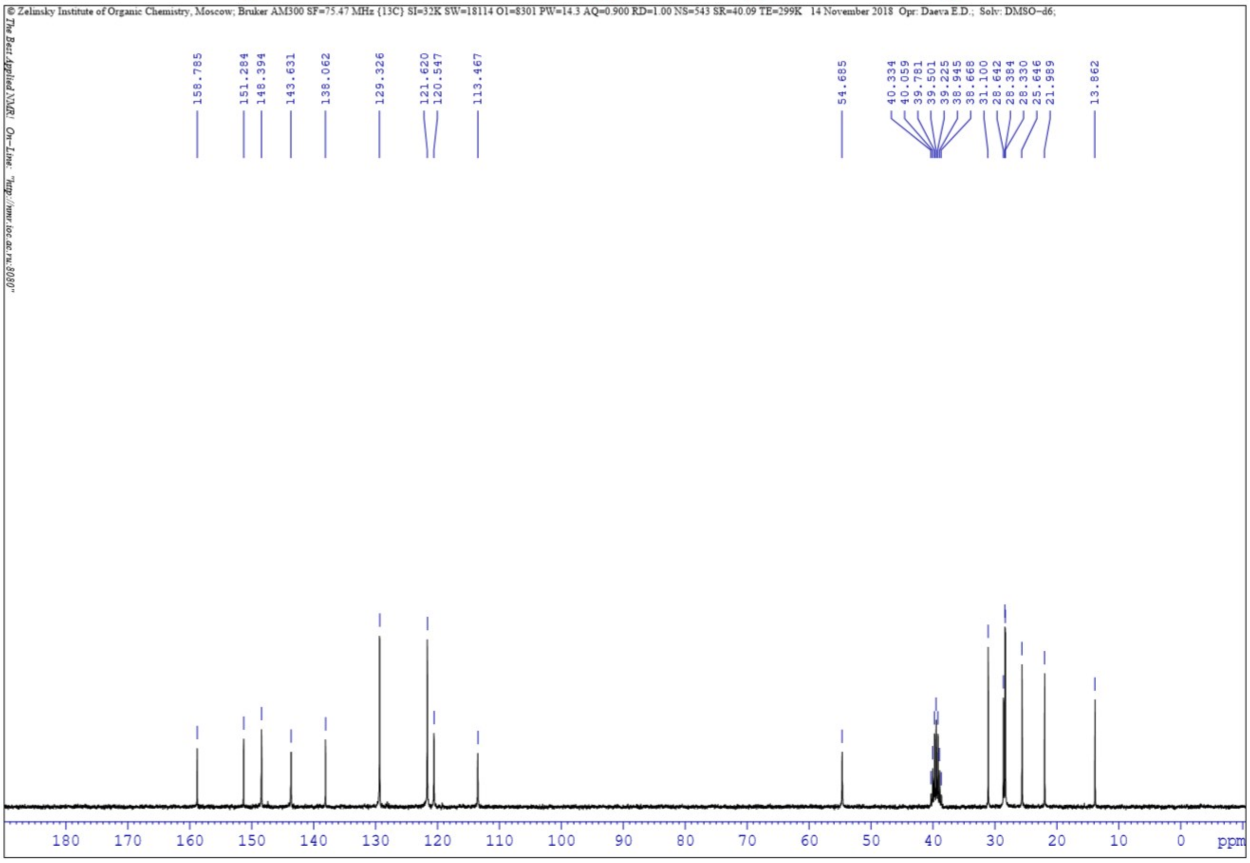

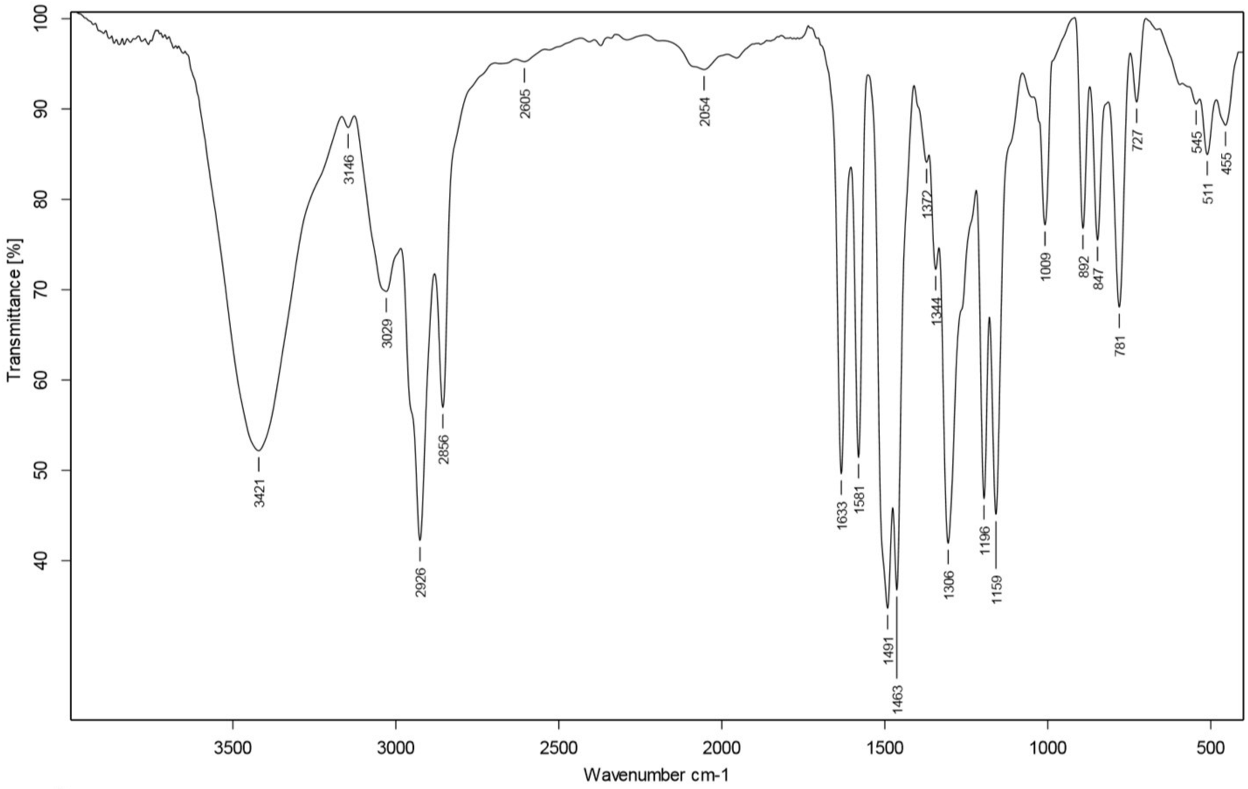

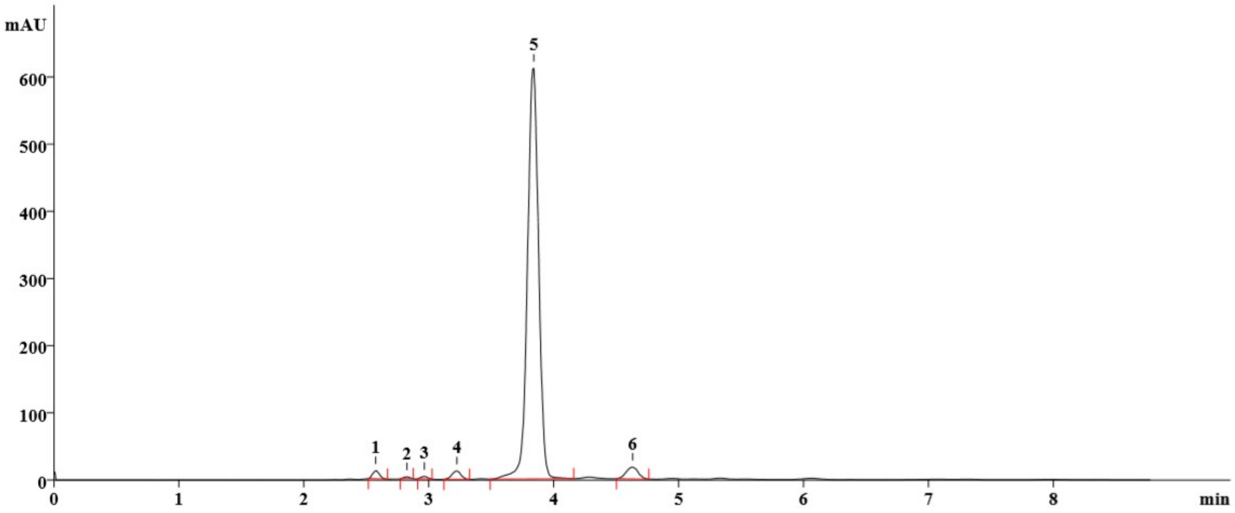

Peak table

Peak	Retention min	Area	Height	Width h/2	Area%	Type
1	2,58	45,217	11,846	0,0629	1,117	BB :
2	2,82	9,683	2,747	0,0581	0,239	BB :
3	2,97	13,578	3,612	0,0625	0,335	BB :
4	3,22	63,039	12,424	0,0794	1,557	BB :
5	3,84	3799,584	611,866	0,0958	93,870	BB :
6	4,63	116,628	17,370	0,107	2,881	BB :

4, 2BP4BO-8, I

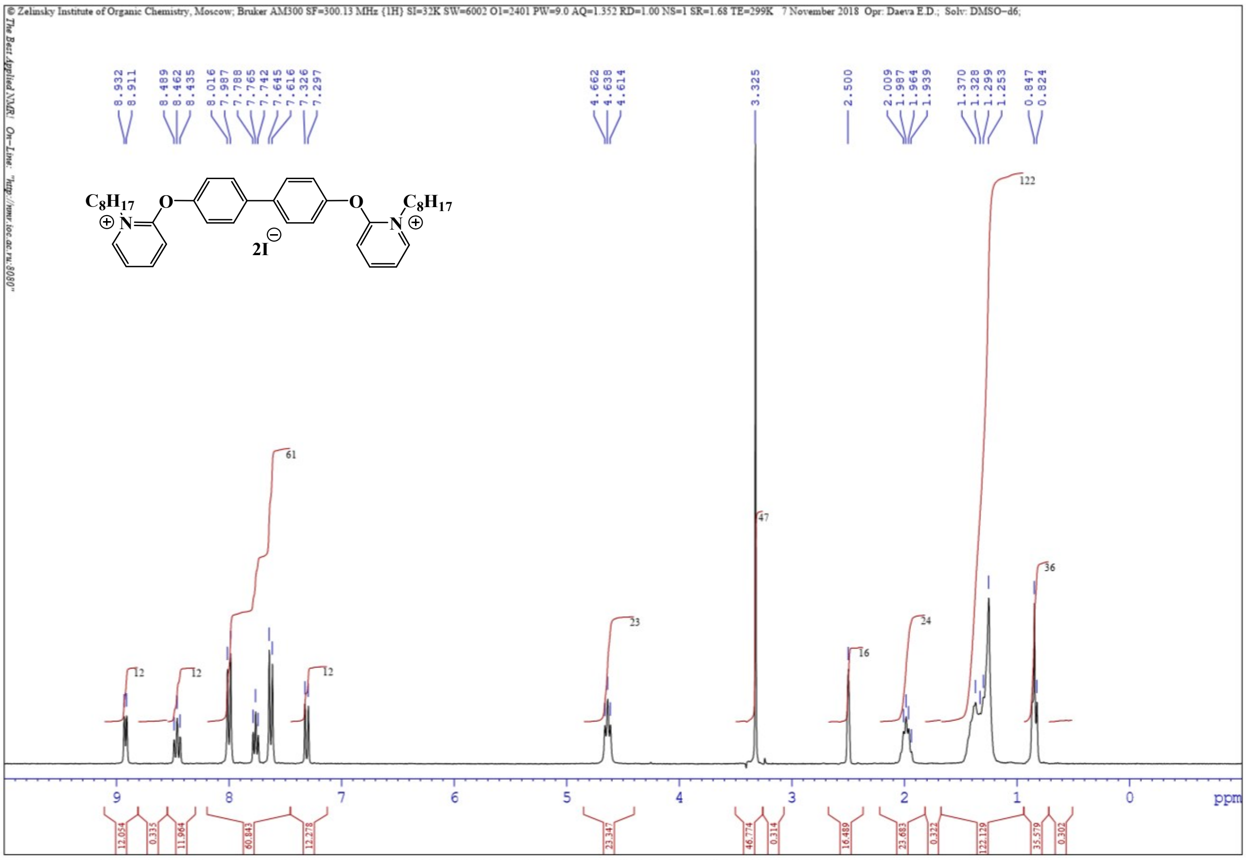
© Zelinsky Institute of Organic Chemistry, Moscow; Bruker AM300 SF=300.13 MHz {1H} SI=32K SW=6002 O1=2401 PW=9.0 AQ=1.352 RD=1.00 NS=1 SR=1.68 TE=299K 7 November 2018 Opr: Daeva E.D.; Solv: DMSO-d6;
The Best Applied NMR! On-Line: "http://nmr.ioc.ac.ru:8080"
8.932 8.911 8.489 8.462 8.435 8.016 7.987 7.788 7.765 7.742 7.645 7.616 7.326 7.297
4.662 4.638 4.614
3.325
2.500
2.009 1.987 1.964 1.939
1.370 1.328 1.299 1.253
0.847 0.824
C8H17
O
N
2I
ppm
12.054 0.335 11.964 60.843 12.278 23.347 46.774 0.314 16.489 23.683 0.322 122.129 35.579 0.302

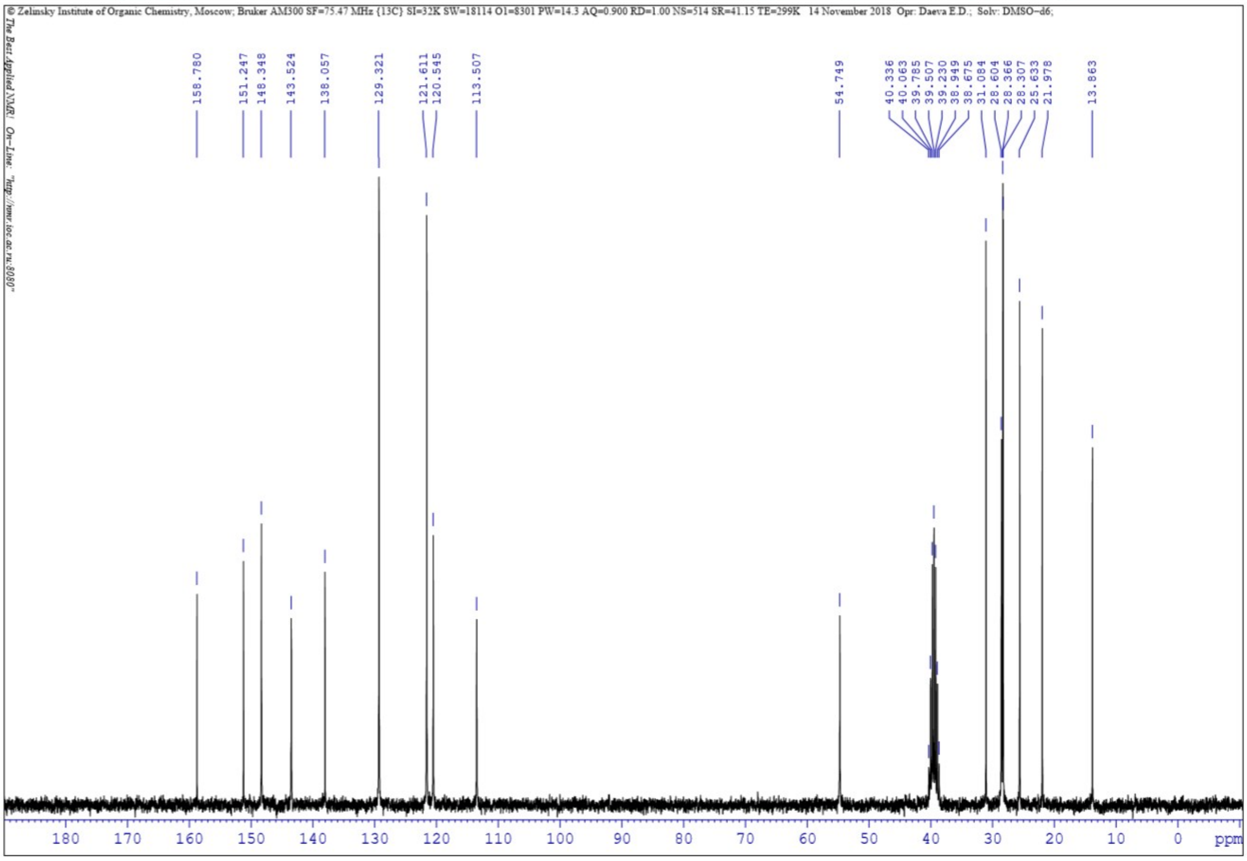
© Zelinsky Institute of Organic Chemistry, Moscow; Bruker AM300 SF=75.47 MHz {13C} SI=32K SW=18114 O1=8301 PW=14.3 AQ=0.900 RD=1.00 NS=514 SR=41.15 TE=299K 14 November 2018 Opr: Daeva E.D.; Solv: DMSO-d6;
The Best Applied NMR! On-Line: "http://nmr.ioc.ac.ru:8080"
158.780 151.247 148.348 143.524 138.057 129.321 121.611 120.545 113.507
54.749
40.336 40.063 39.785 39.507 39.230 38.949 38.675 31.084 28.604 28.366 28.307 25.633 21.978
13.863
ppm

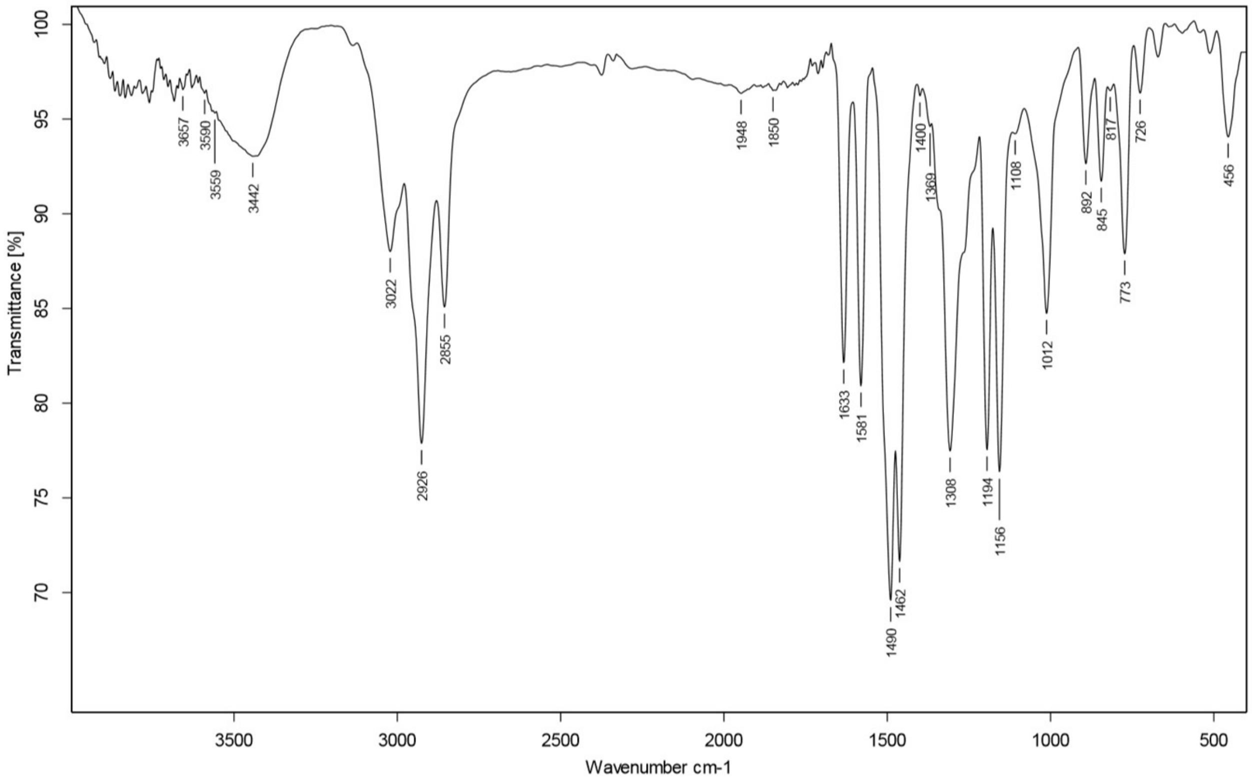

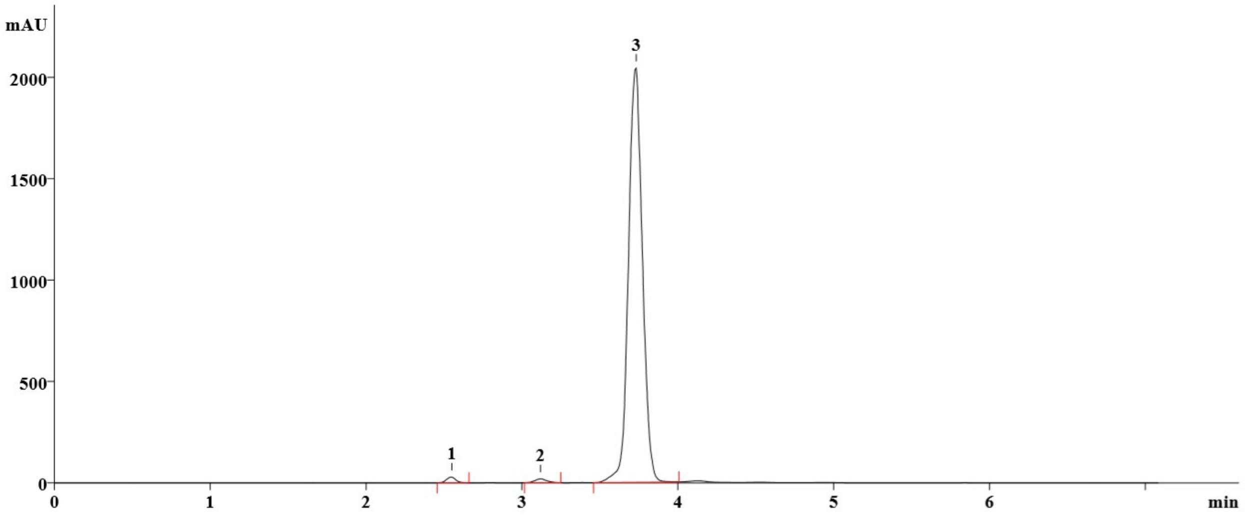

Peak table

Peak	Retention min	Area	Height	Width h/2	Area%	Type
1	2,55	114,127	27,770	0,0645	0,881	BB :
2	3,12	99,119	18,458	0,0818	0,765	BB :
3	3,73	12735,514	2042,445	0,0957	98,353	BB :

5, 2BP4BO-10, Br

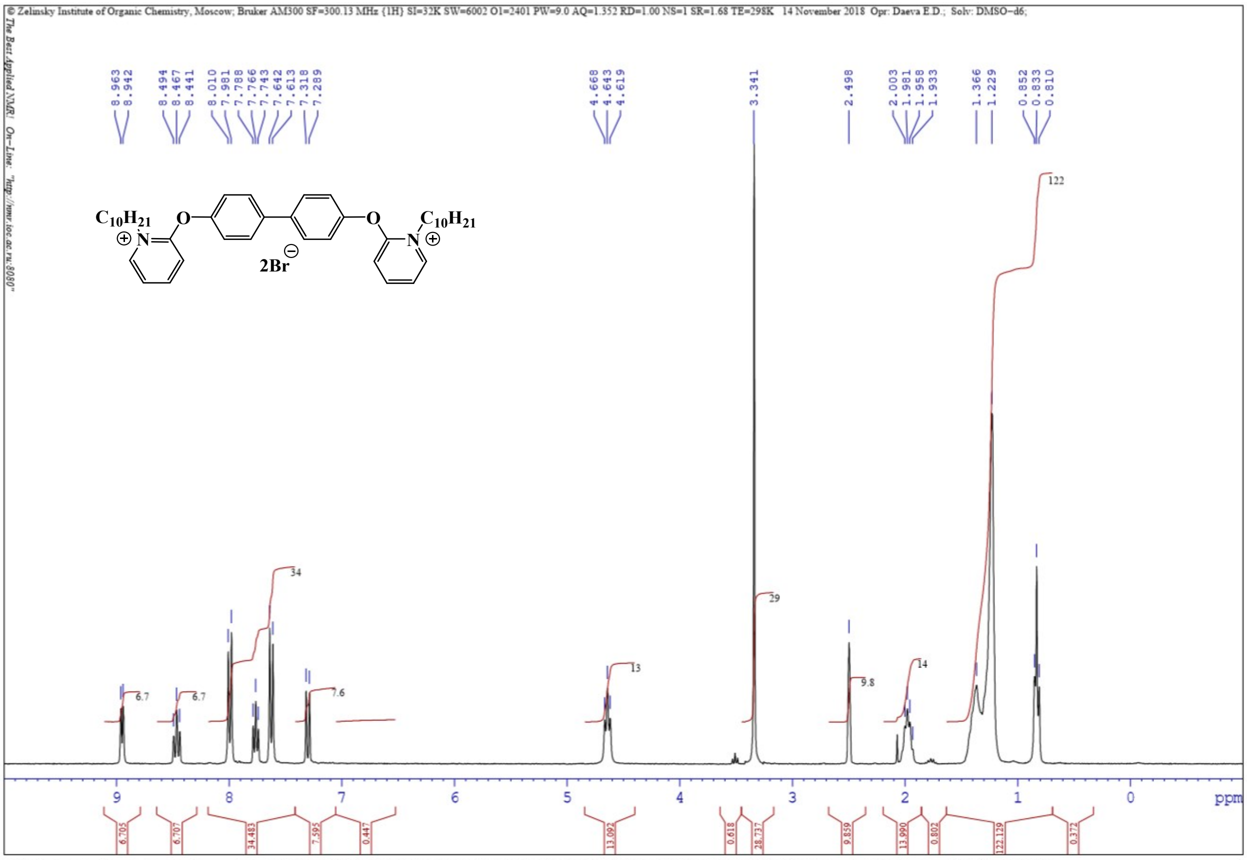

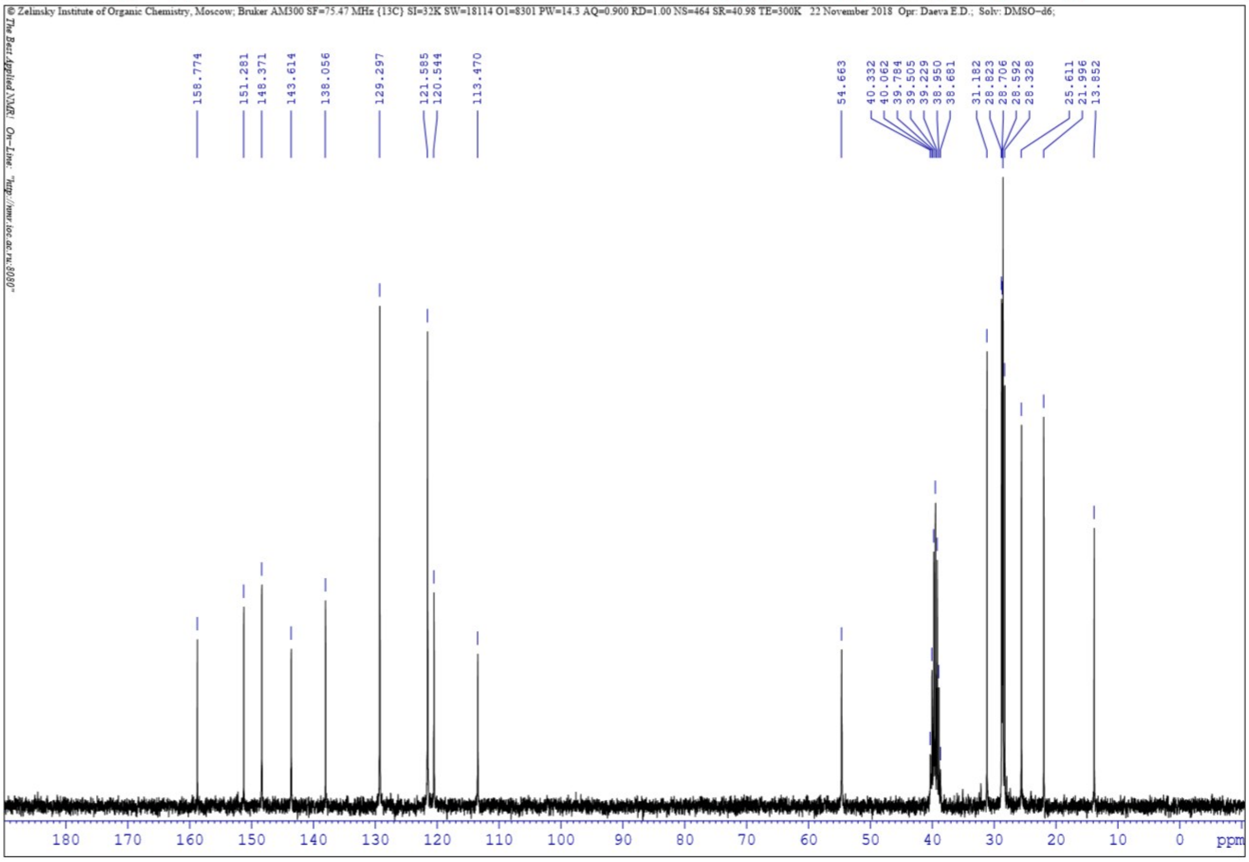

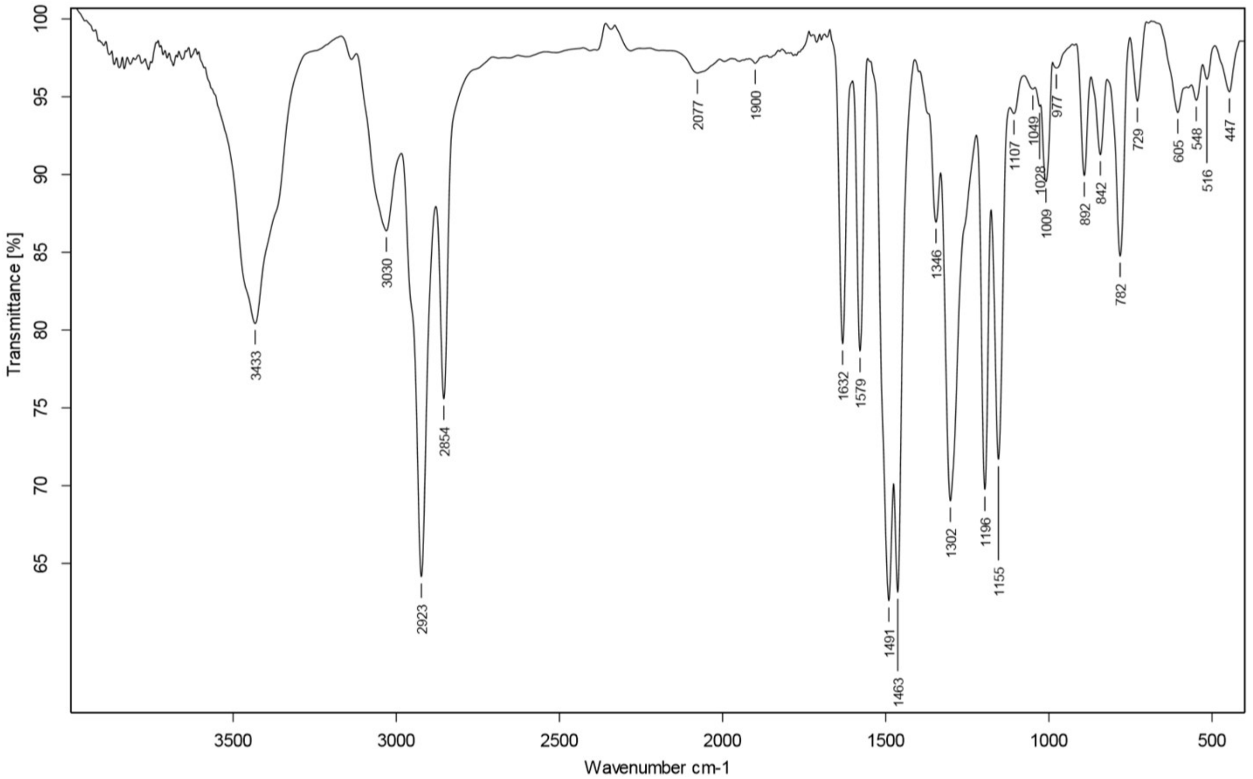

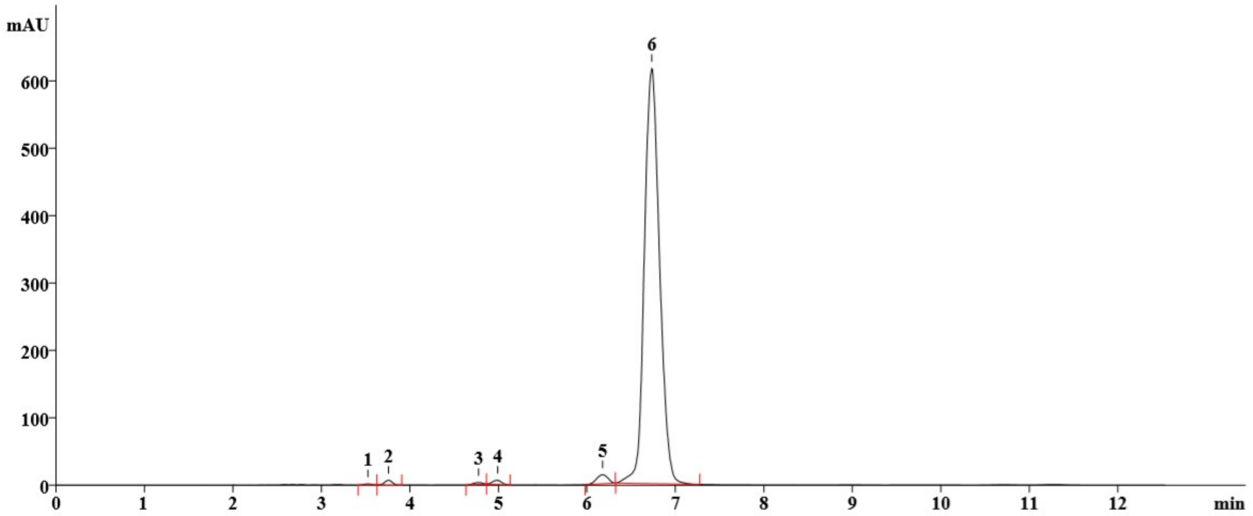

Peak table

Peak	Retention min	Area	Height	Width h/2	Area%	Type
1	3,53	8,360	1,515	0,0886	0,110	BB :
2	3,76	41,018	6,836	0,0957	0,540	BB :
3	4,77	18,502	2,706	0,114	0,244	BB :
4	4,99	43,569	6,166	0,117	0,574	BB :
5	6,18	119,203	13,265	0,146	1,570	BB :
6	6,74	7361,074	615,807	0,184	96,962	BB :

6, 2BP4BO-10, I

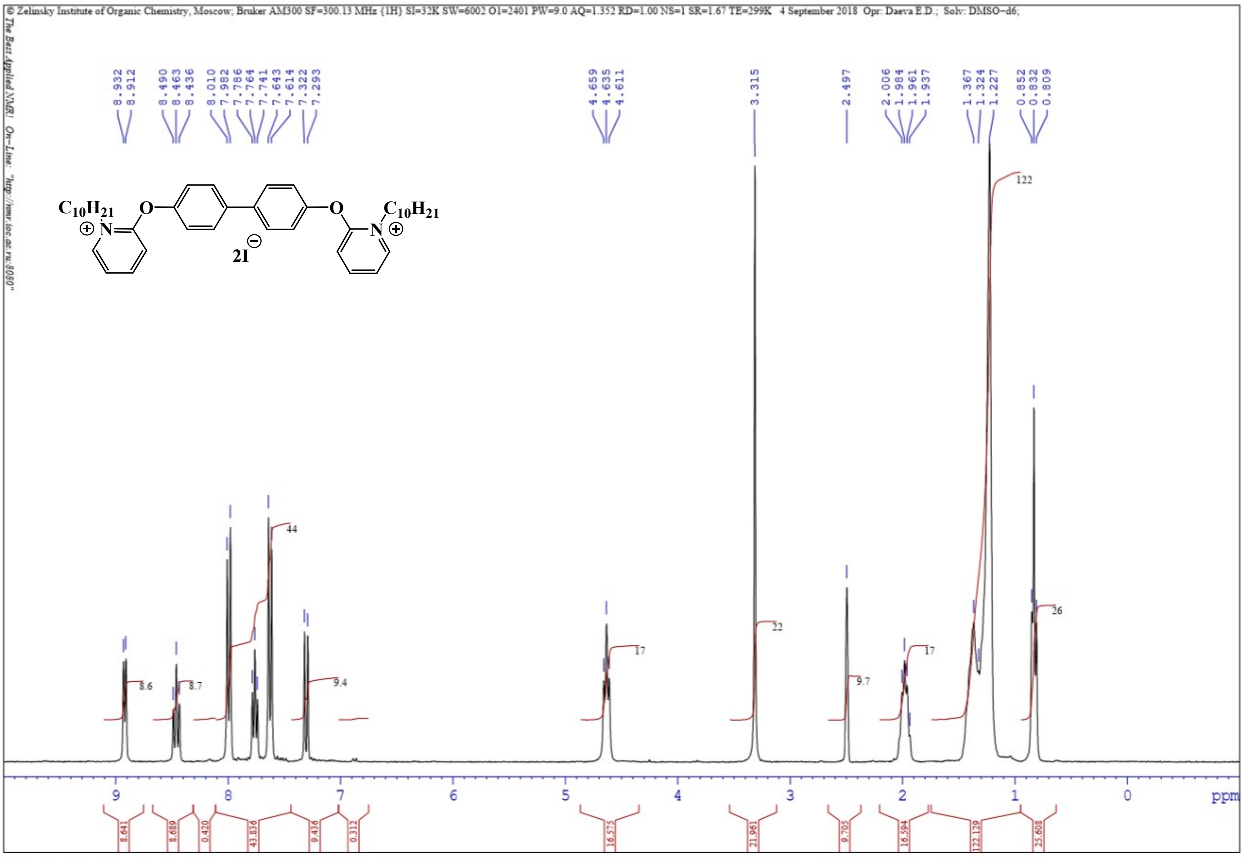
© Zelinsky Institute of Organic Chemistry, Moscow; Bruker AM300 SF=300.13 MHz {1H} SI=32K SW=6002 O1=2401 PW=9.0 AQ=1.352 RD=1.00 NS=1 SR=1.67 TE=299K 4 September 2018 Opr: Daeva E.D.; Solv: DMSO−d6;
The Best Applied NMR! On−Line: "http://nmr.ioc.ac.ru:8080"
8.932 8.912 8.490 8.463 8.436 8.010 7.982 7.786 7.764 7.741 7.643 7.614 7.322 7.293
4.659 4.635 4.611
3.315
2.497
2.006 1.984 1.961 1.937
1.367 1.324 1.227
0.852 0.832 0.809
C10H21
O
O
C10H21
N
N
2I
8.6
8.7
44
9.4
17
22
9.7
17
122
26
ppm
8.641 8.689 0.420 43.836 9.436 0.312 16.575 21.961 9.705 16.594 122.129 25.608

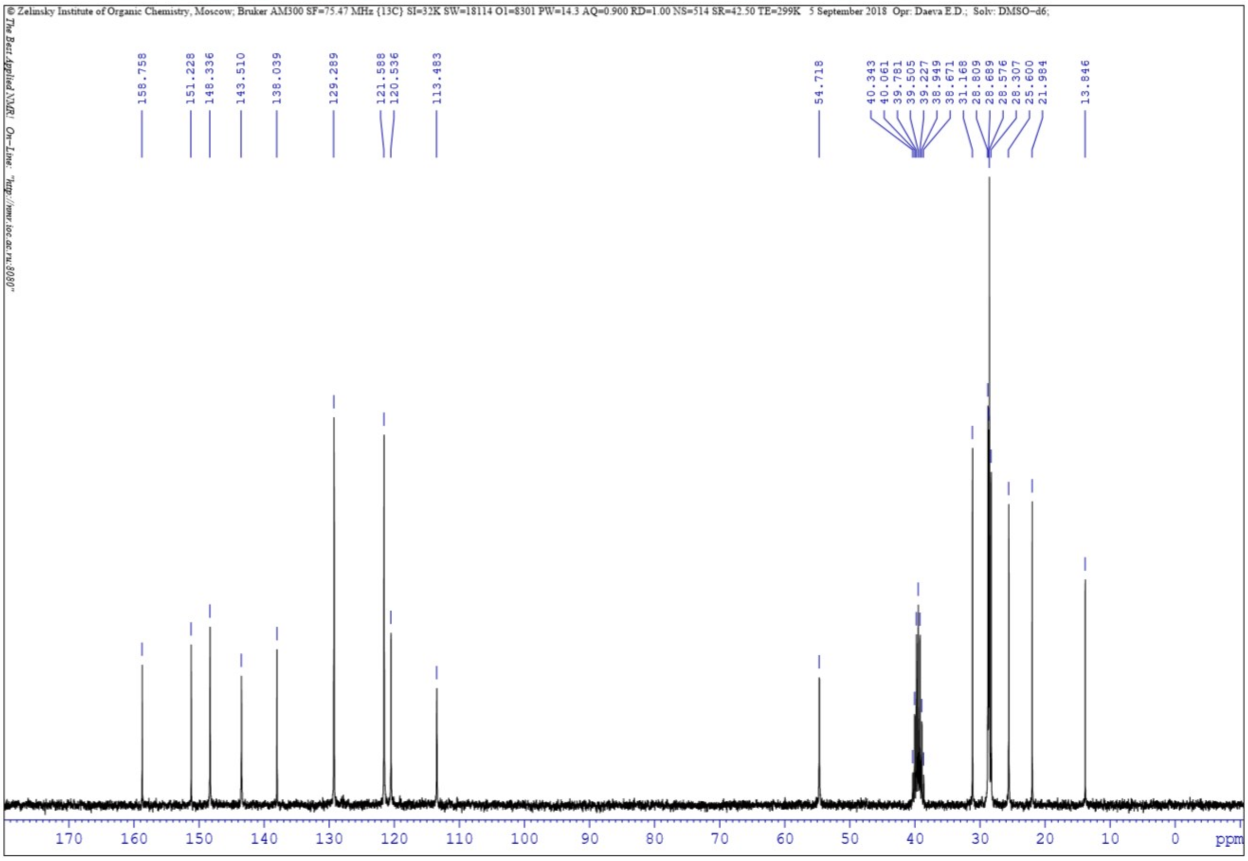
© Zelinsky Institute of Organic Chemistry, Moscow; Bruker AM300 SF=75.47 MHz {13C} SI=32K SW=18114 O1=8301 PW=14.3 AQ=0.900 RD=1.00 NS=514 SR=42.50 TE=299K 5 September 2018 Opr: Daeva E.D.; Solv: DMSO−d6;
The Best Applied NMR! On−Line: "http://nmr.ioc.ac.ru:8080"
158.758 151.228 148.336 143.510 138.039 129.289 121.588 120.536 113.483
54.718
40.343 40.061 39.781 39.505 39.227 38.949 38.671 31.168 28.809 28.689 28.576 28.307 25.600 21.984
13.846
ppm

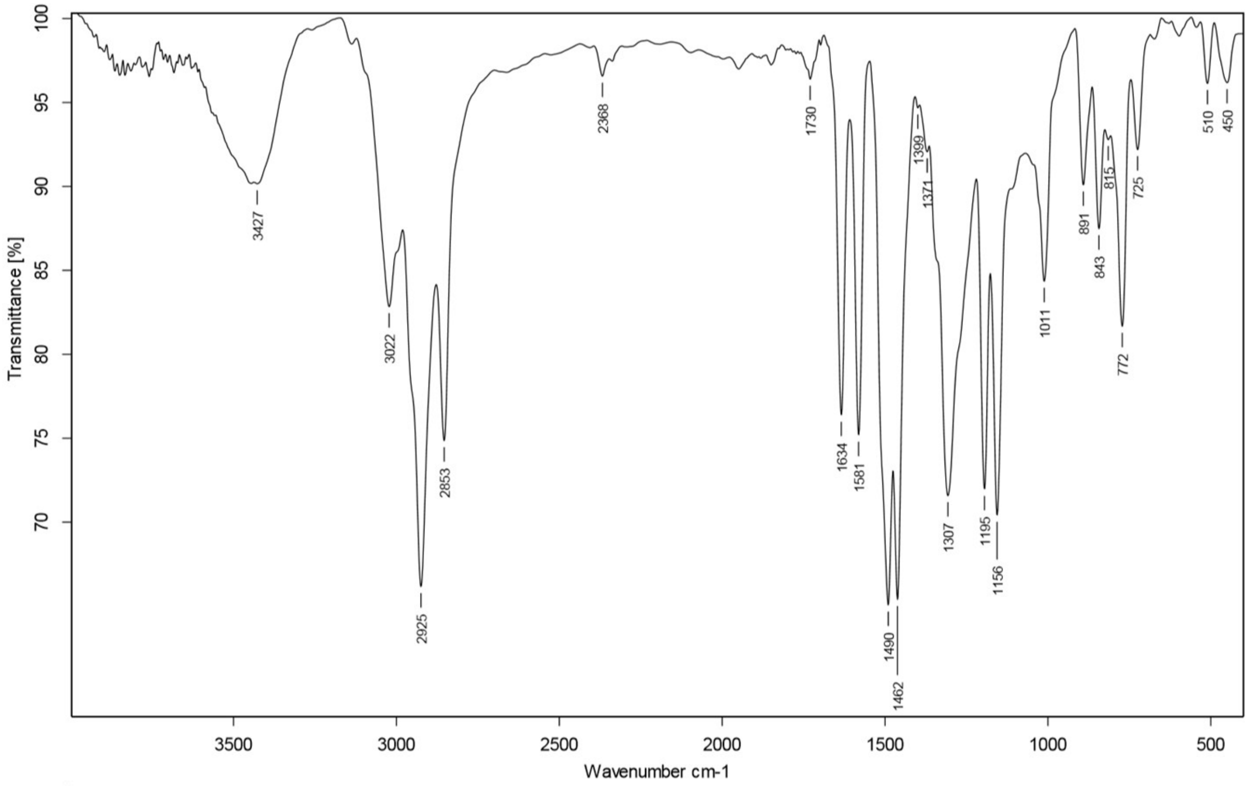

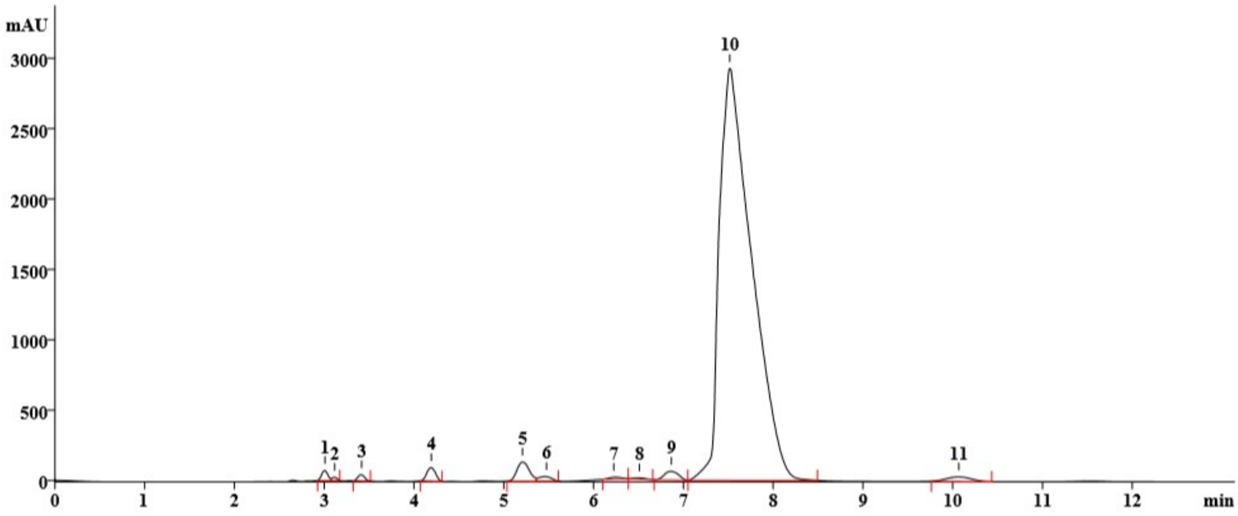

Peak table

Peak	*Retention min*	*Area*	*Height*	*Width h/2*	*Area%*	*Type*
1	3,01	343,248	70,771	0,0783	0,443	BD :
2	3,12	92,594	22,236	0,0757	0,119	DB :
3	3,41	228,850	42,901	0,0860	0,295	BB :
4	4,19	648,035	94,876	0,112	0,836	BB :
5	5,21	1299,790	135,258	0,153	1,677	BD :
6	5,48	314,818	30,833	0,181	0,406	DB :
7	6,23	114,119	12,382	0,155	0,147	BB :
8	6,51	76,834	8,378	0,158	0,0991	BB :
9	6,86	672,870	61,565	0,181	0,868	BB :
10	7,52	73090,901	2925,584	0,401	94,320	BB :
11	10,07	610,738	31,747	0,315	0,788	BB :

7, 2BP4BO-12, Br

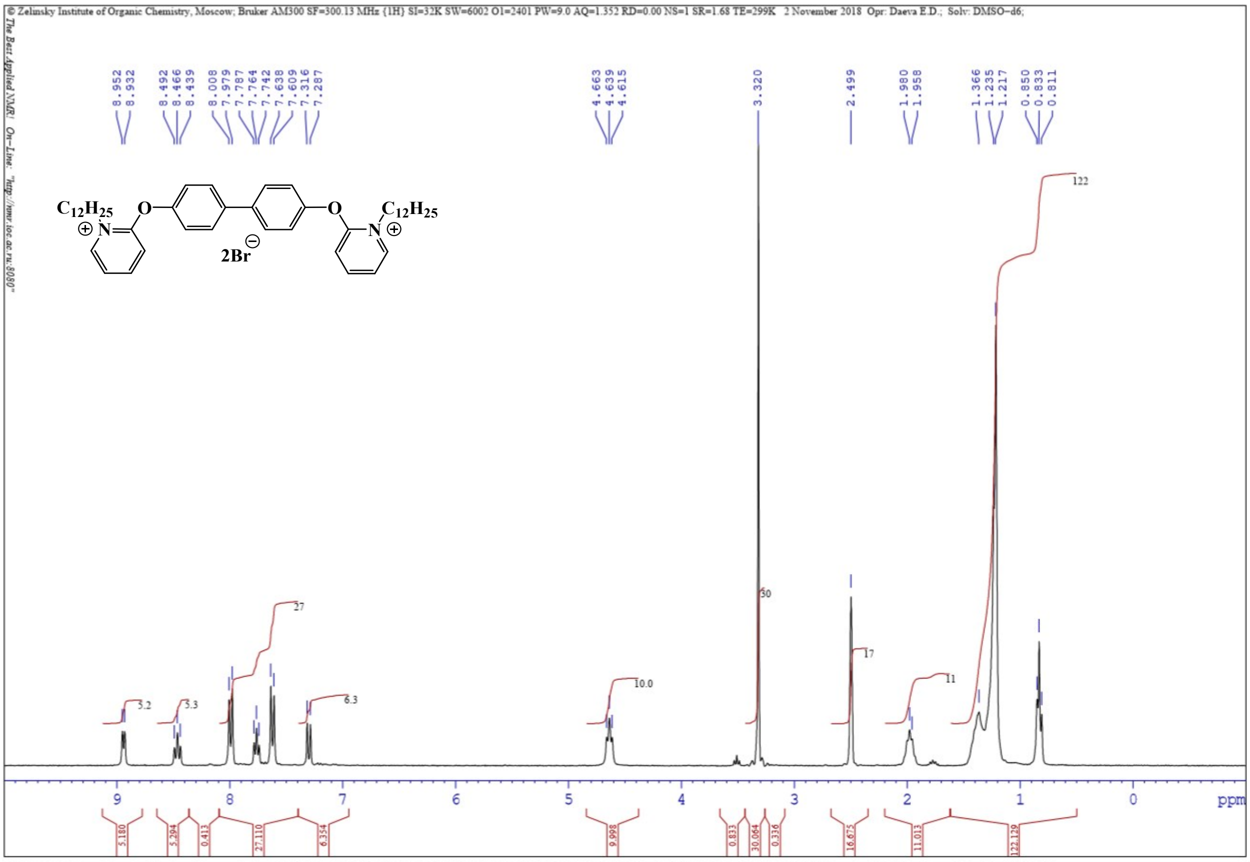

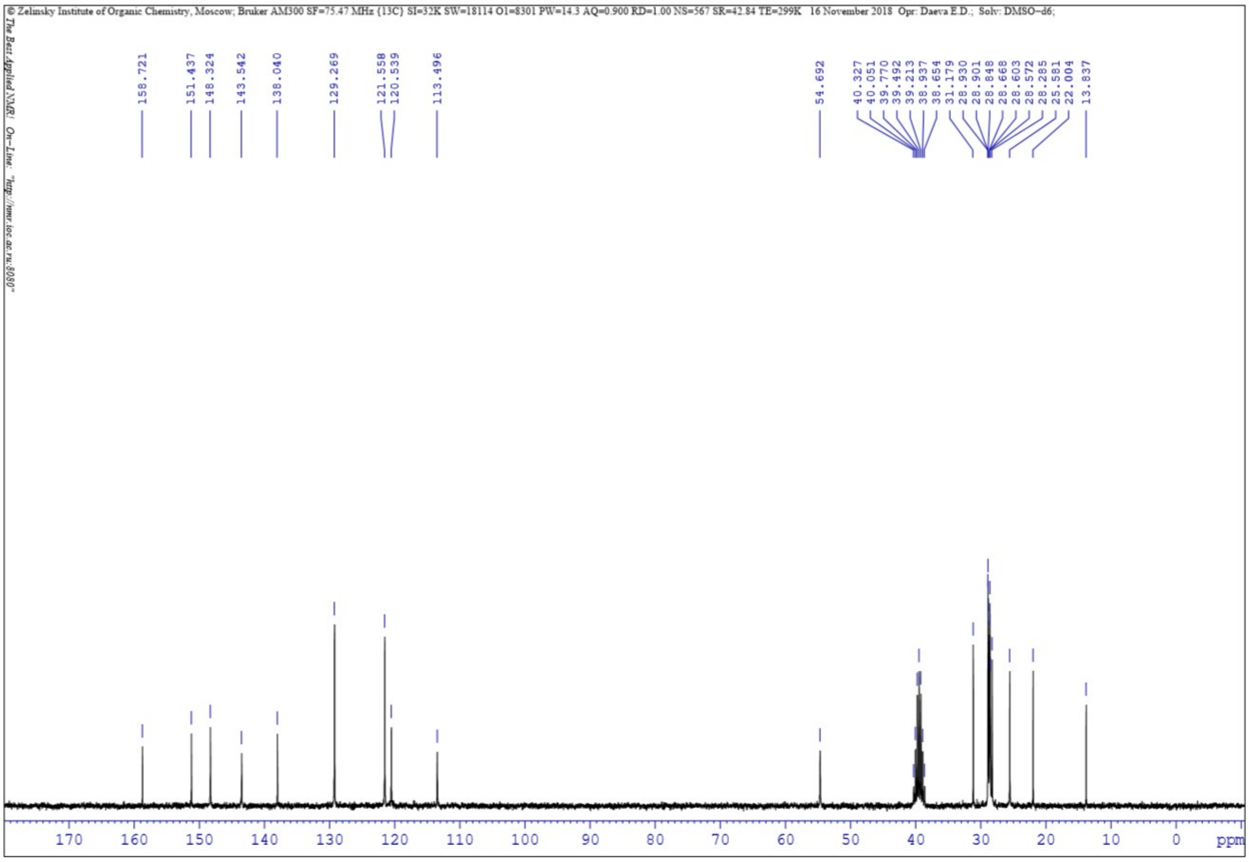

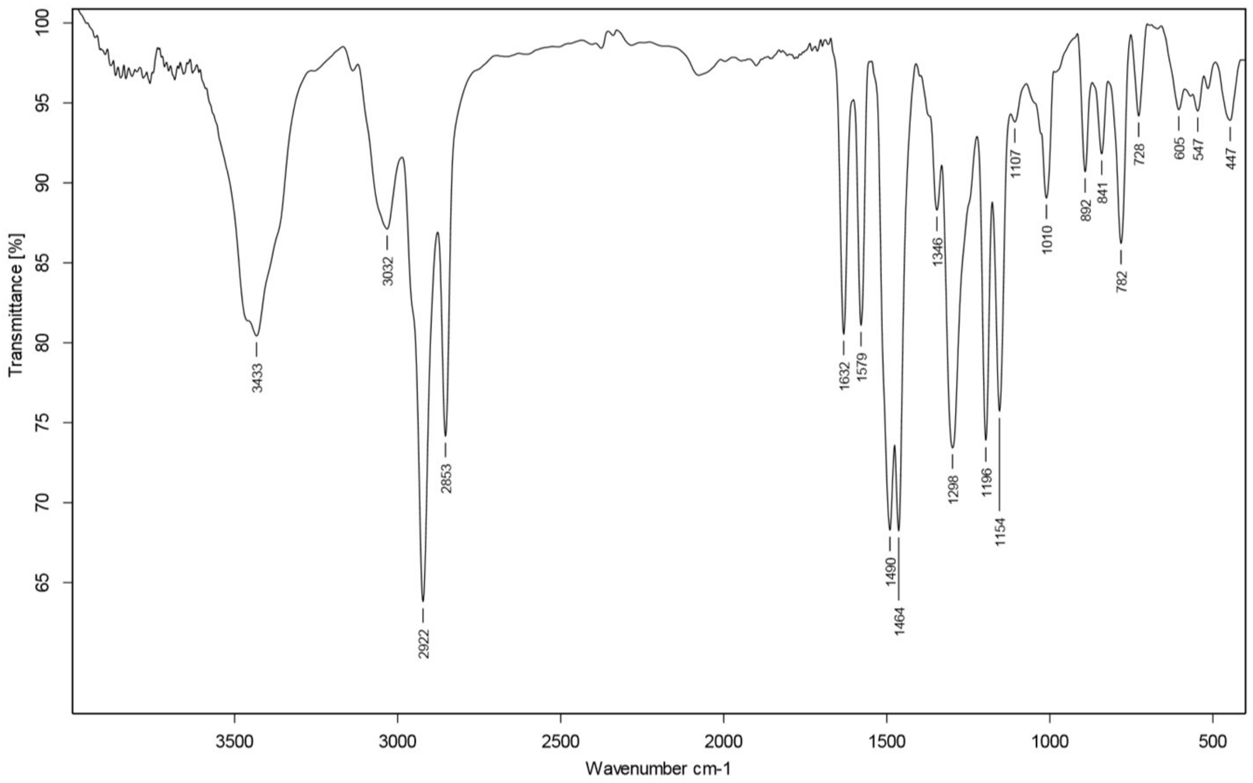

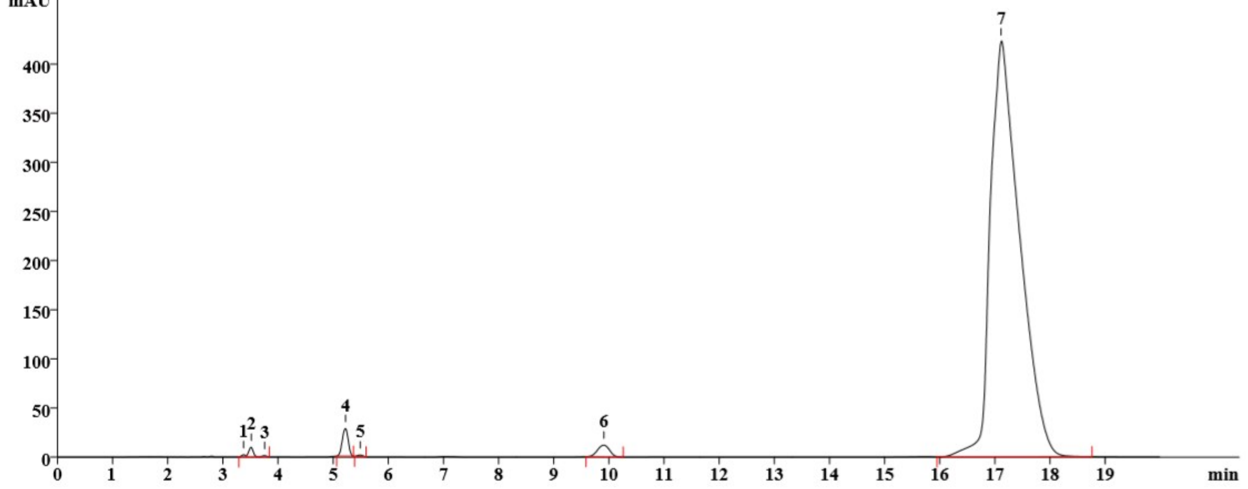

Peak table

Peak	Retention min	Area	Height	Width h/2	Area%	Type
1	3,38	10,884	2,093	0,0948	0,0677	BD :
2	3,51	55,463	9,714	0,0911	0,345	DD :
3	3,75	7,843	1,244	0,0942	0,0488	DB :
4	5,22	209,805	27,943	0,121	1,305	BB :
5	5,49	9,669	1,412	0,115	0,0602	BB :
6	9,91	180,969	11,854	0,244	1,126	BB :
7	17,12	15599,212	423,054	0,585	97,047	BB :

8, 2BP4BO-12, I

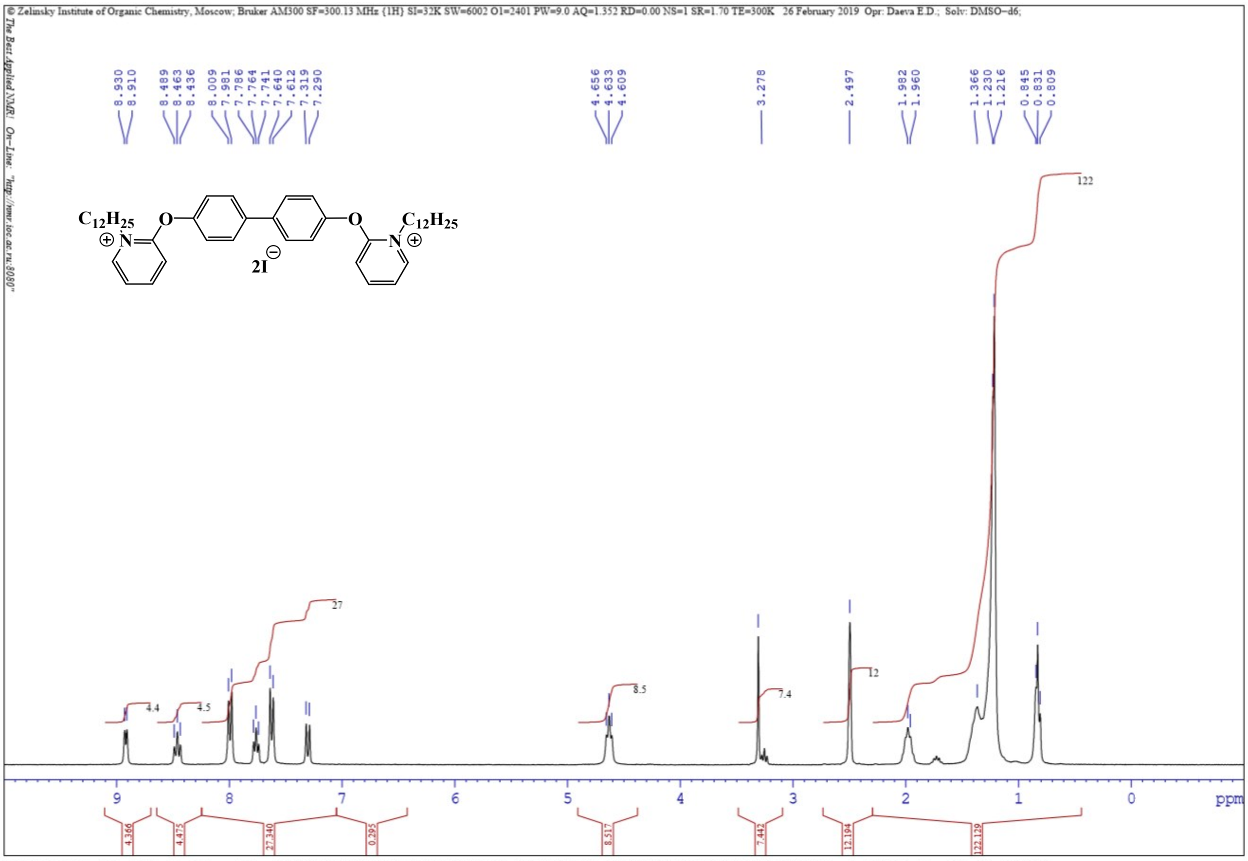
© Zelinsky Institute of Organic Chemistry, Moscow; Bruker AM300 SF=300.13 MHz {1H} SI=32K SW=6002 O1=2401 PW=9.0 AQ=1.352 RD=0.00 NS=1 SR=1.70 TE=300K 26 February 2019 Opr: Daeva E.D.; Solv: DMSO-d6;
8.930 8.910 8.489 8.463 8.436 8.009 7.981 7.786 7.764 7.741 7.640 7.612 7.319 7.290 4.656 4.633 4.609 3.278 2.497 1.982 1.960 1.366 1.230 1.216 0.845 0.831 0.809
ppm

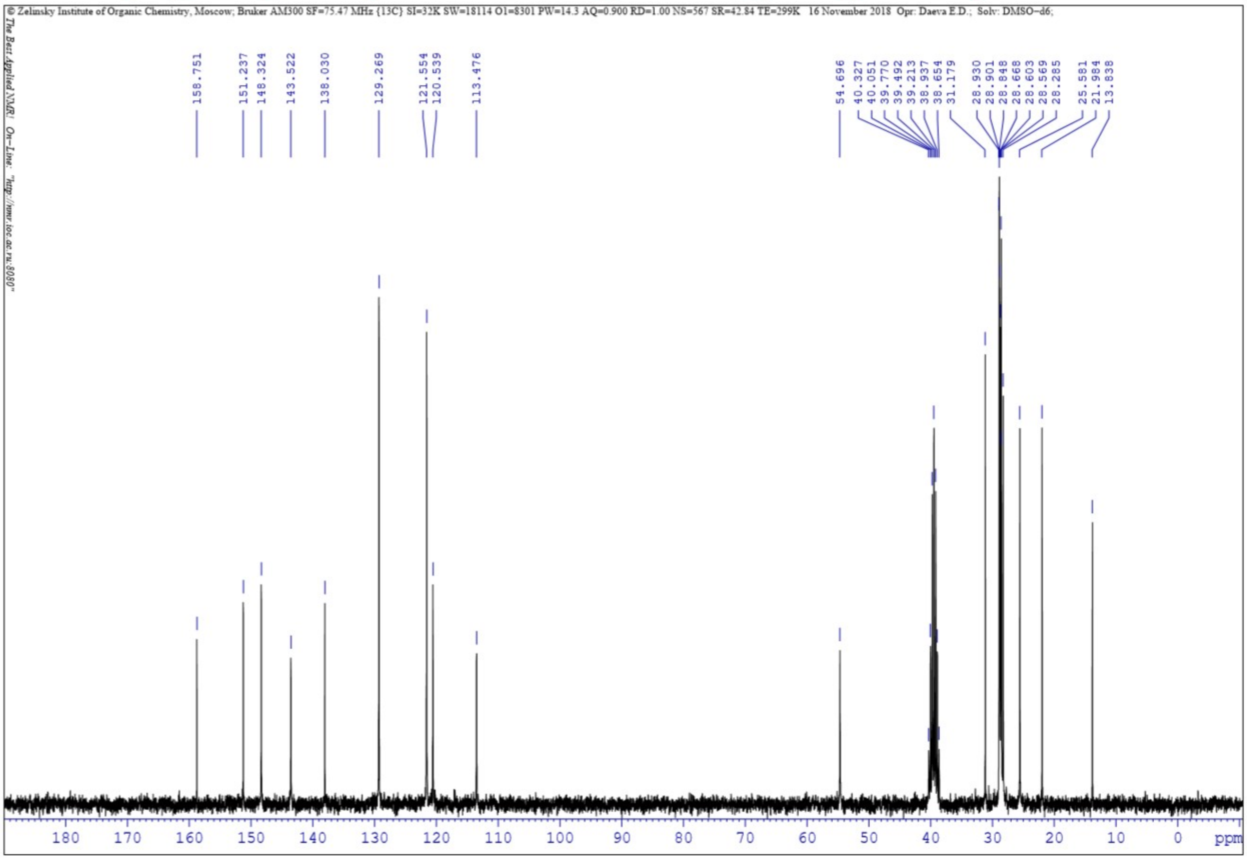
© Zelinsky Institute of Organic Chemistry, Moscow; Bruker AM300 SF=75.47 MHz {13C} SI=32K SW=18114 O1=8301 PW=14.3 AQ=0.900 RD=1.00 NS=567 SR=42.84 TE=299K 16 November 2018 Opr: Daeva E.D.; Solv: DMSO-d6;
158.751 151.237 148.324 143.522 138.030 129.269 121.554 120.539 113.476
54.696 40.327 40.051 39.770 39.492 39.213 38.937 38.654 31.179 28.930 28.901 28.848 28.668 28.603 28.569 28.285 25.581 21.984 13.838
ppm

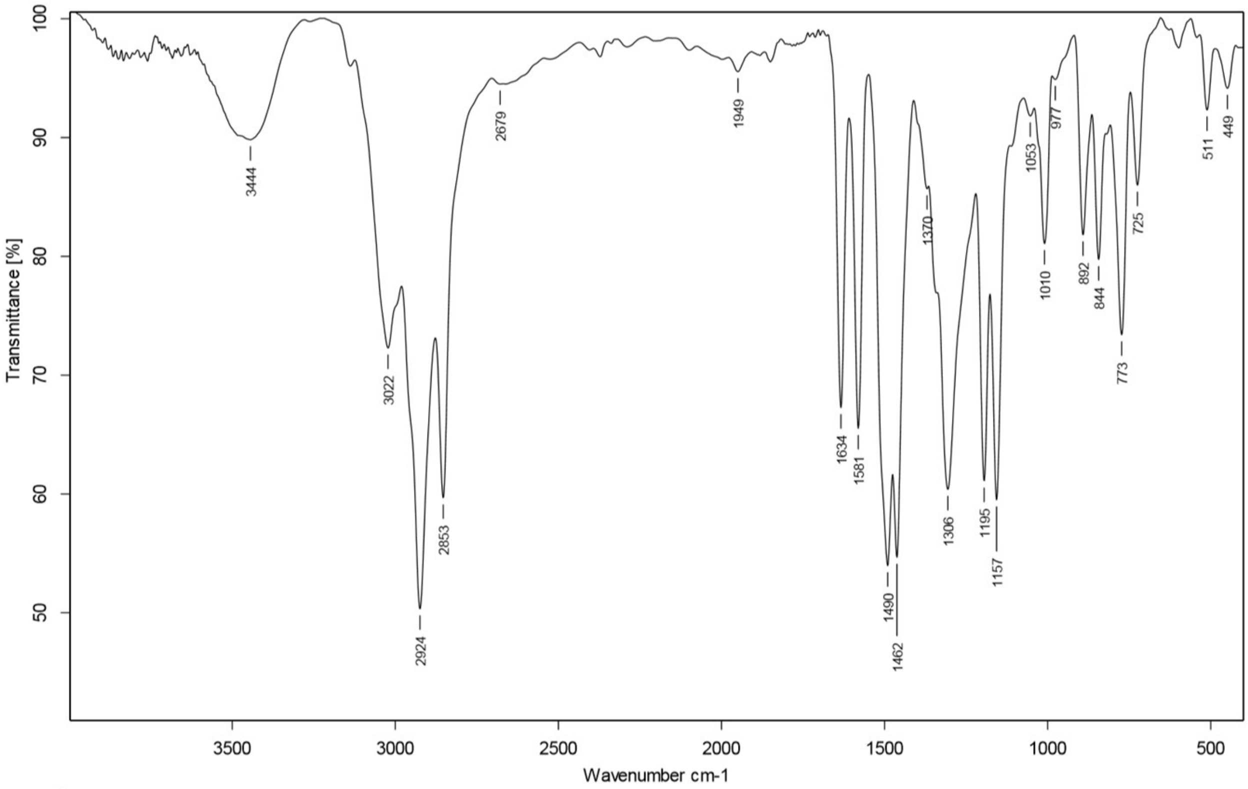

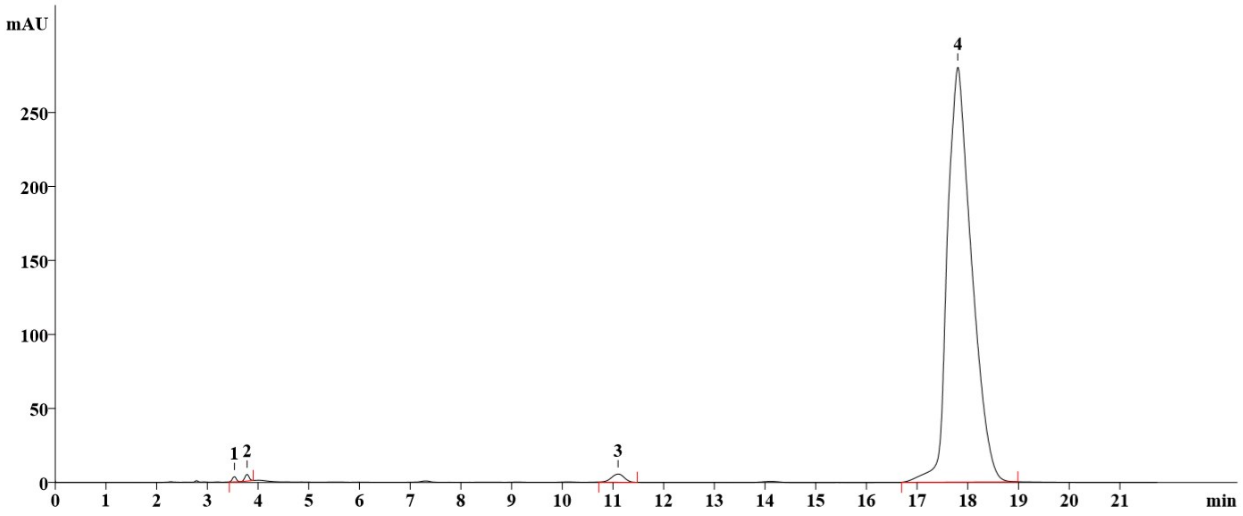

Peak table

Peak	Retention min	Area	Height	Width h/2	Area%	Type
1	3,53	18,673	3,433	0,0861	0,198	BD :
2	3,78	26,446	4,443	0,0936	0,280	DB :
3	11,11	95,752	5,472	0,277	1,013	BB :
4	17,80	9307,291	280,258	0,522	98,509	BB :

10, 4BP4BO-8, Br

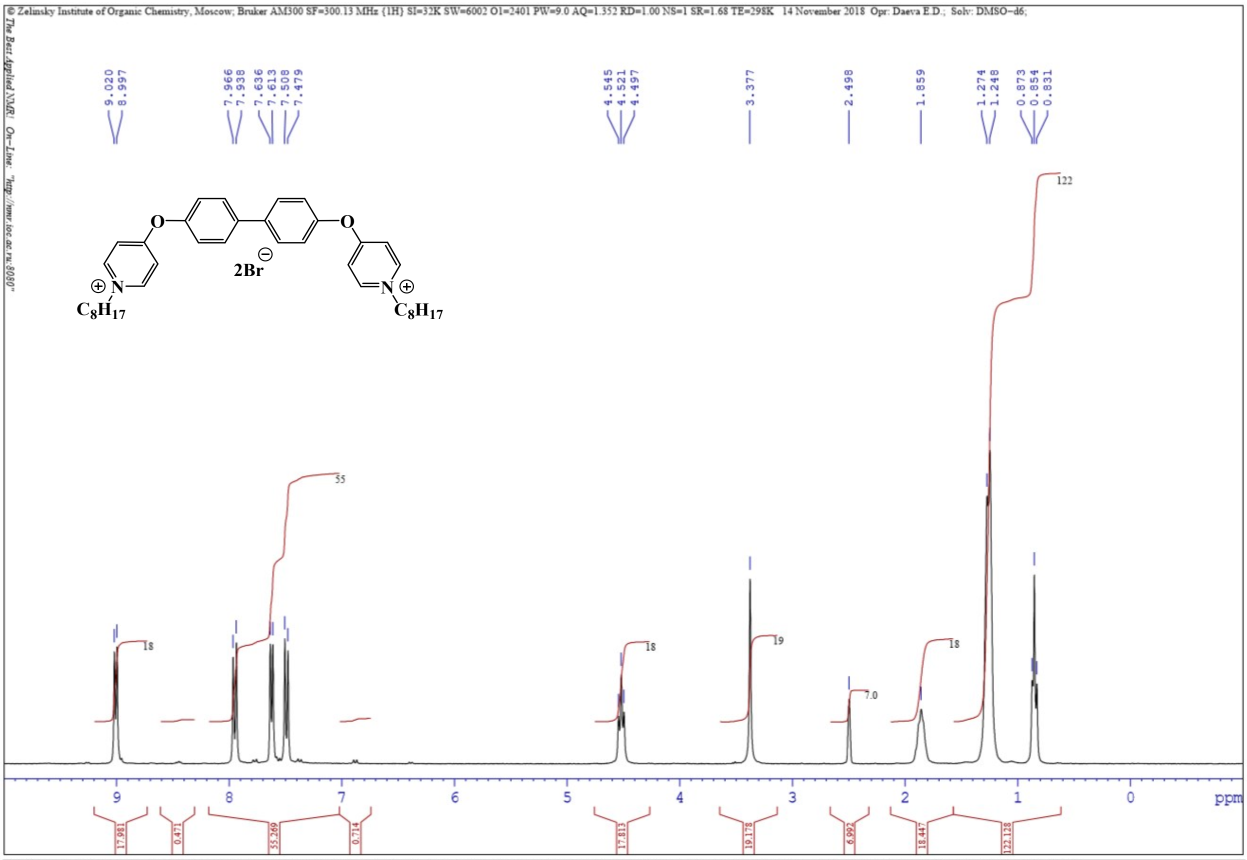

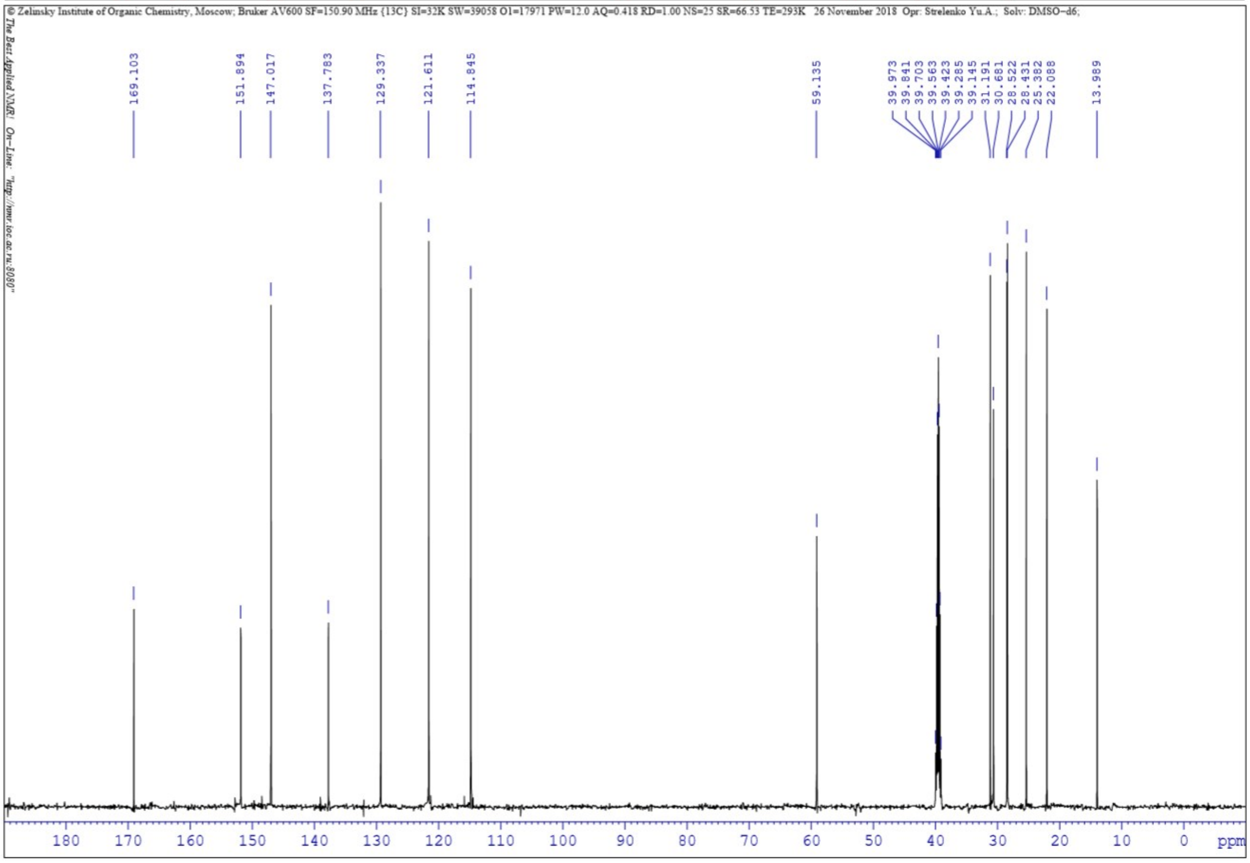

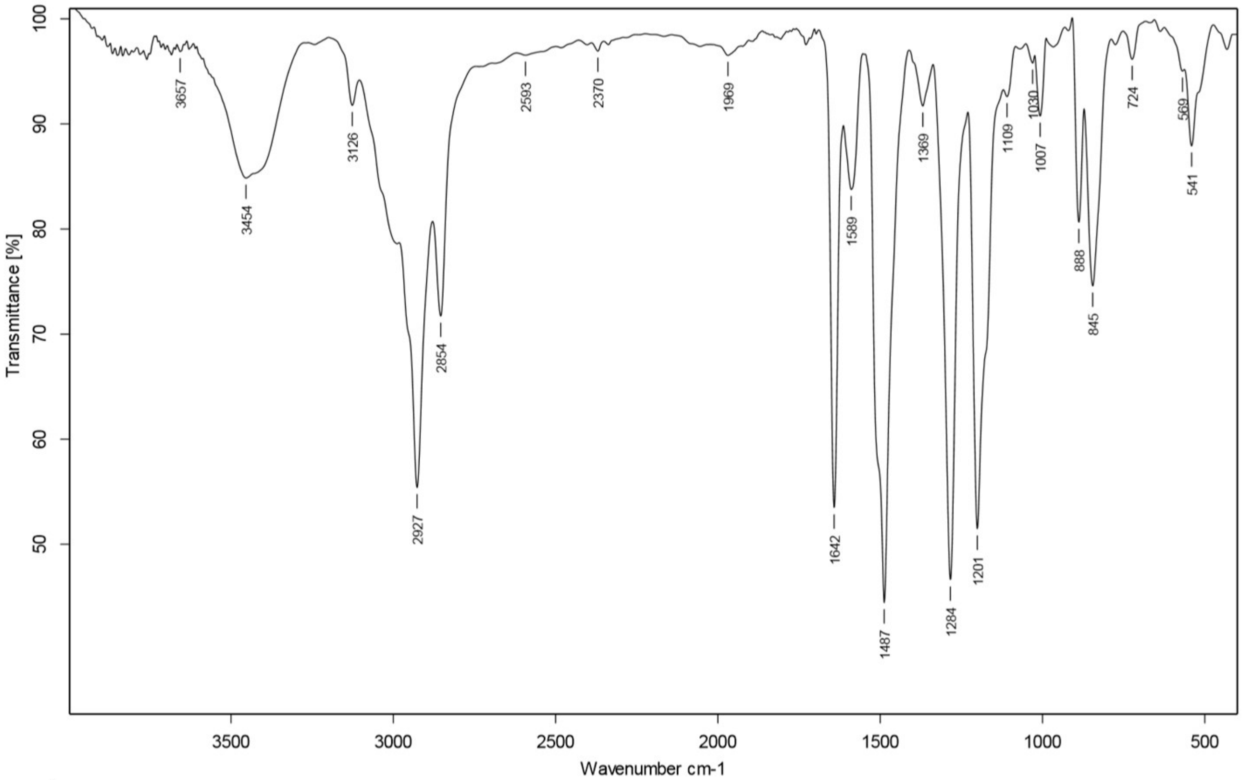

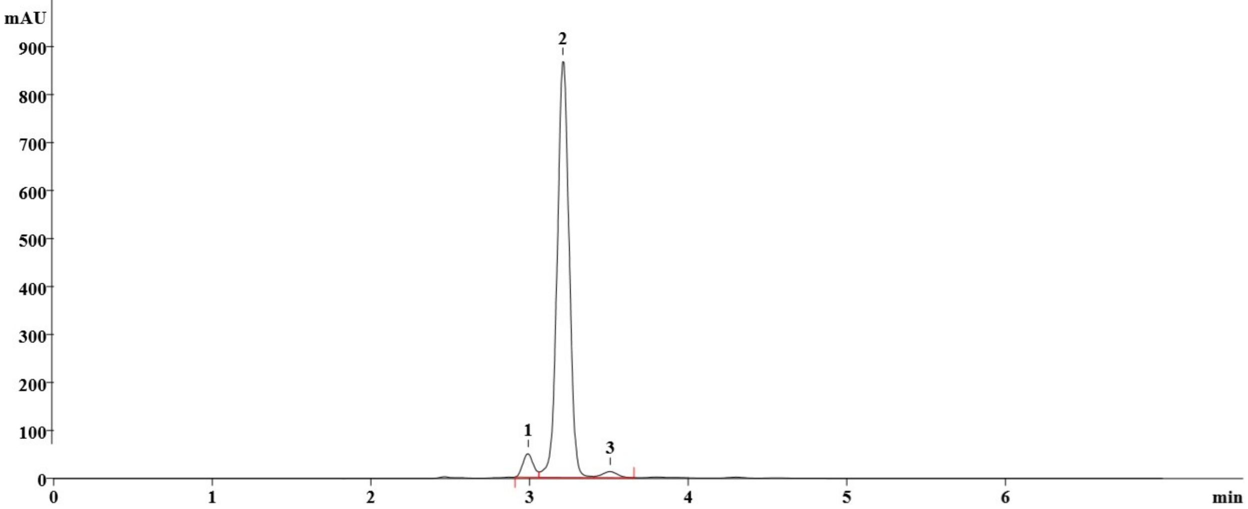

Peak table

Peak	Retention min	Area	Height	Width h/2	Area%	Type
1	2,99	223,788	48,496	0,0741	4,616	BD :
2	3,21	4540,737	867,161	0,0807	93,669	DD :
3	3,51	83,128	12,270	0,105	1,715	DB :

11, 4BP4BO-8, I

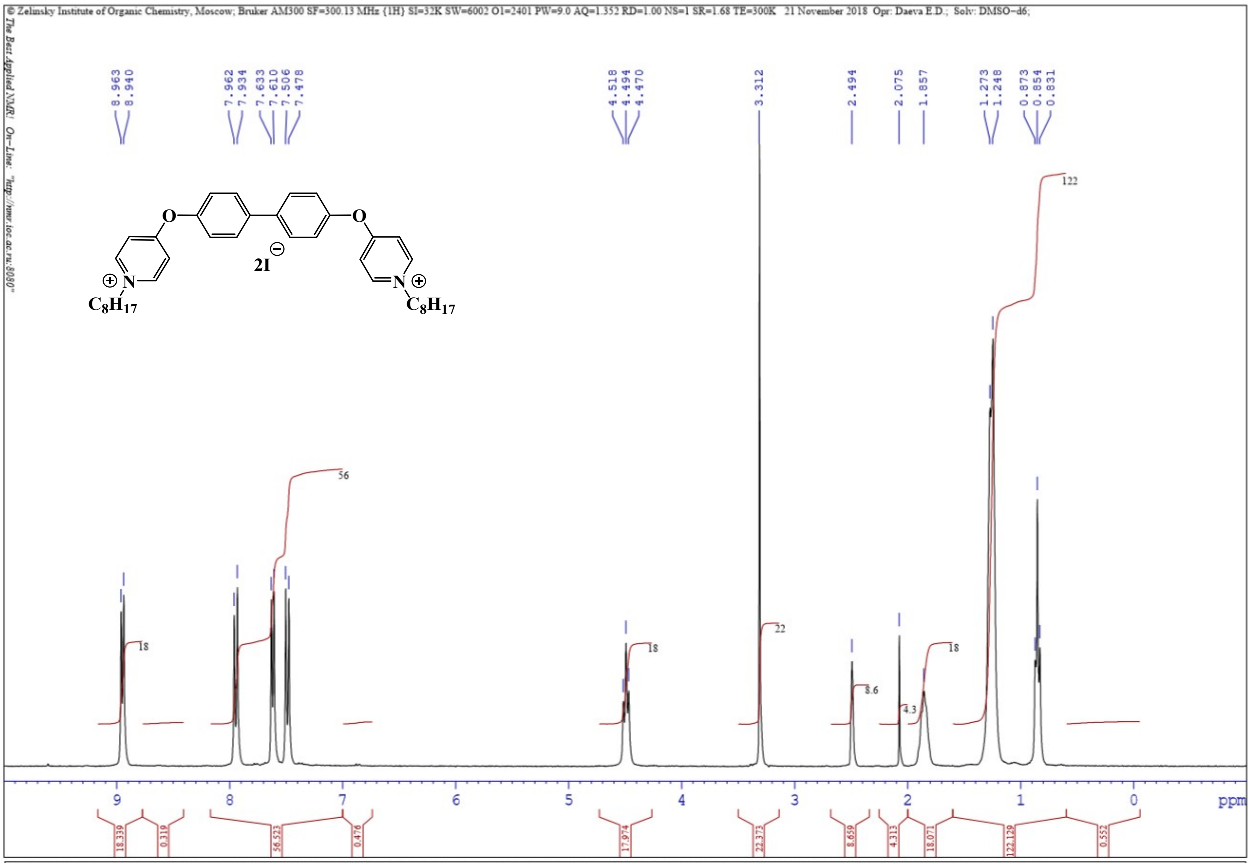
© Zelinsky Institute of Organic Chemistry, Moscow; Bruker AM300 SF=300.13 MHz {1H} SI=32K SW=6002 O1=2401 PW=9.0 AQ=1.352 RD=1.00 NS=1 SR=1.68 TE=300K 21 November 2018 Opr: Daeva E.D.; Solv: DMSO−d6;
The Best Applied NMR! On−Line: "http://nmr.ioc.ac.ru:8080"
8.963
8.940
7.962
7.934
7.633
7.610
7.506
7.478
4.518
4.494
4.470
3.312
2.494
2.075
1.857
1.273
1.248
0.873
0.854
0.831
O
O
2I
N
N
C8H17
C8H17
122
56
18
18
22
8.6
4.3
18
ppm
18.339
0.319
56.523
0.476
17.974
22.373
8.659
4.313
18.071
122.129
0.552

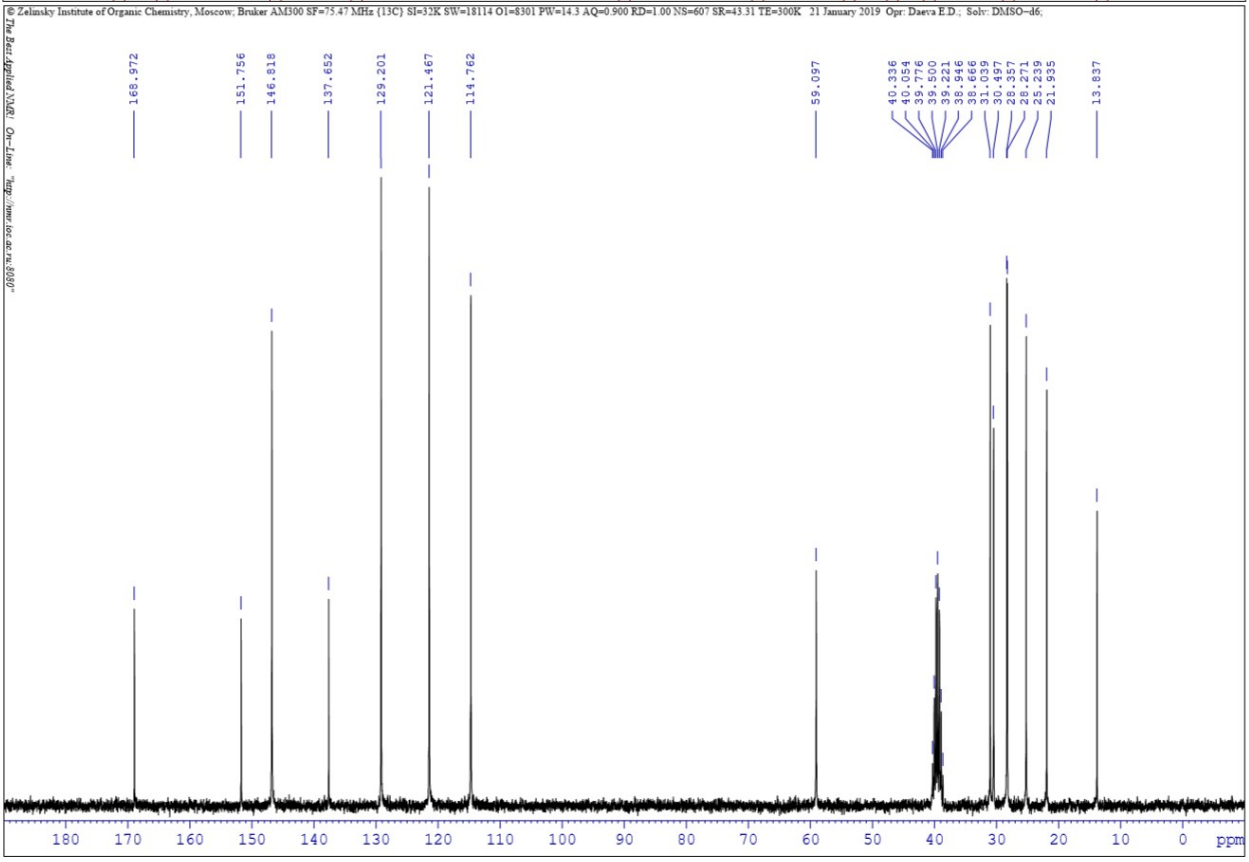
© Zelinsky Institute of Organic Chemistry, Moscow; Bruker AM300 SF=75.47 MHz {13C} SI=32K SW=18114 O1=8301 PW=14.3 AQ=0.900 RD=1.00 NS=607 SR=43.31 TE=300K 21 January 2019 Opr: Daeva E.D.; Solv: DMSO−d6;
The Best Applied NMR! On−Line: "http://nmr.ioc.ac.ru:8080"
168.972
151.756
146.818
137.652
129.201
121.467
114.762
59.097
40.336
40.054
39.776
39.500
39.221
38.946
38.666
31.039
30.497
28.357
28.271
25.239
21.935
13.837
ppm

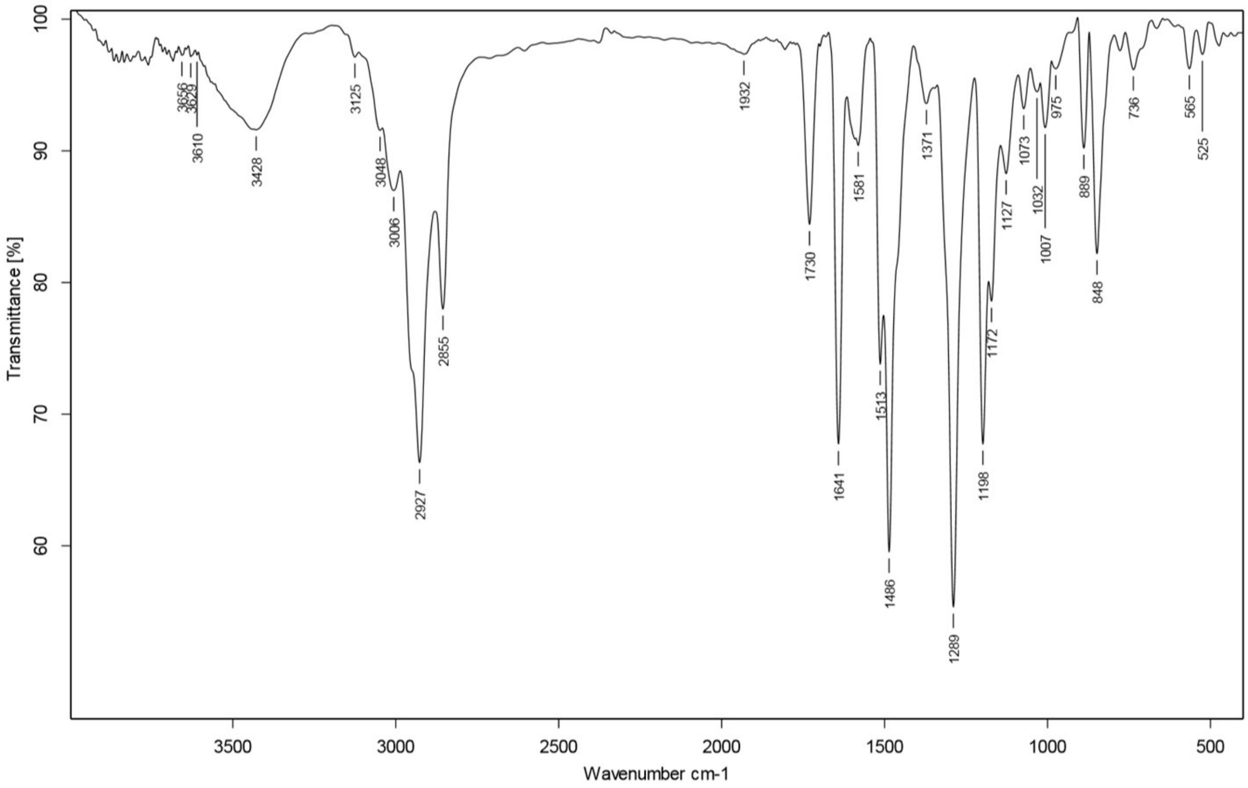

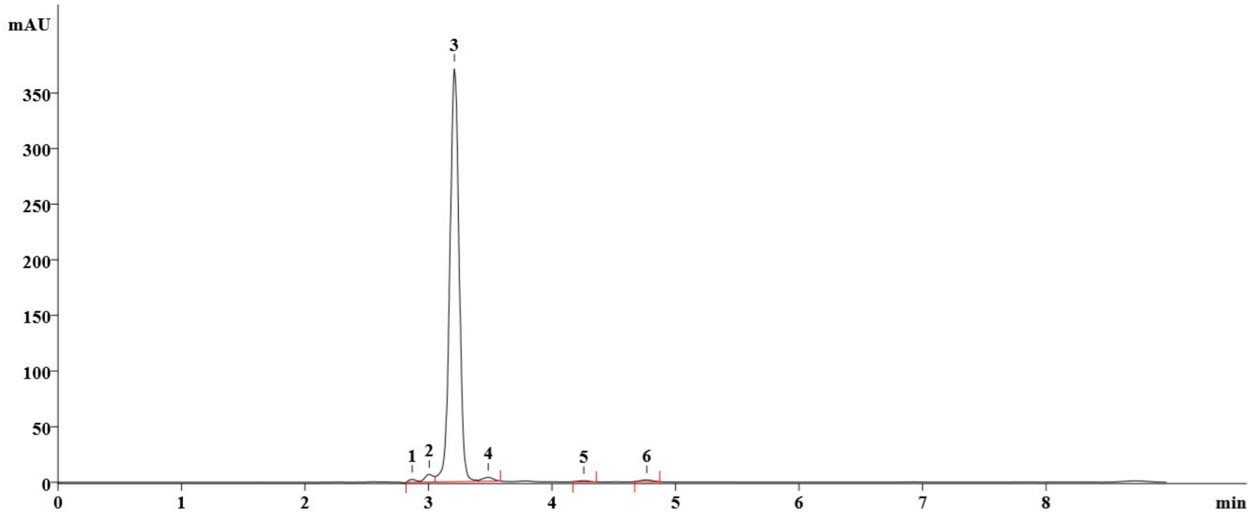

Peak table

Peak	Retention min	Area	Height	Width h/2	Area%	Type
1	2,87	10,964	2,608	0,0688	0,542	BD :
2	3,01	32,860	6,752	0,0851	1,626	DD :
3	3,21	1939,650	370,315	0,0797	95,969	DD :
4	3,48	21,433	3,409	0,105	1,060	DB :
5	4,26	5,962	0,983	0,100	0,295	BB :
6	4,77	10,260	1,554	0,111	0,508	BB :

12, 4BP4BO-10, Br

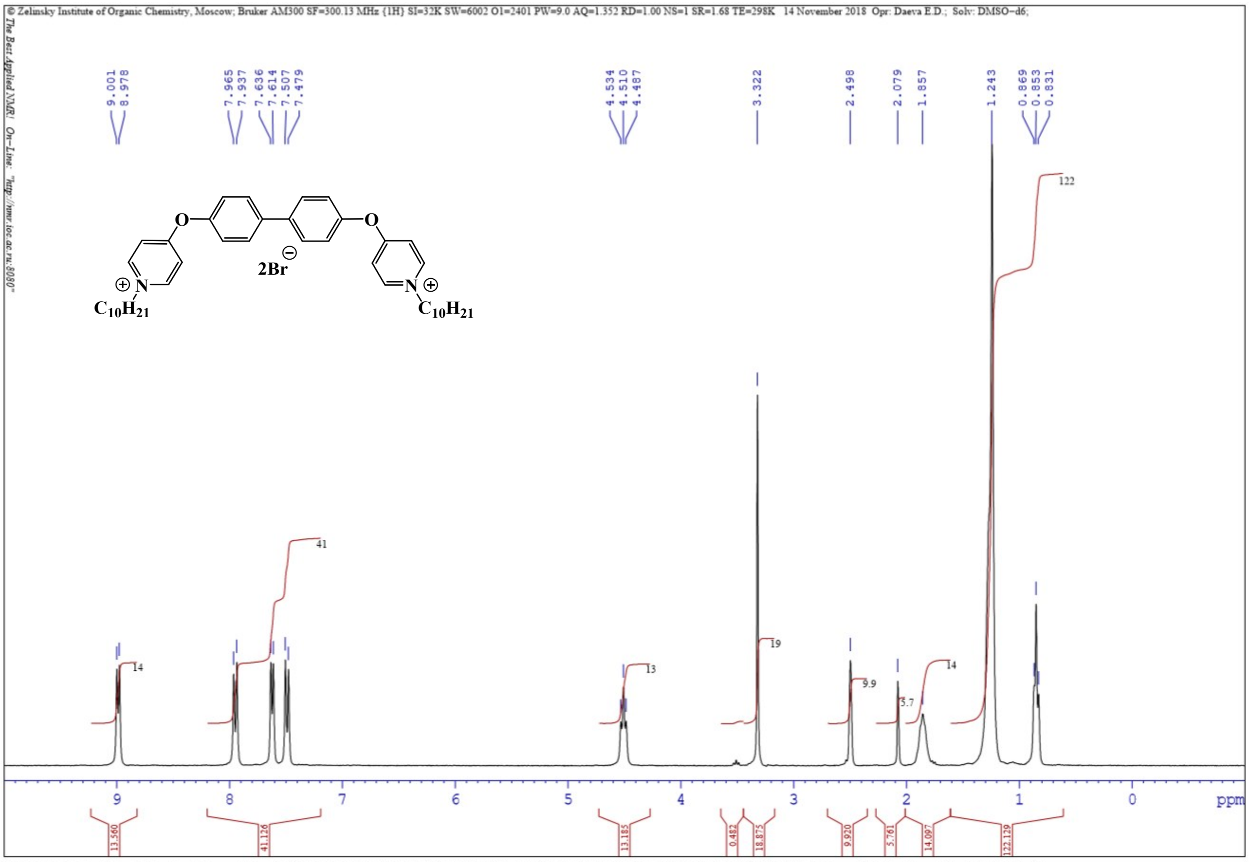

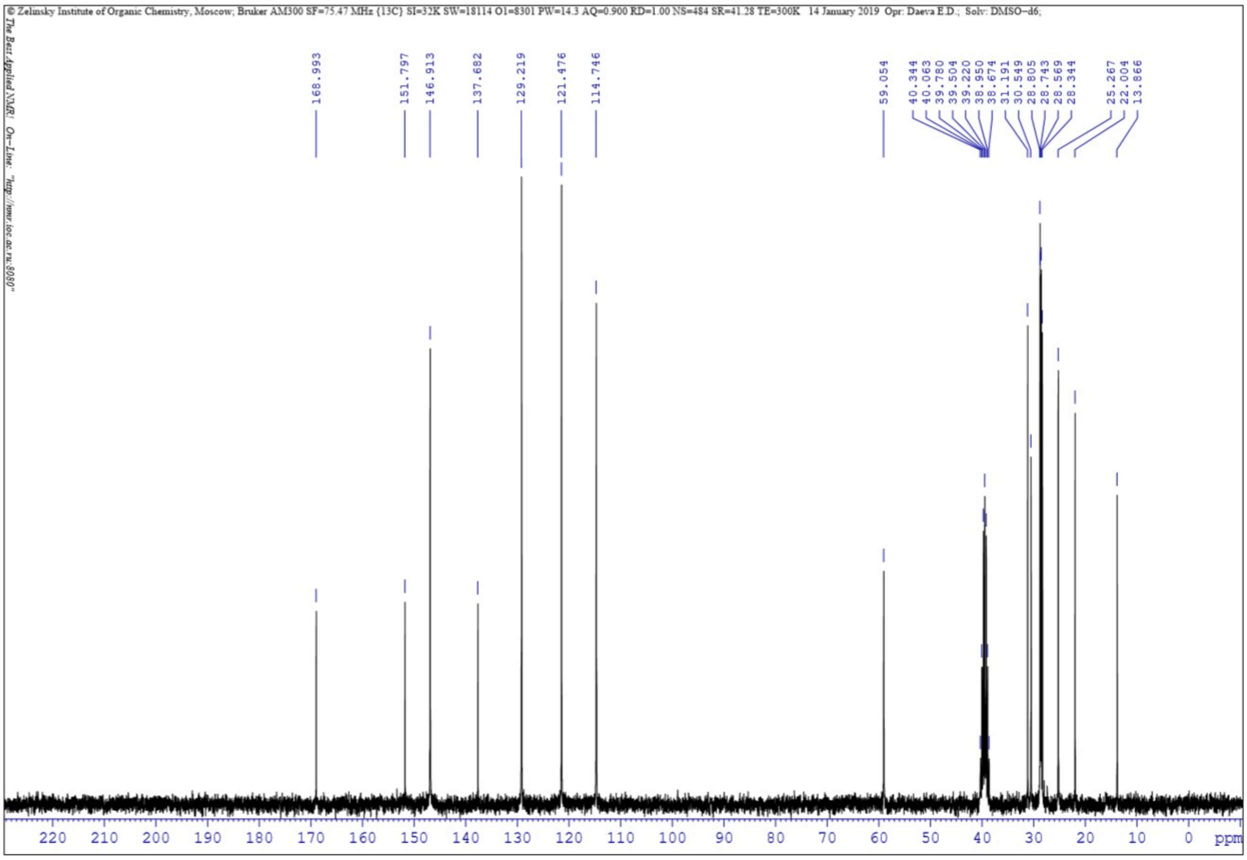

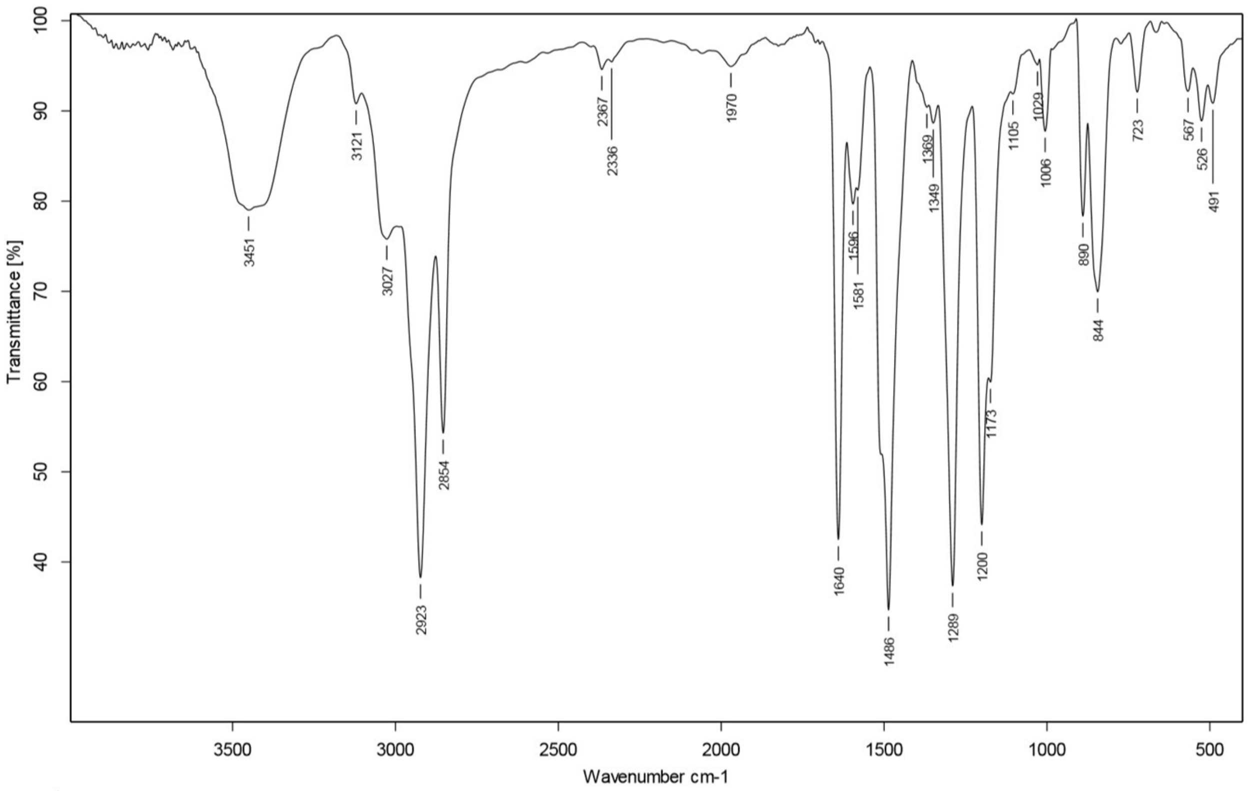

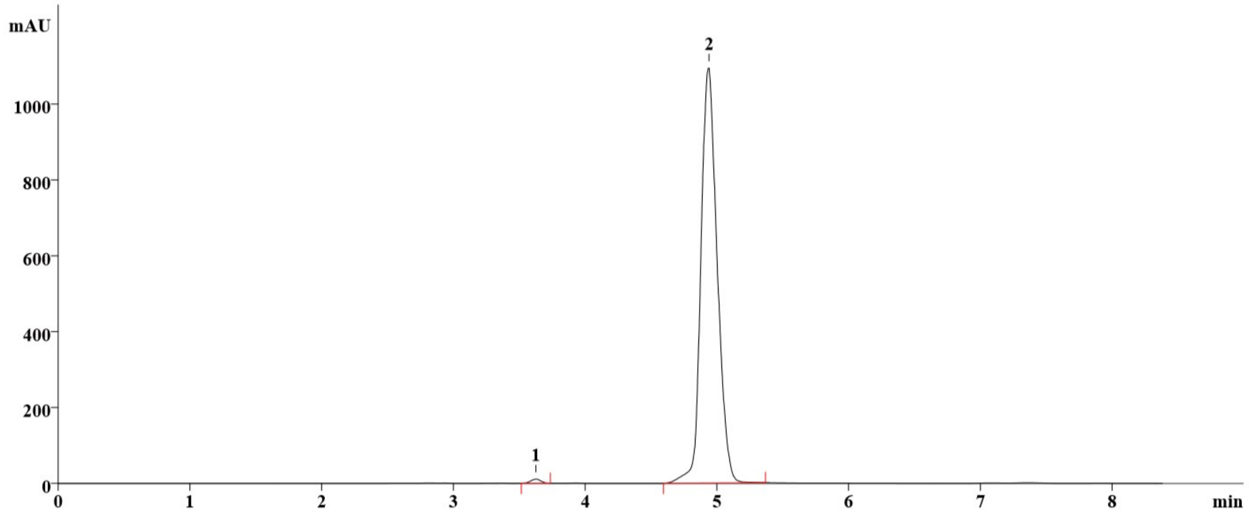

Peak table

Peak	Retention min	Area	Height	Width h/2	Area%	Type
1	3,63	58,408	10,867	0,0851	0,618	BB :
2	4,94	9397,859	1094,477	0,131	99,382	BB :

13, 4BP4BO-10, I

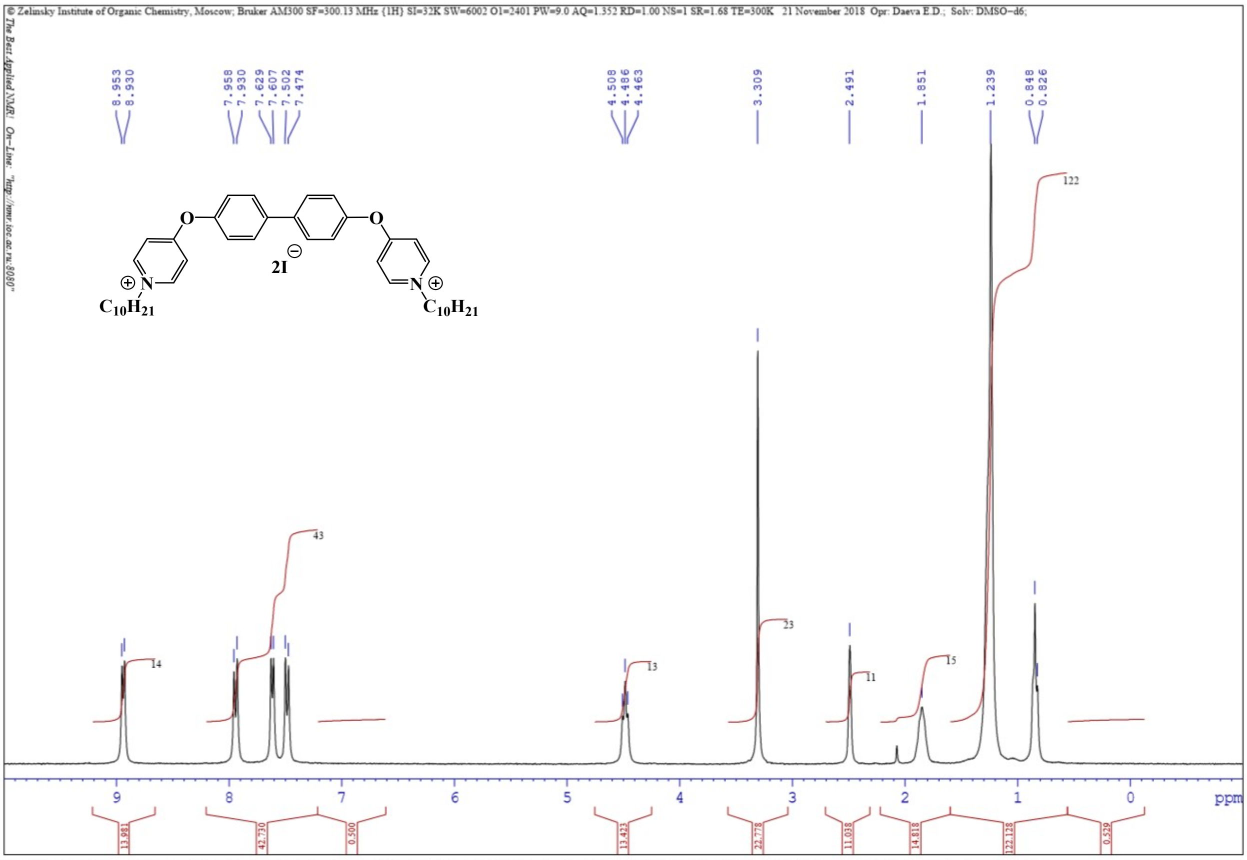

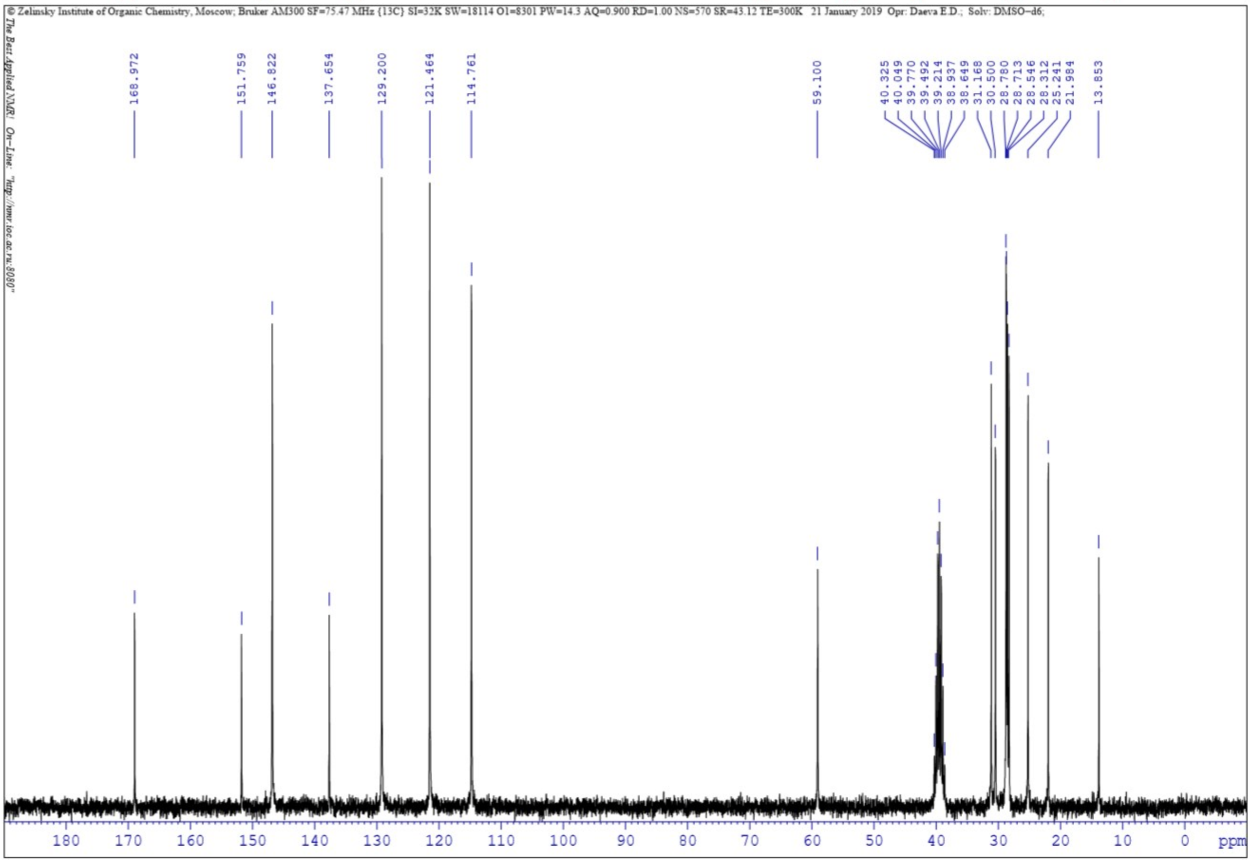

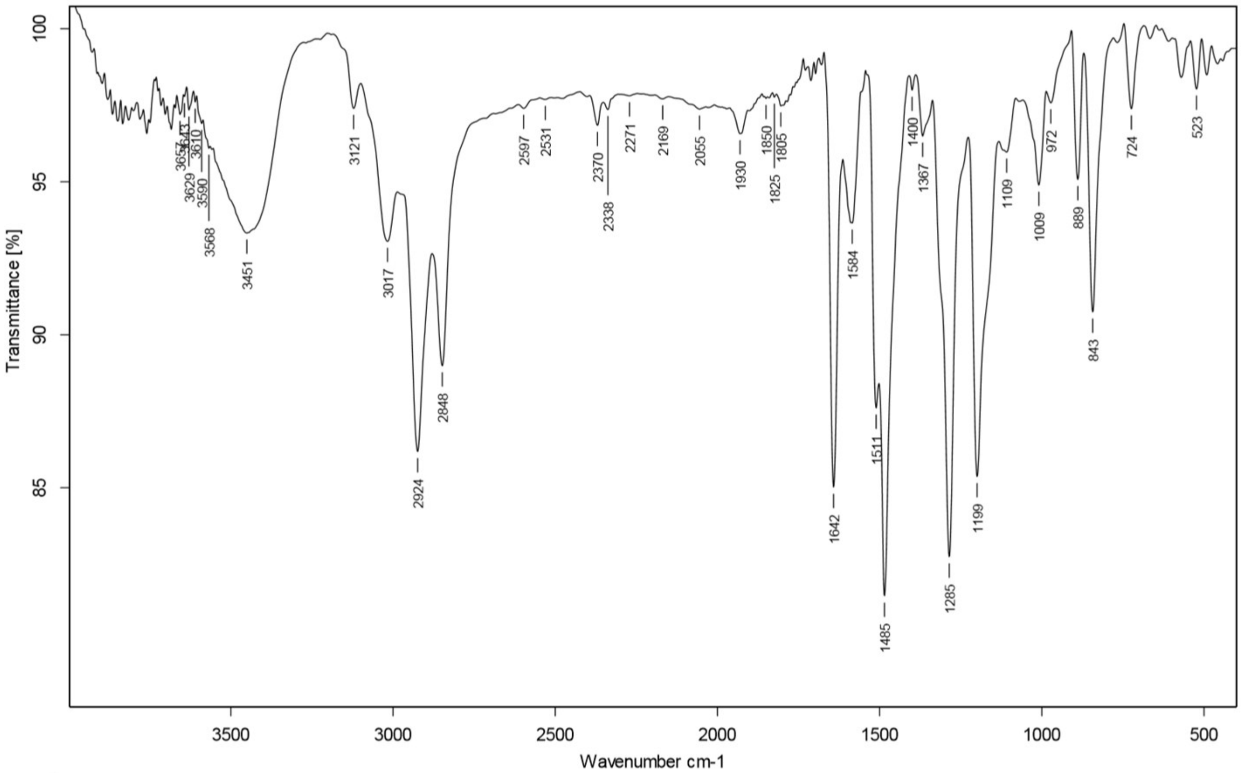

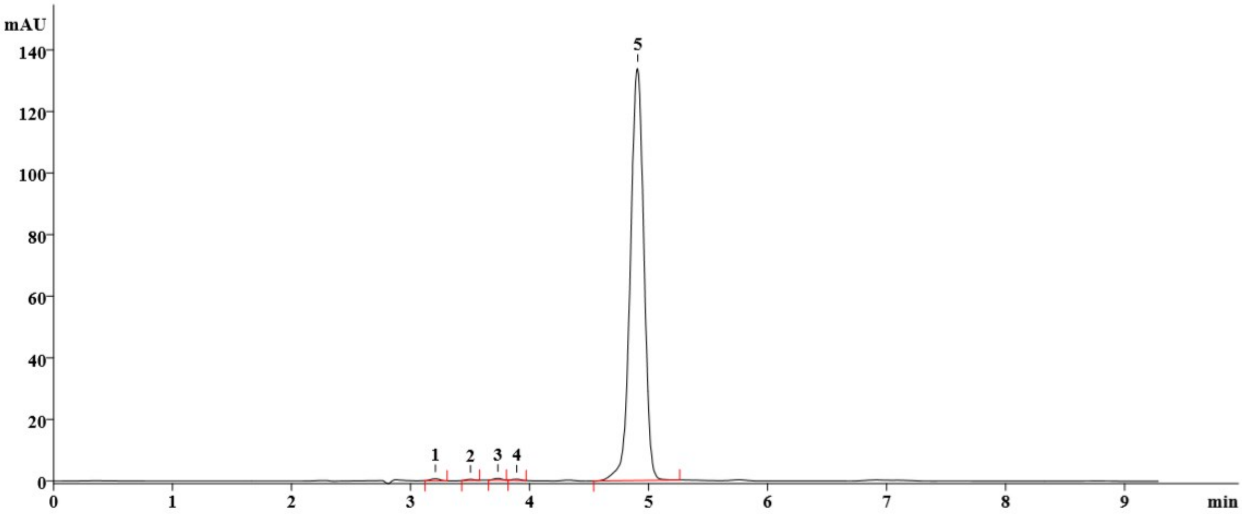

Peak table

Peak	Retention min	Area	Height	Width h/2	Area%	Type
1	3,21	3,264	0,627	0,0829	0,304	BB :
2	3,50	1,587	0,325	0,0801	0,148	BB :
3	3,73	2,581	0,538	0,0792	0,241	BB :
4	3,90	1,805	0,371	0,0810	0,168	BB :
5	4,91	1063,513	133,756	0,121	99,139	BB :

14, 4BP4BO-12, Br

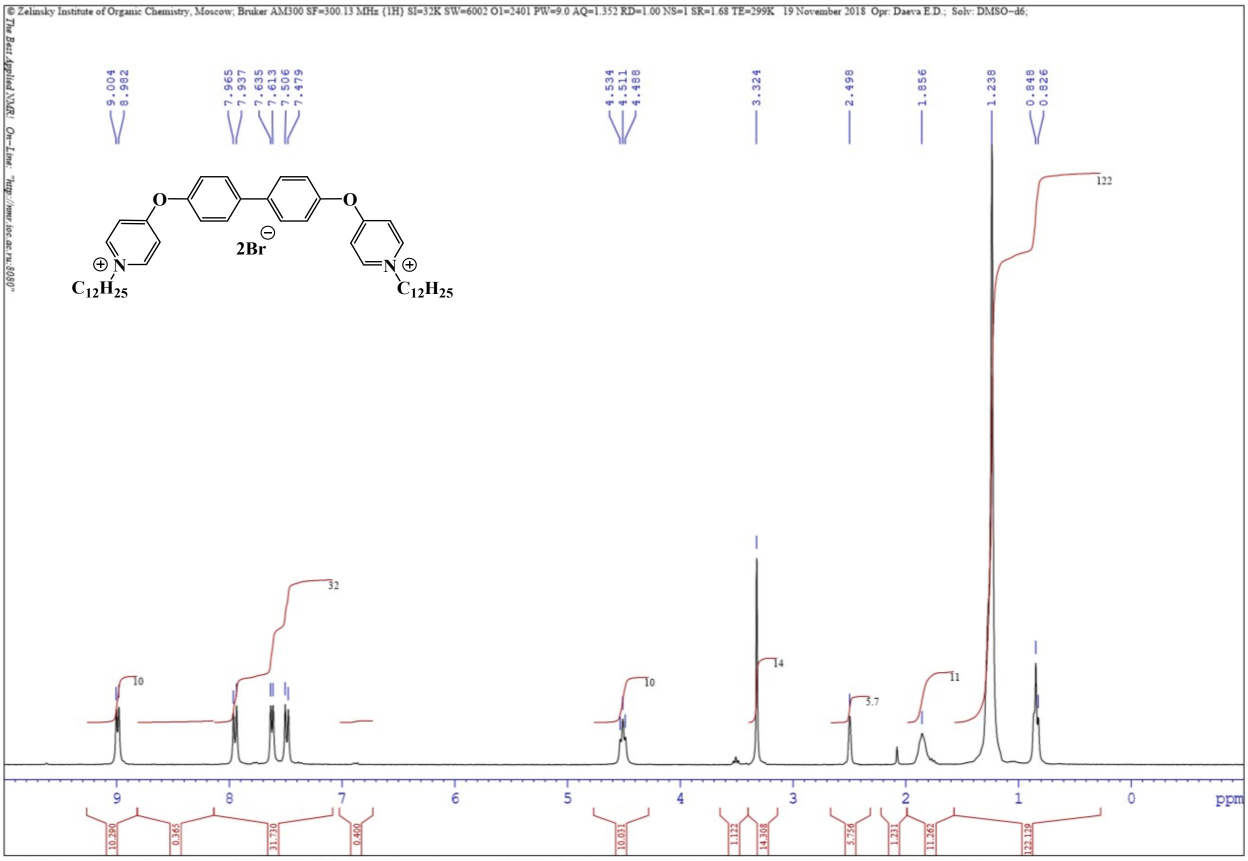

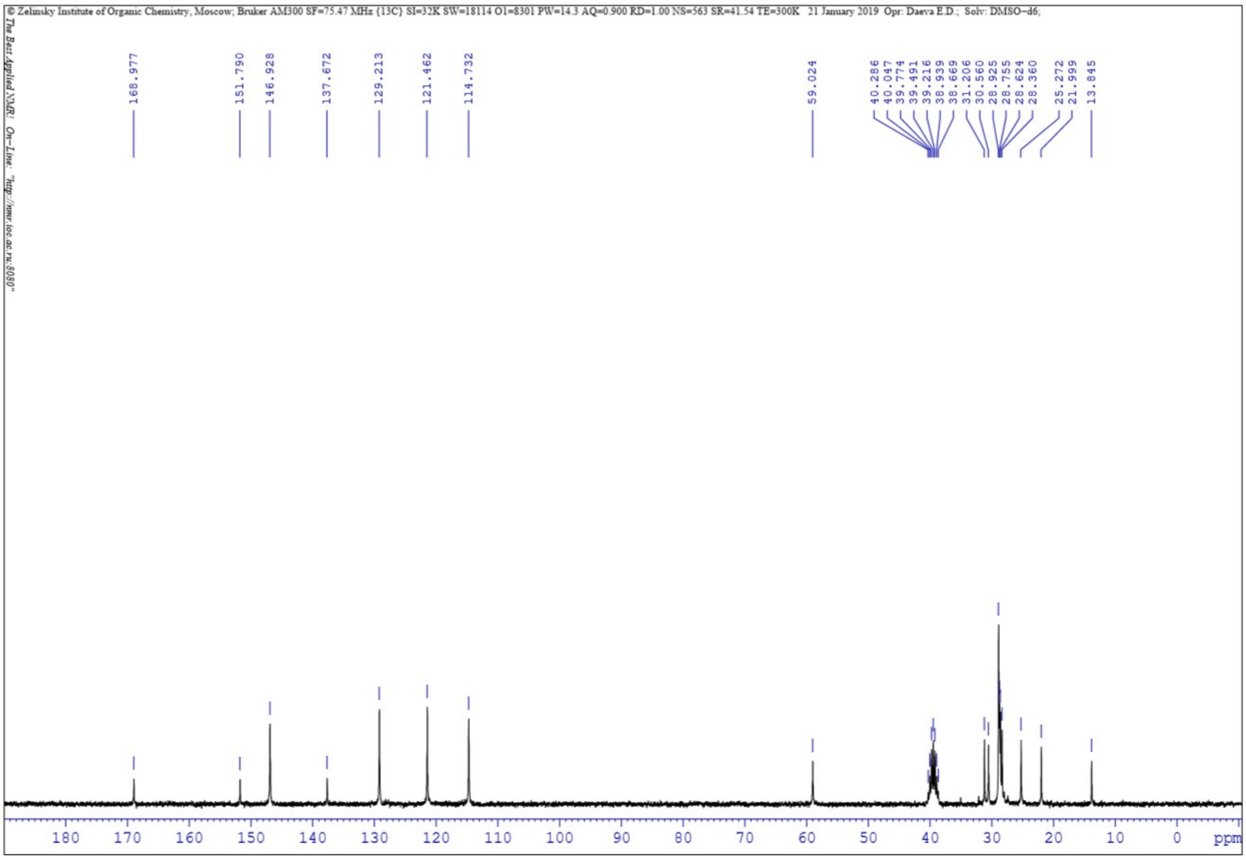

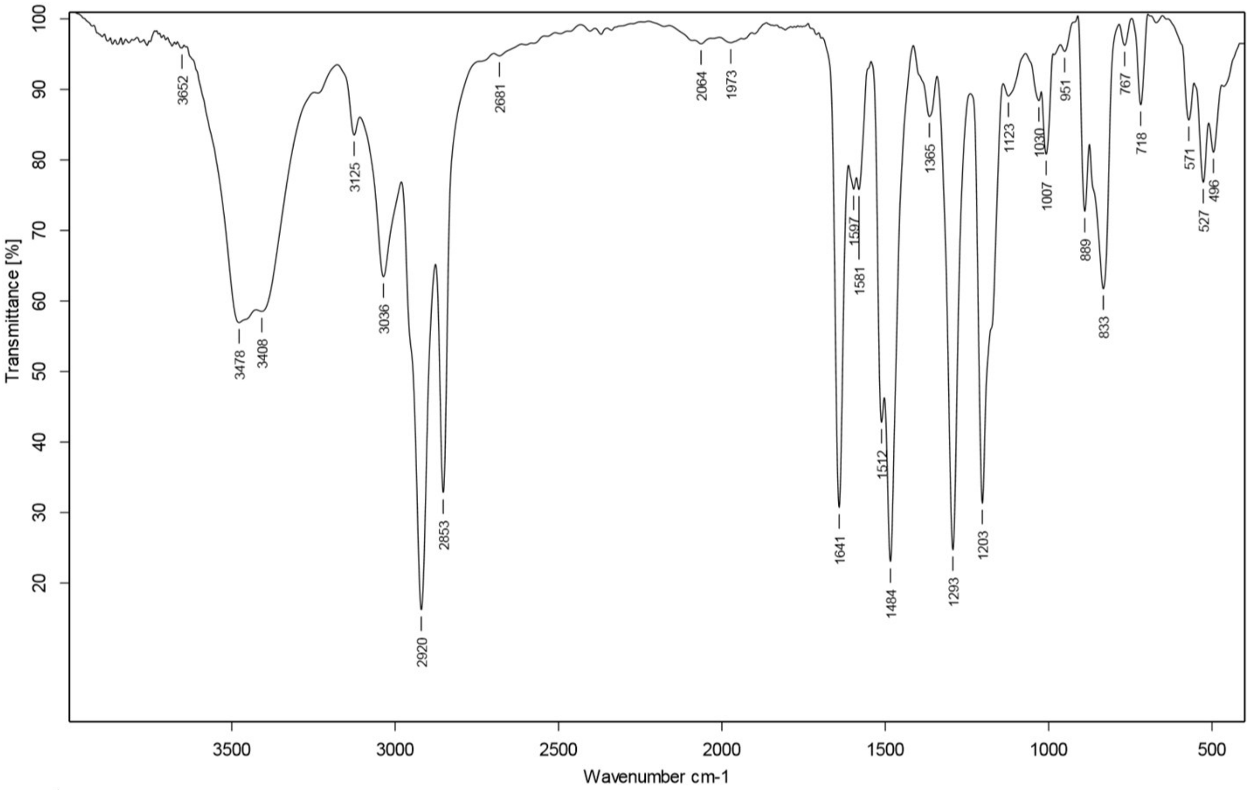

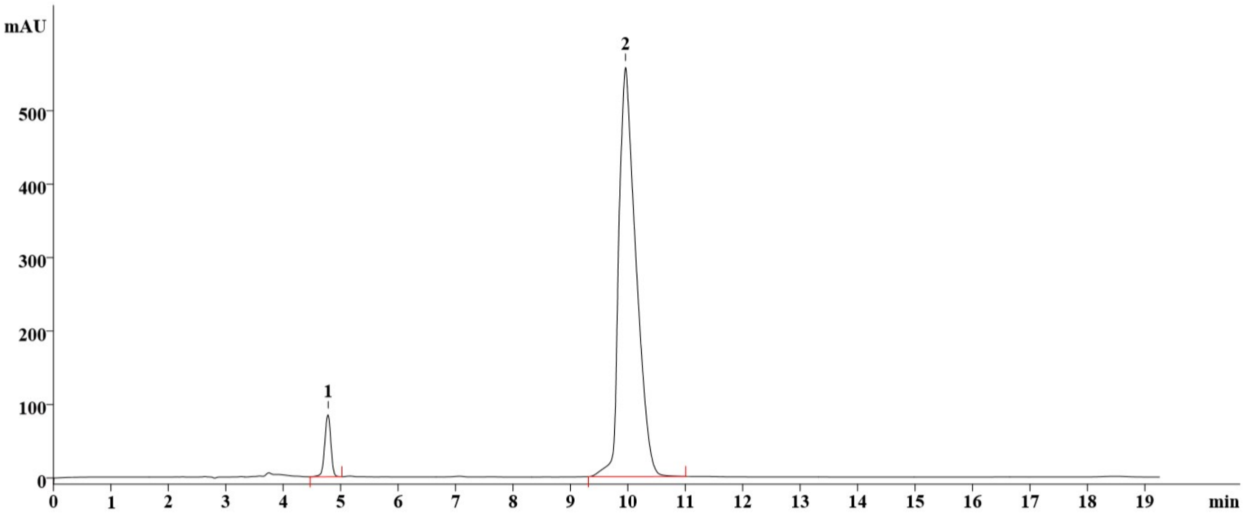

Peak table

Peak	Retention min	Area	Height	Width h/2	Area%	Type
1	4,78	626,415	84,113	0,114	5,151	BB :
2	9,96	11535,600	556,792	0,325	94,849	BB :

15, 4BP4BO-12, I

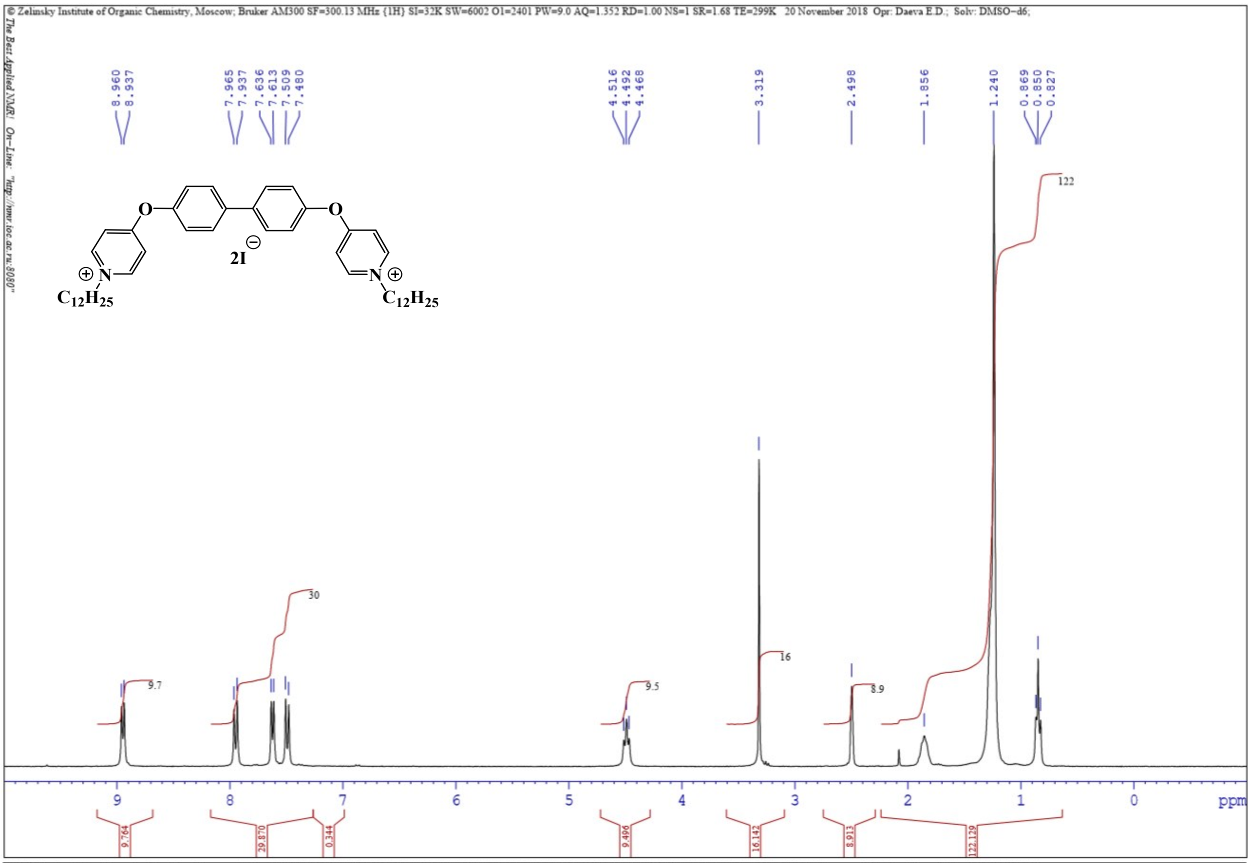

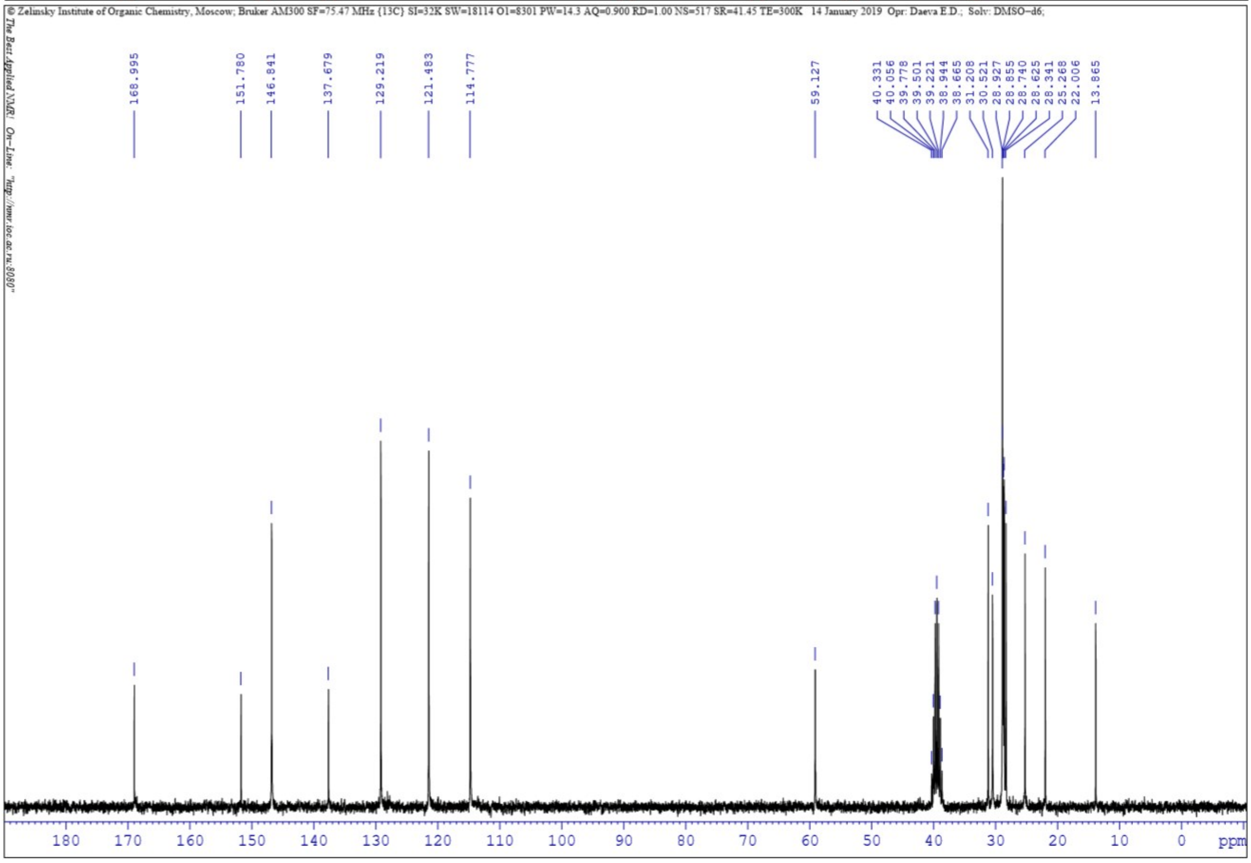

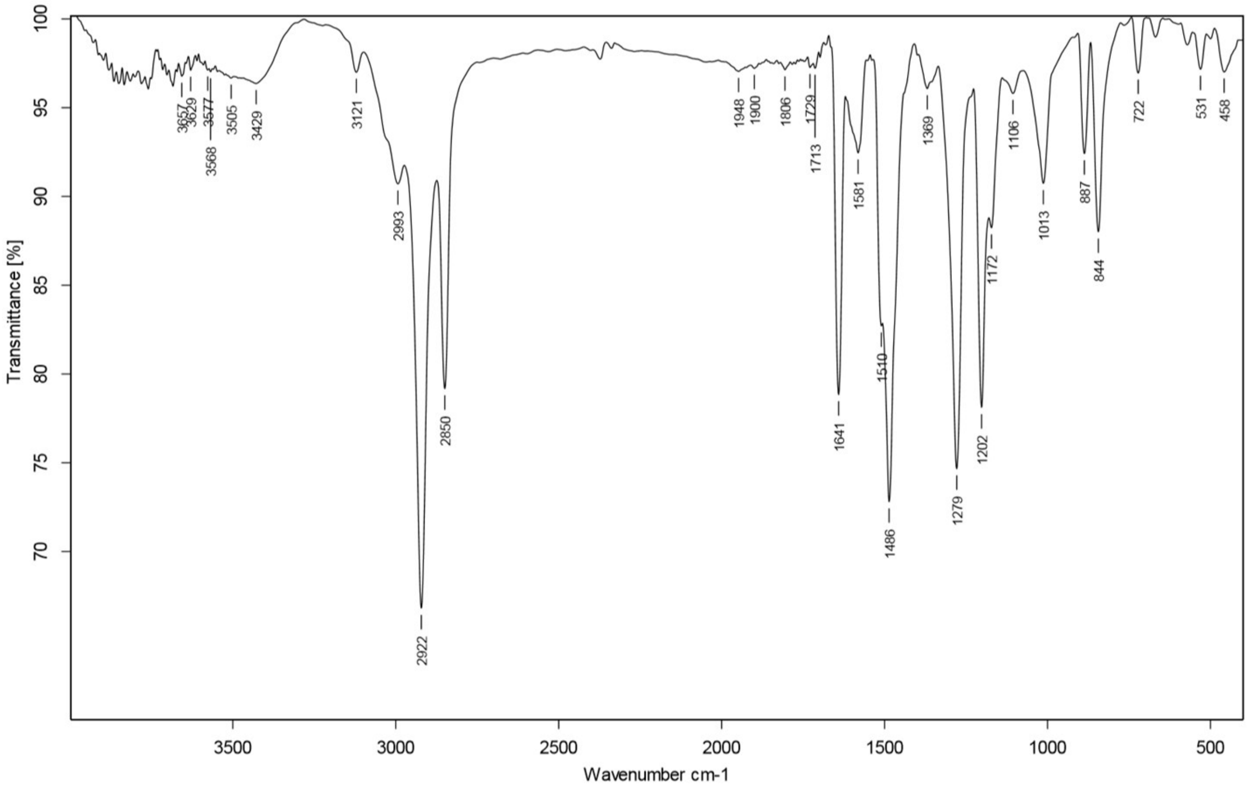

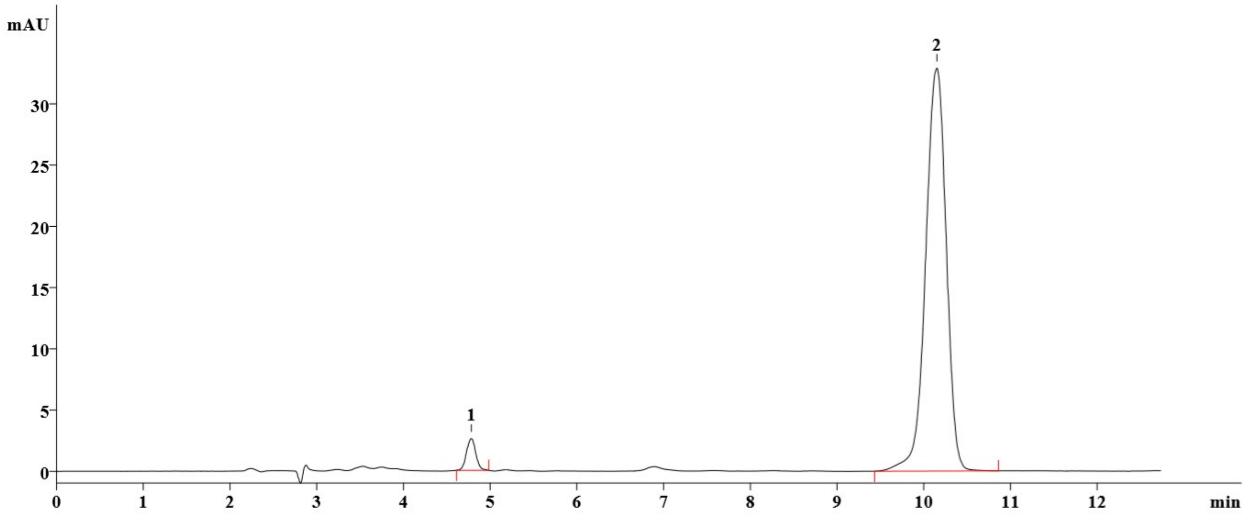

Peak table

Peak	Retention min	Area	Height	Width h/2	Area%	Type
1	4,78	19,308	2,581	0,117	3,429	BB :
2	10,15	543,829	32,857	0,254	96,571	BB :

23, 4OB4POB-9, Br

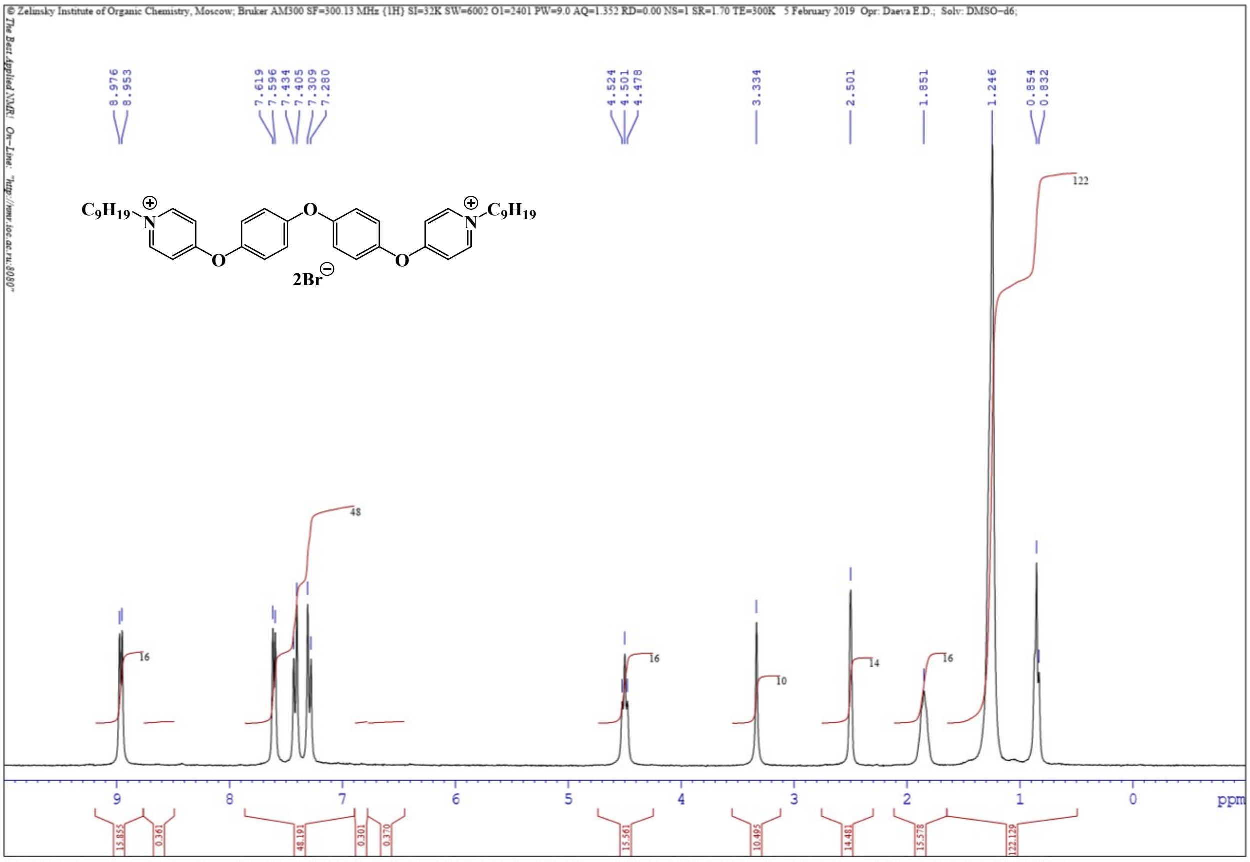

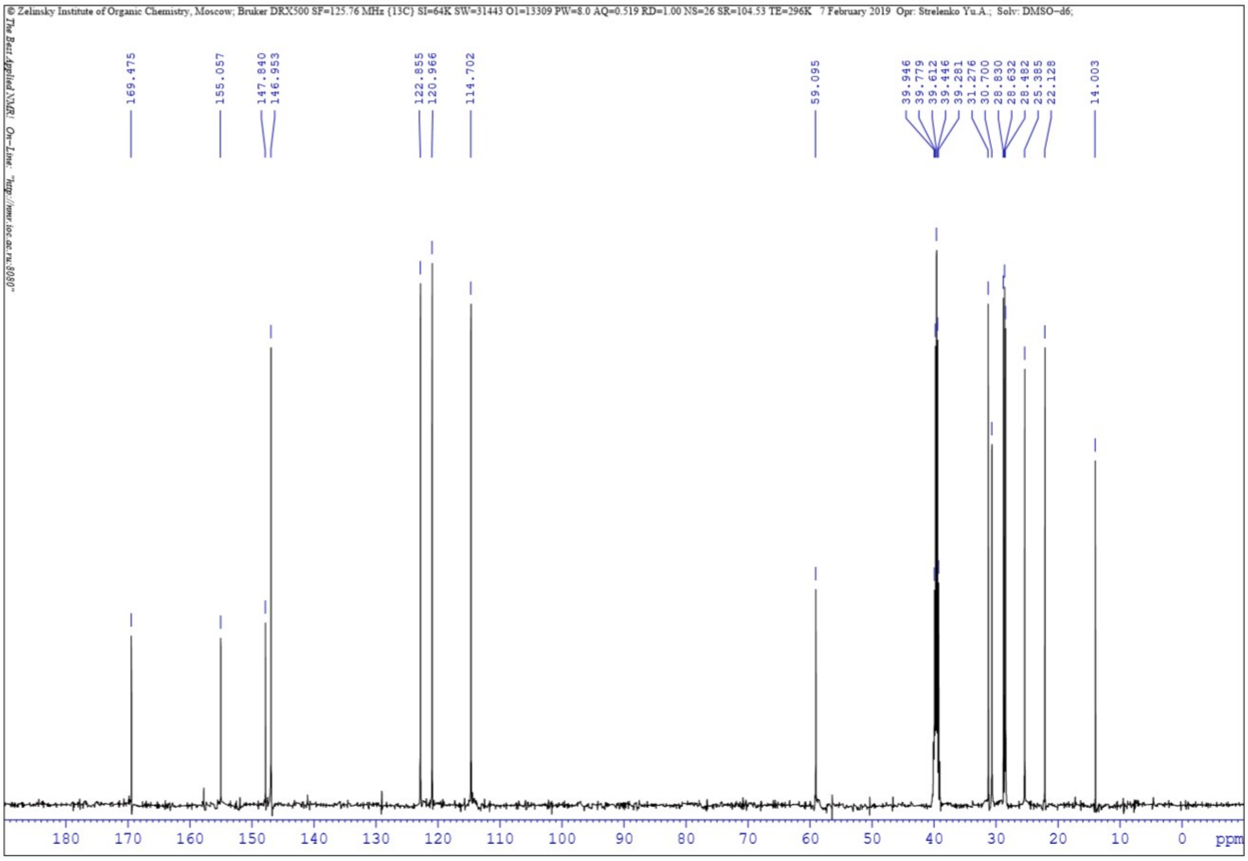

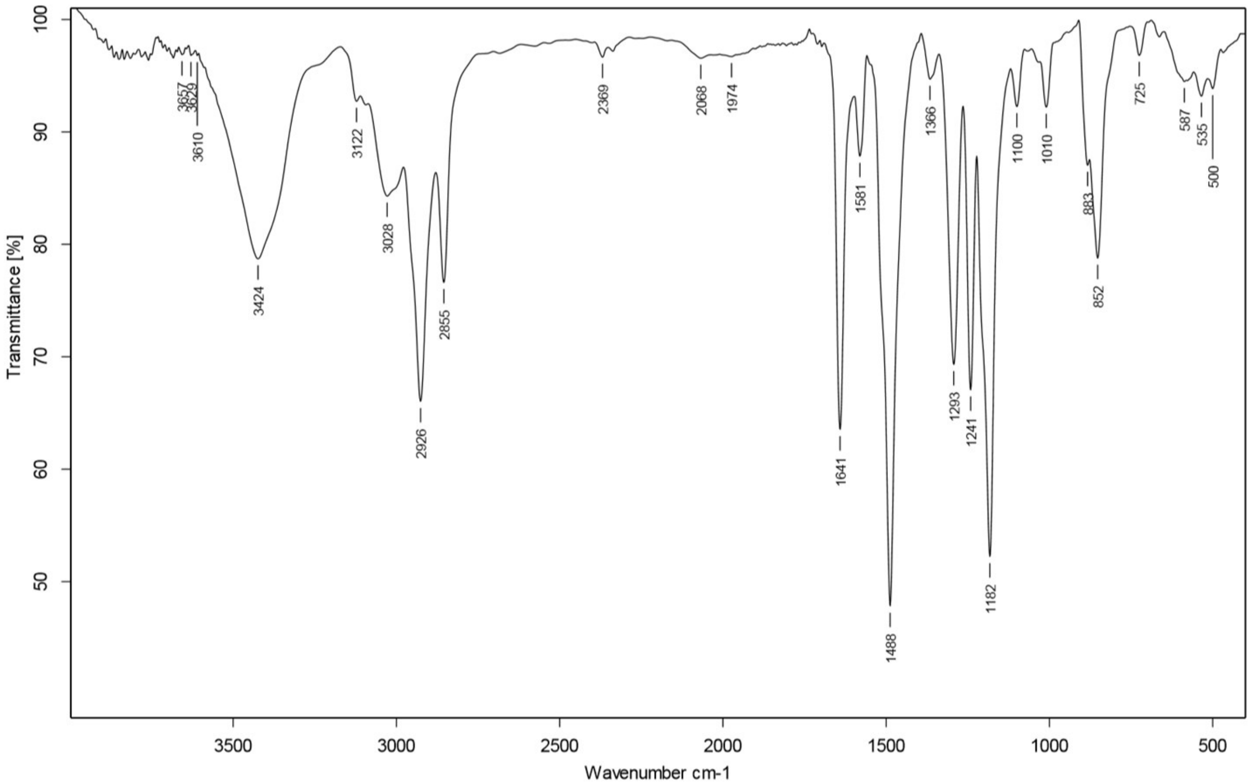

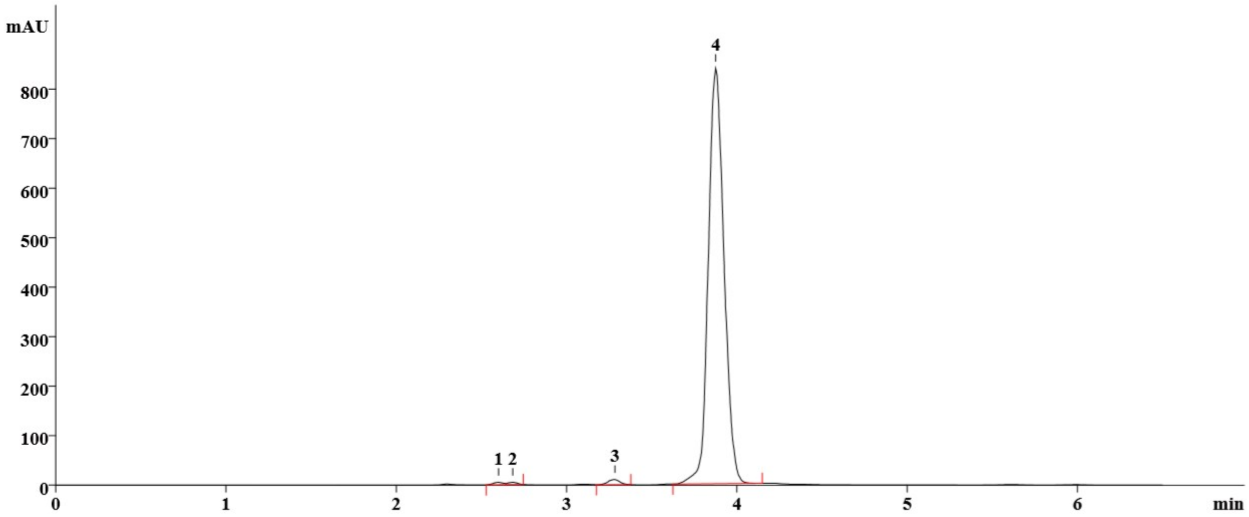

Peak table

Peak	Retention min	Area	Height	Width h/2	Area%	Type
1	2,60	19,510	4,795	0,0768	0,357	BD :
2	2,68	17,560	4,507	0,0733	0,321	DB :
3	3,28	49,678	10,370	0,0747	0,908	BB :
4	3,88	5383,436	836,810	0,0985	98,414	BB :

24, 4OB4POB-10, Br

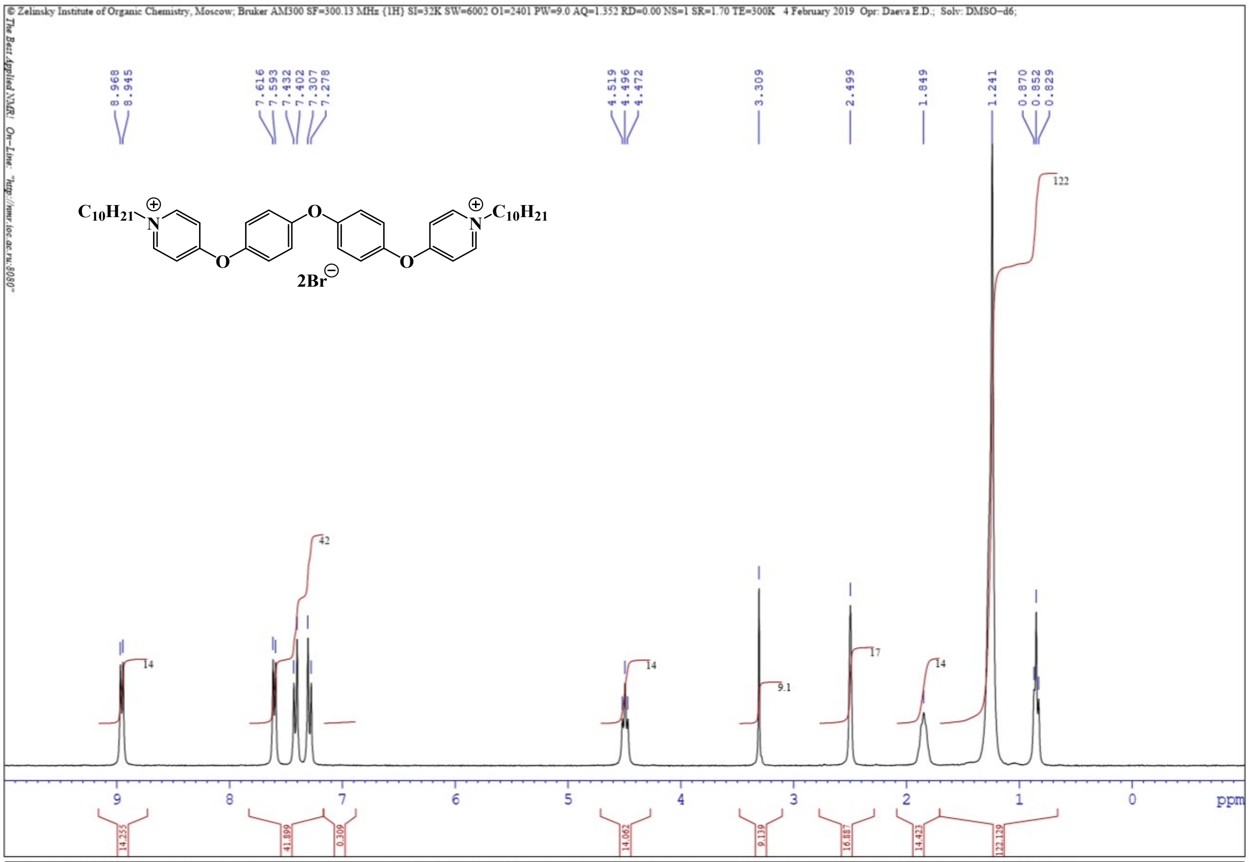

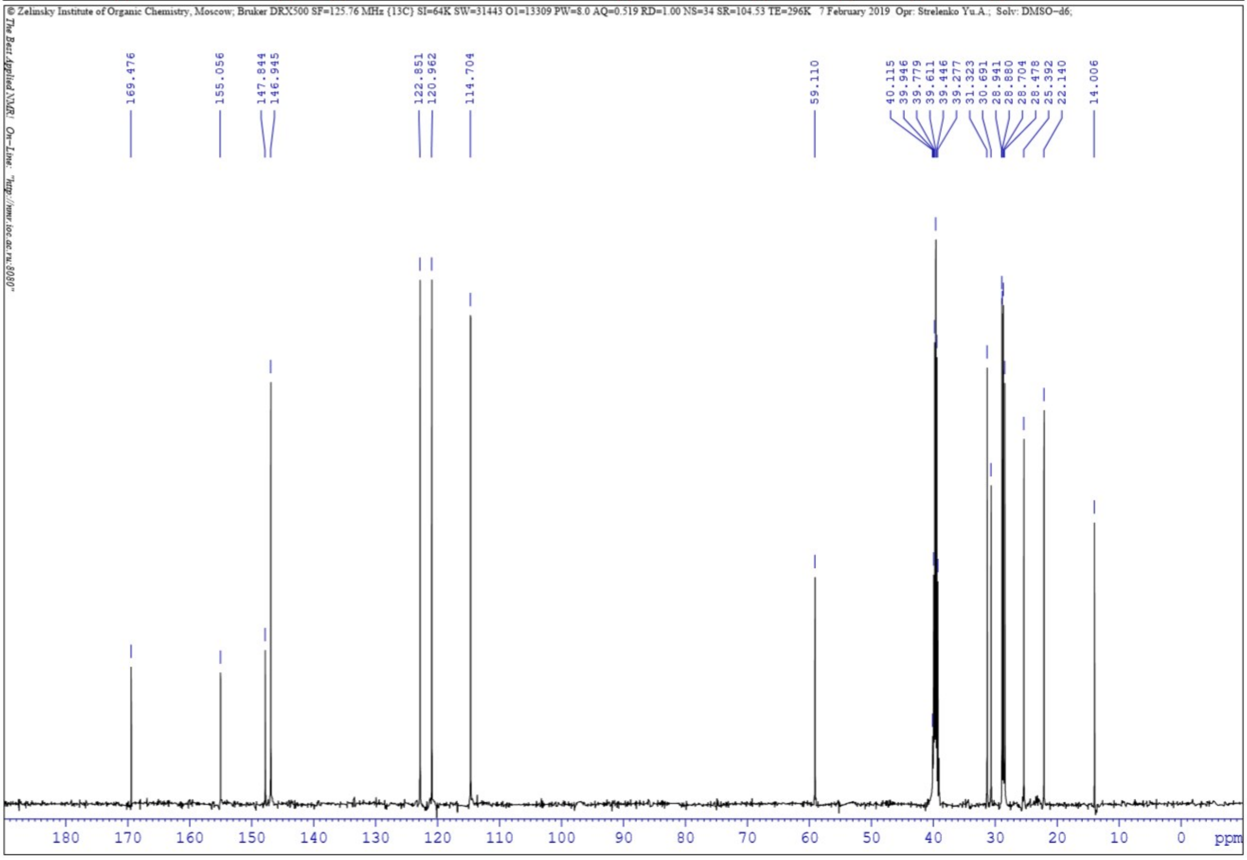

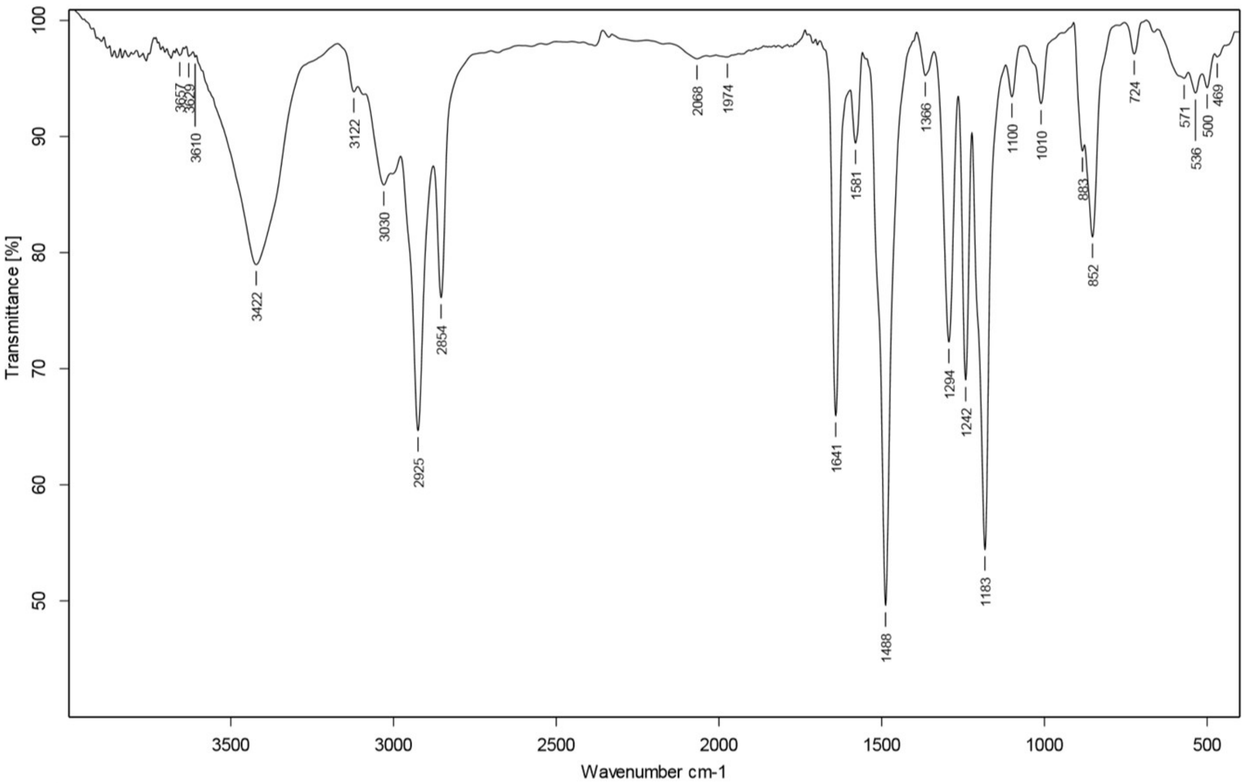

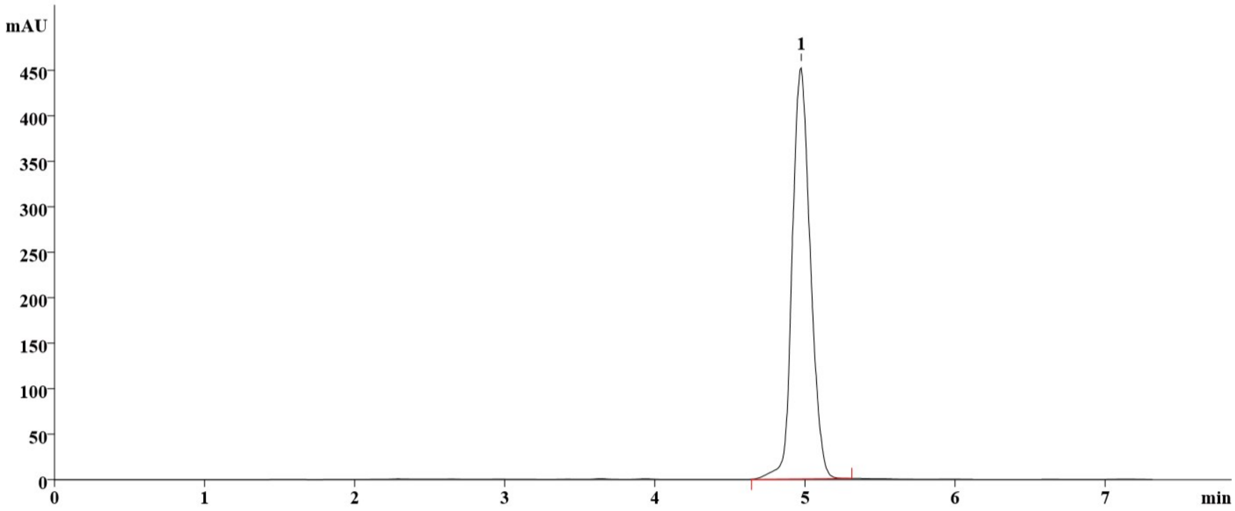

Peak table

Peak	Retention min	Area	Height	Width h/2	Area%	Type
1	4,97	3784,916	451,651	0,131	100,00	BB :

25, 4OB4POB-10, Cl

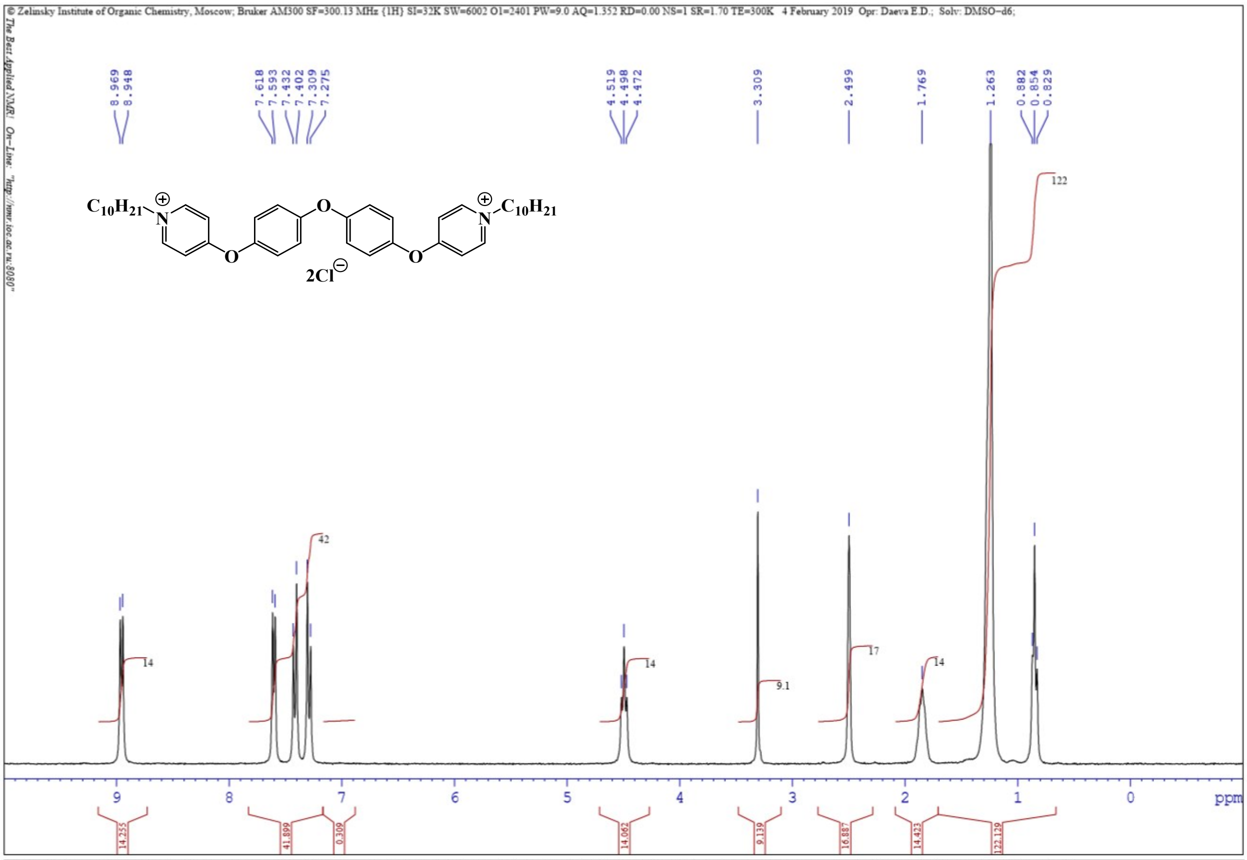

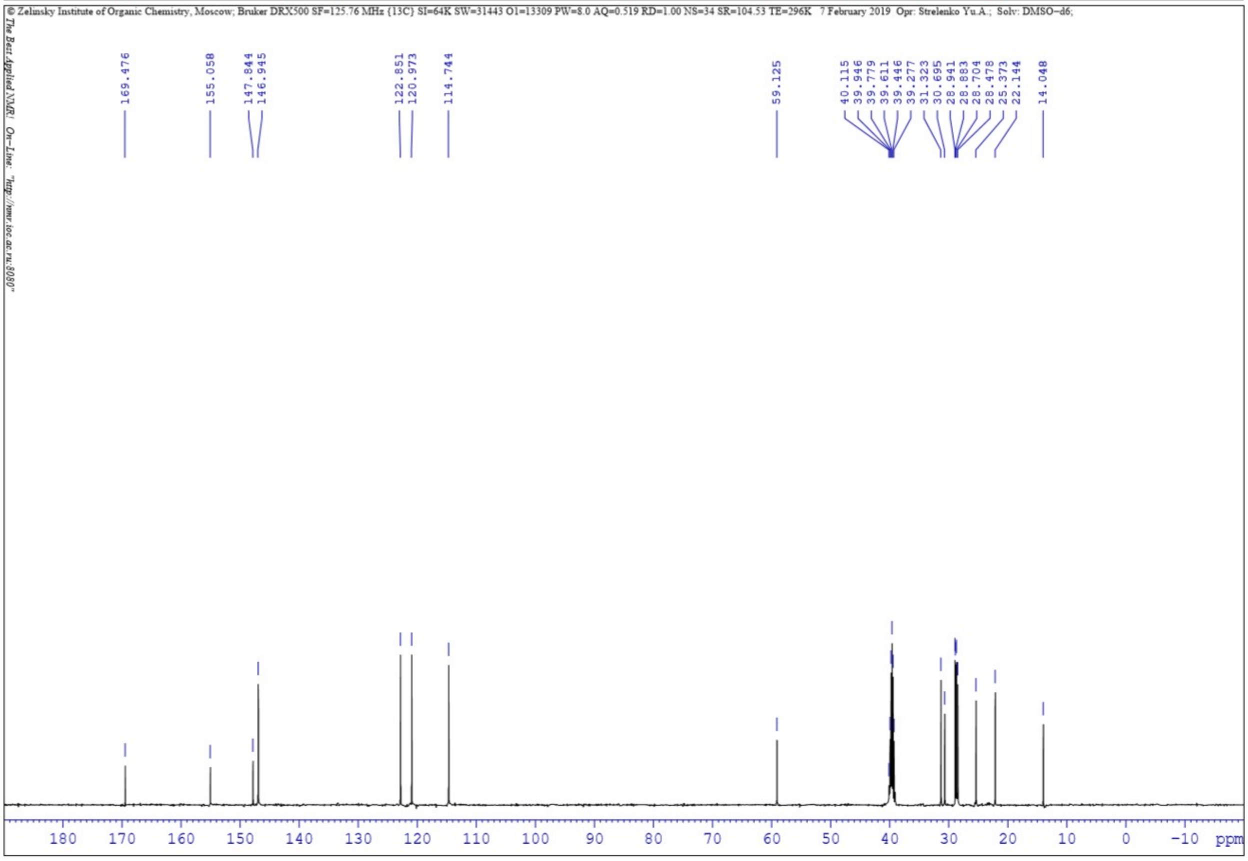

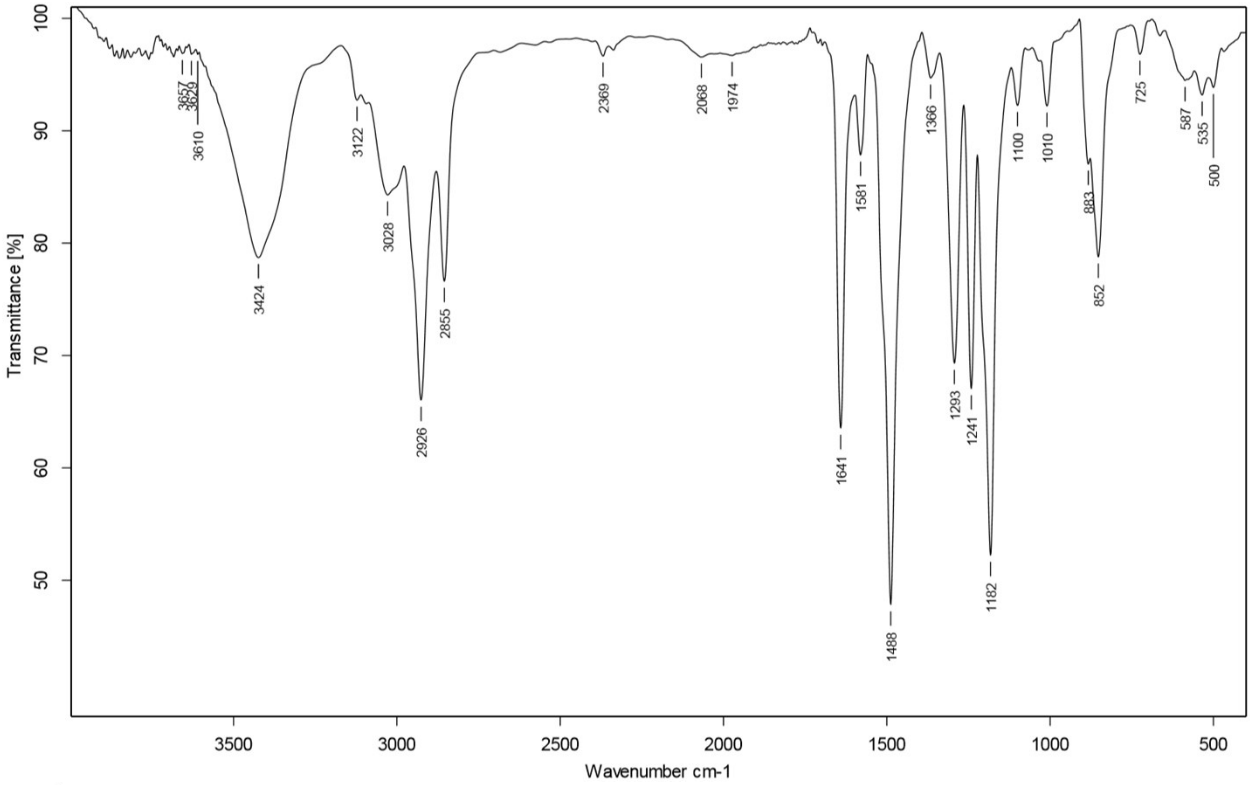

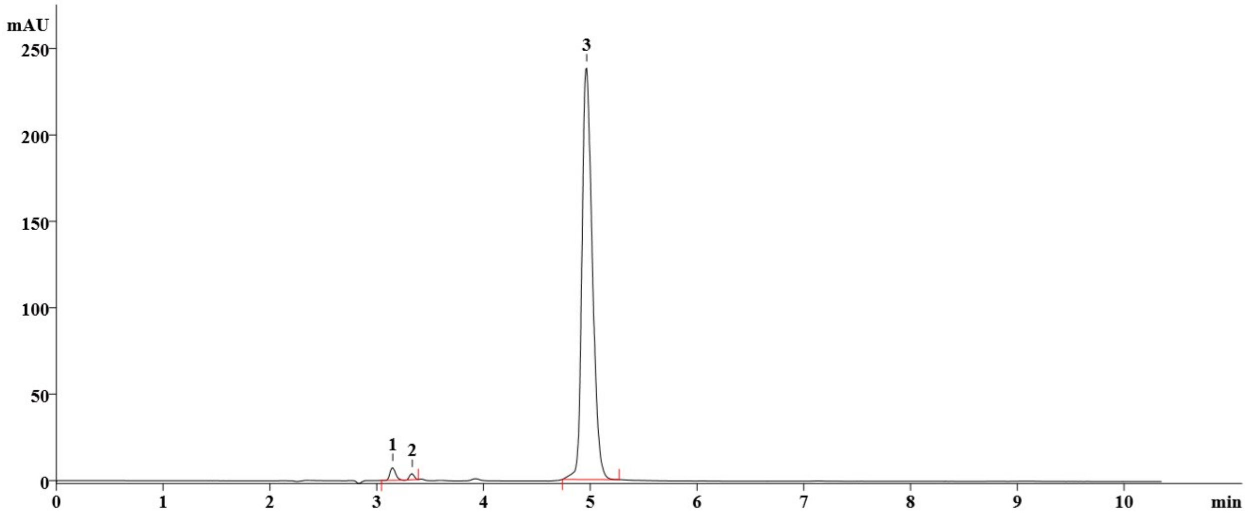

Peak table

Peak	Retention min	Area	Height	Width h/2	Area%	Type
1	3,15	27,011	7,225	0,0582	1,644	BD :
2	3,33	10,525	3,416	0,0516	0,640	DB :
3	4,96	1605,772	238,137	0,103	97,716	BB :

26, 4OB4POB-10, I

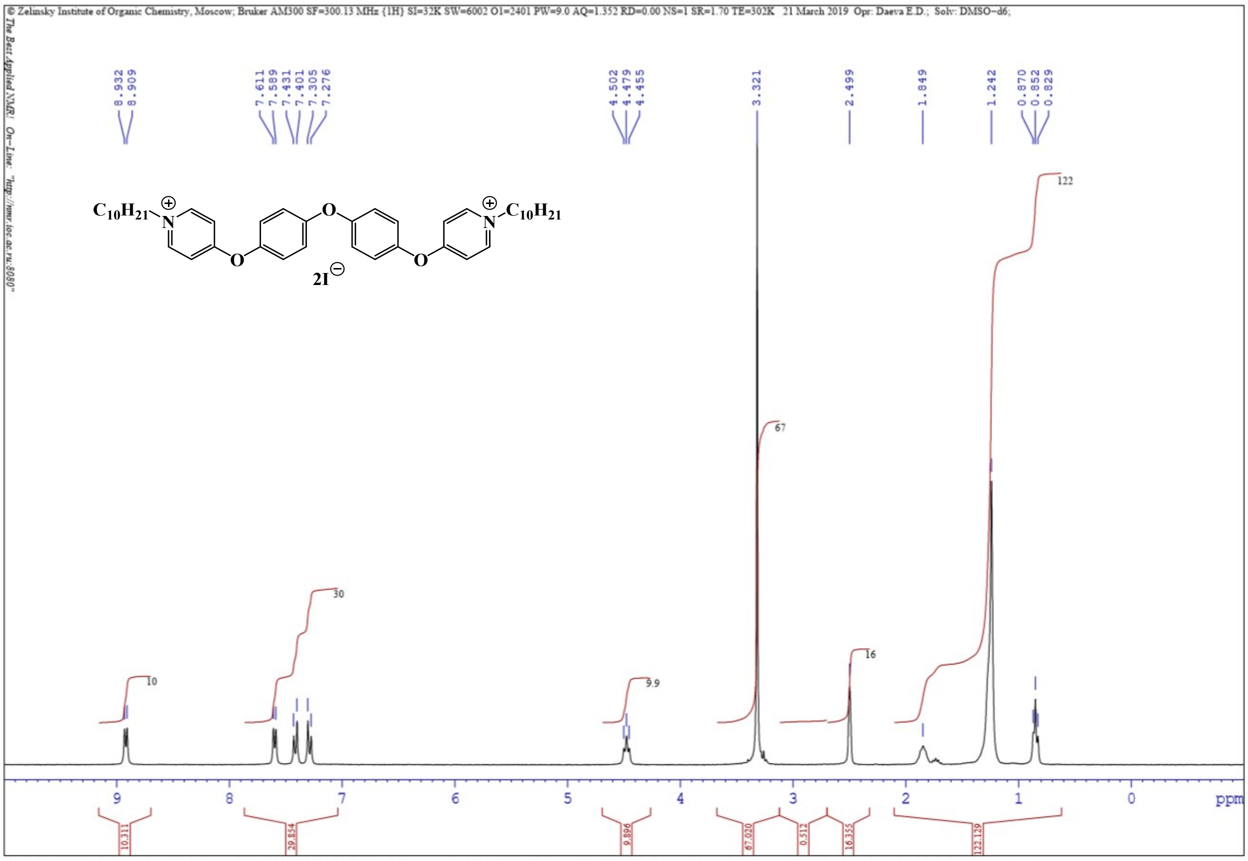

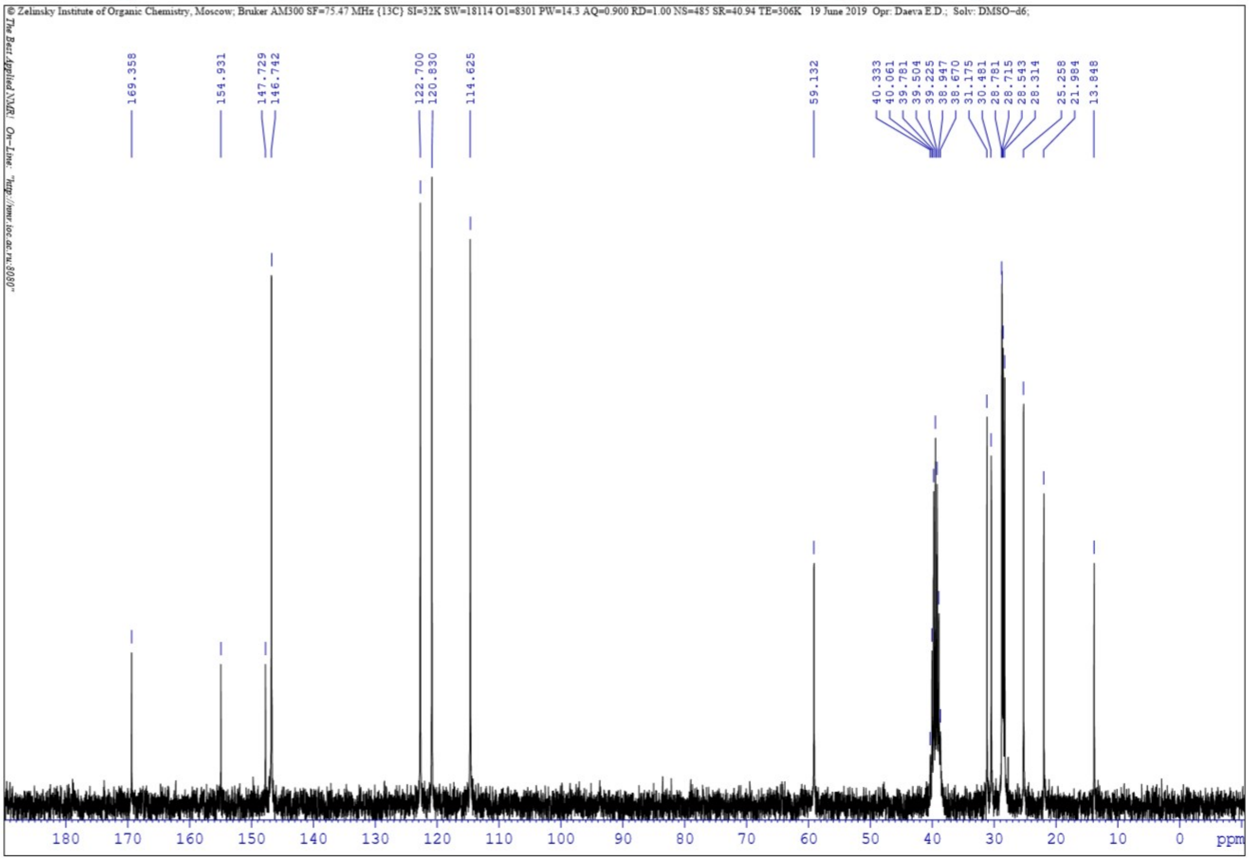

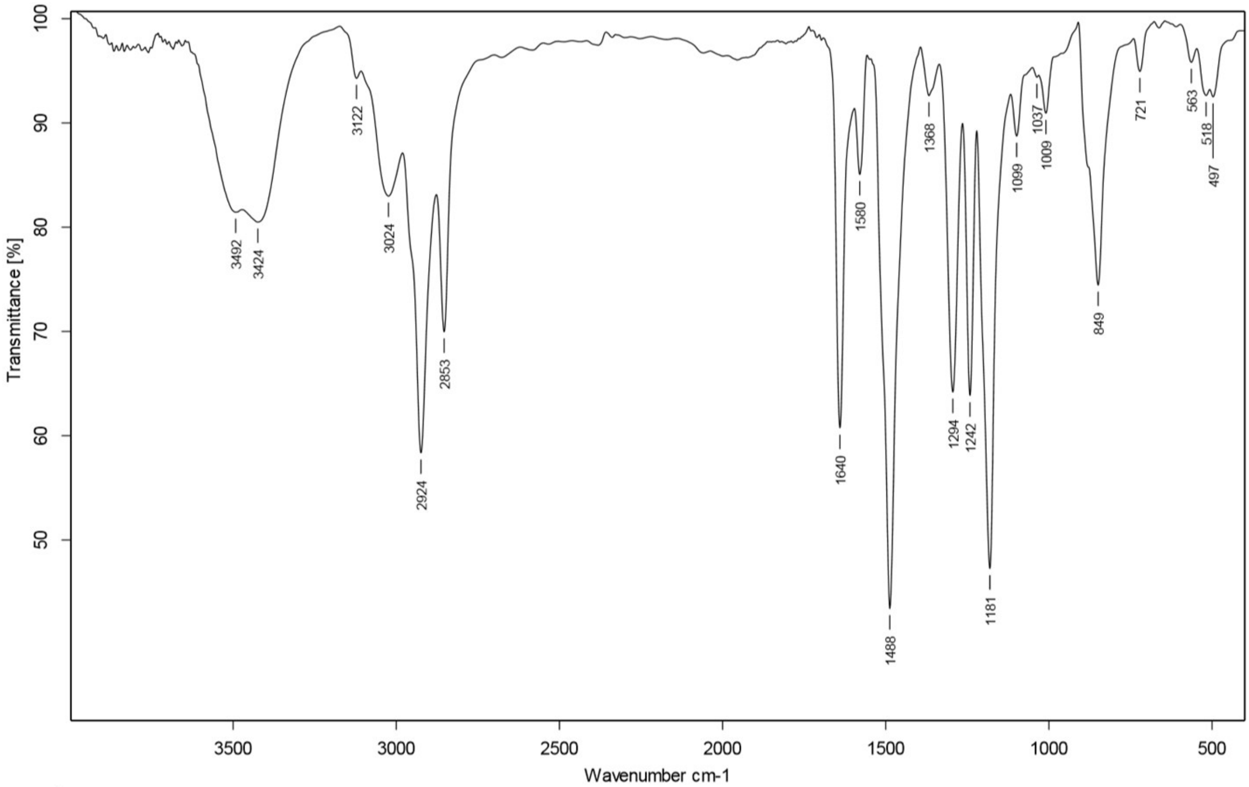

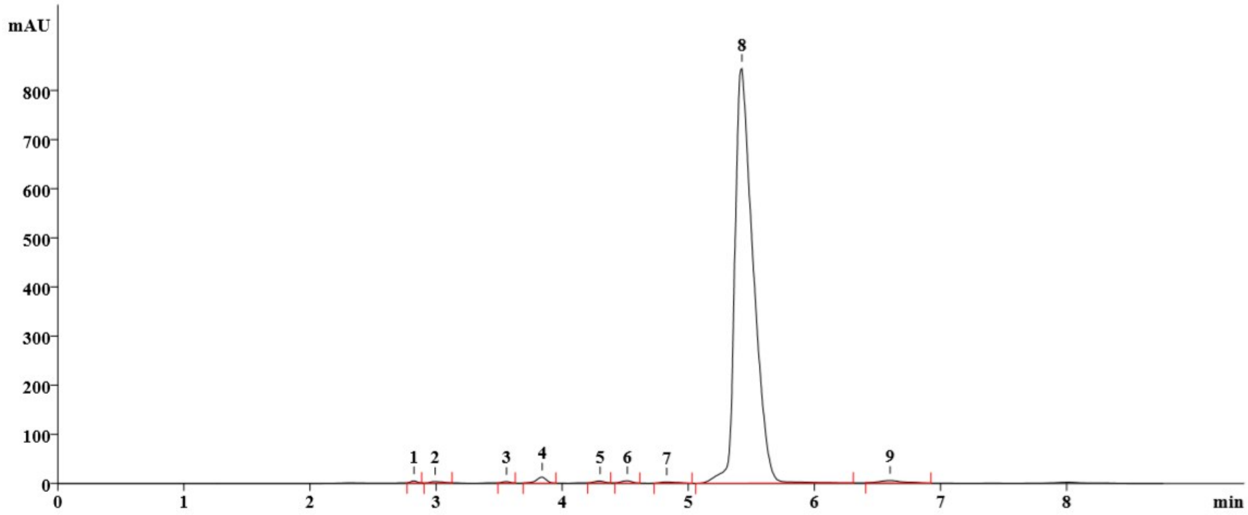

Peak table

Peak	Retention min	Area	Height	Width h/2	Area%	Type
1	2,82	13,446	3,930	0,0547	0,153	BB :
2	2,99	17,697	2,801	0,107	0,202	BB :
3	3,56	11,914	2,814	0,0692	0,136	BB :
4	3,84	66,563	12,070	0,0835	0,759	BB :
5	4,30	20,134	3,717	0,0876	0,230	BB :
6	4,51	25,942	4,562	0,0937	0,296	BB :
7	4,83	22,148	2,279	0,176	0,253	BB :
8	5,42	8528,019	843,565	0,156	97,265	BB :
9	6,60	61,979	4,895	0,171	0,707	BB :

27, 4OB4POB-11, Br

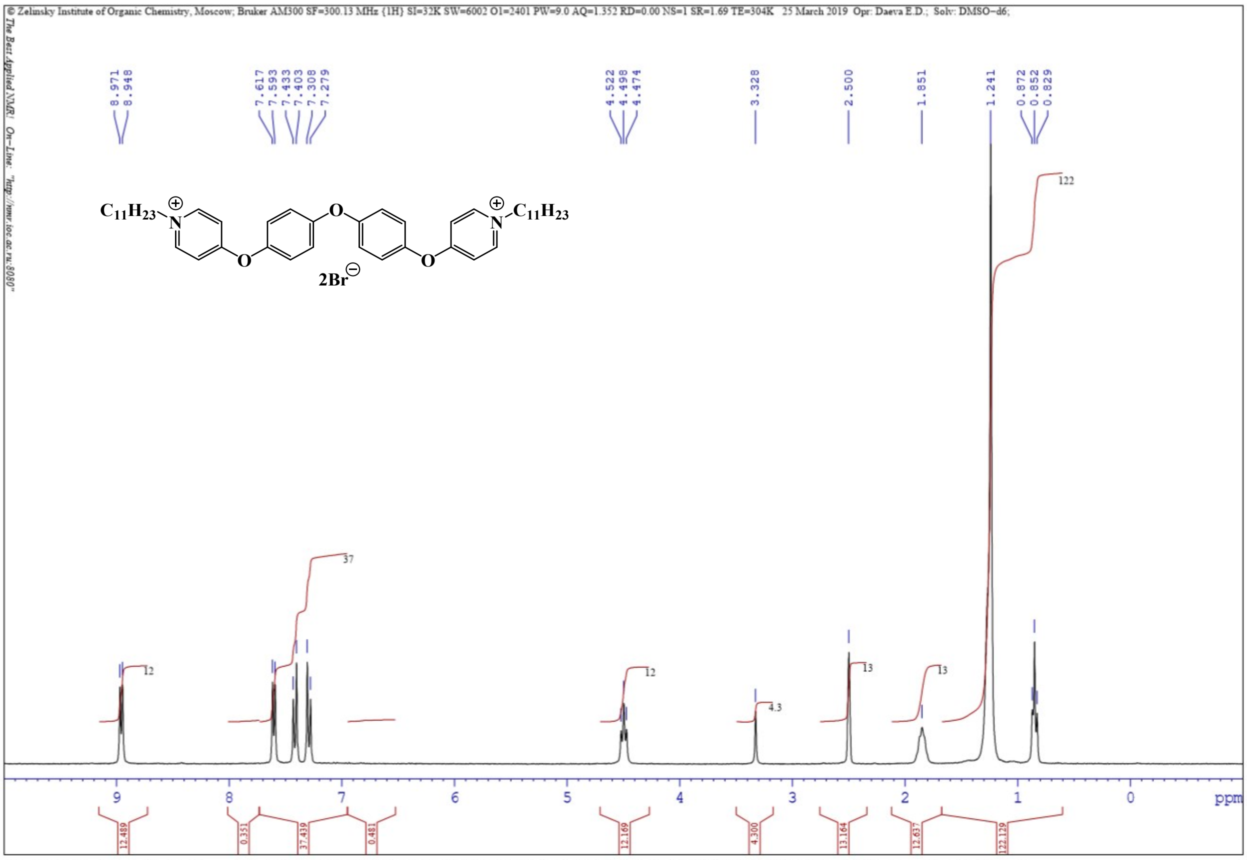

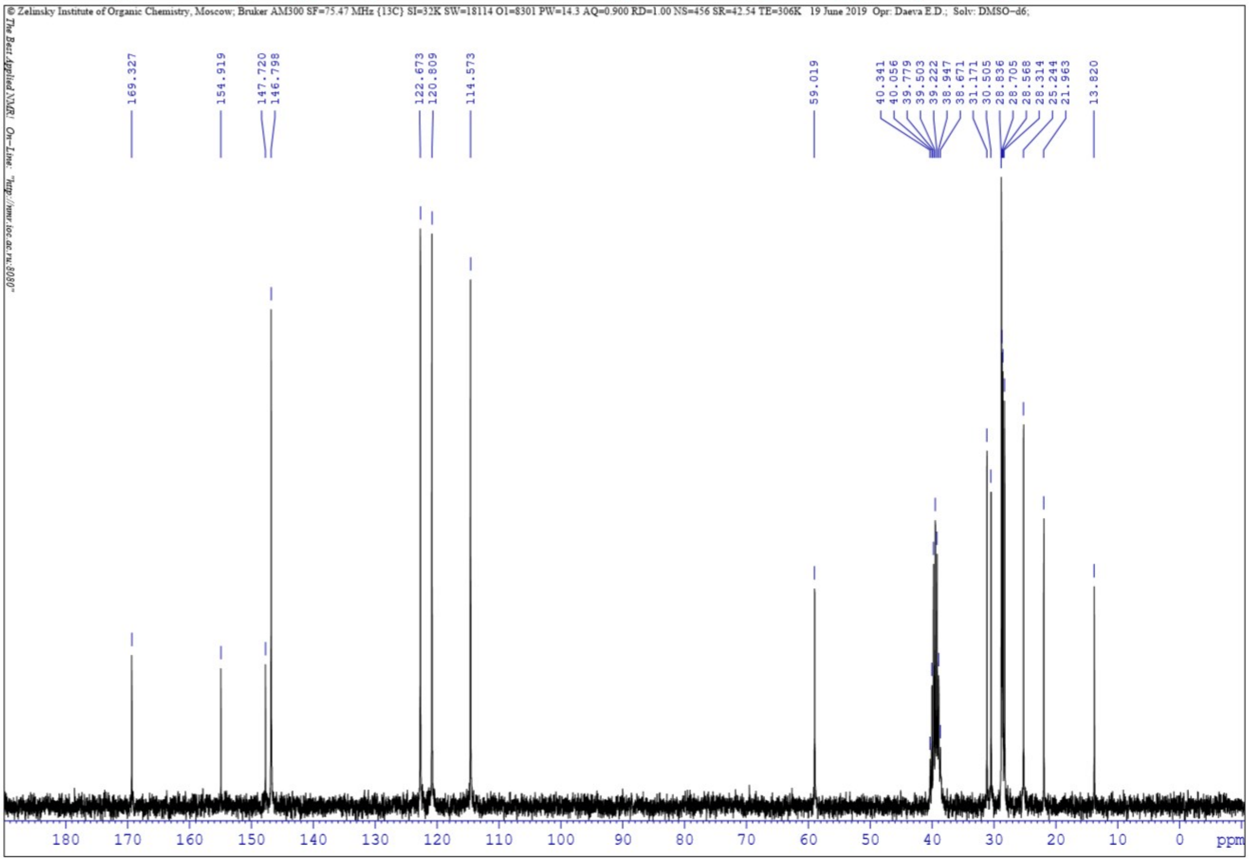

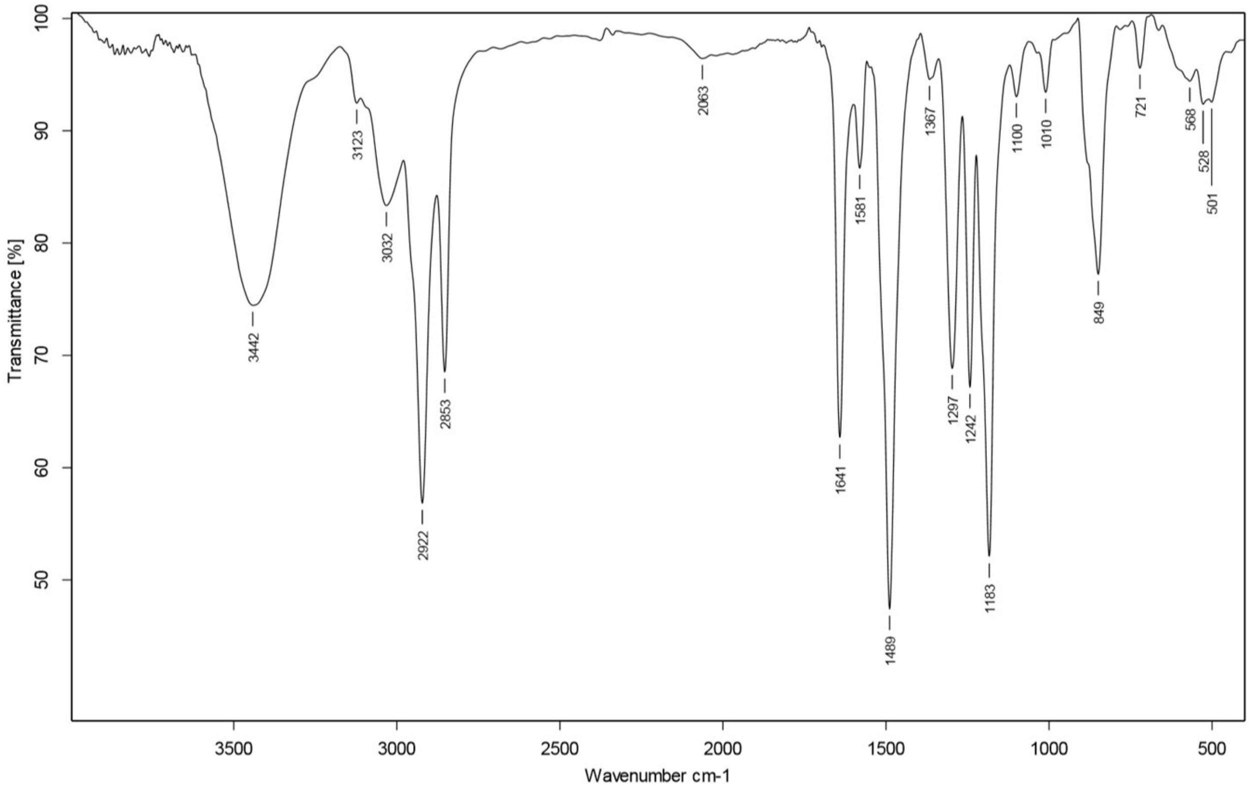

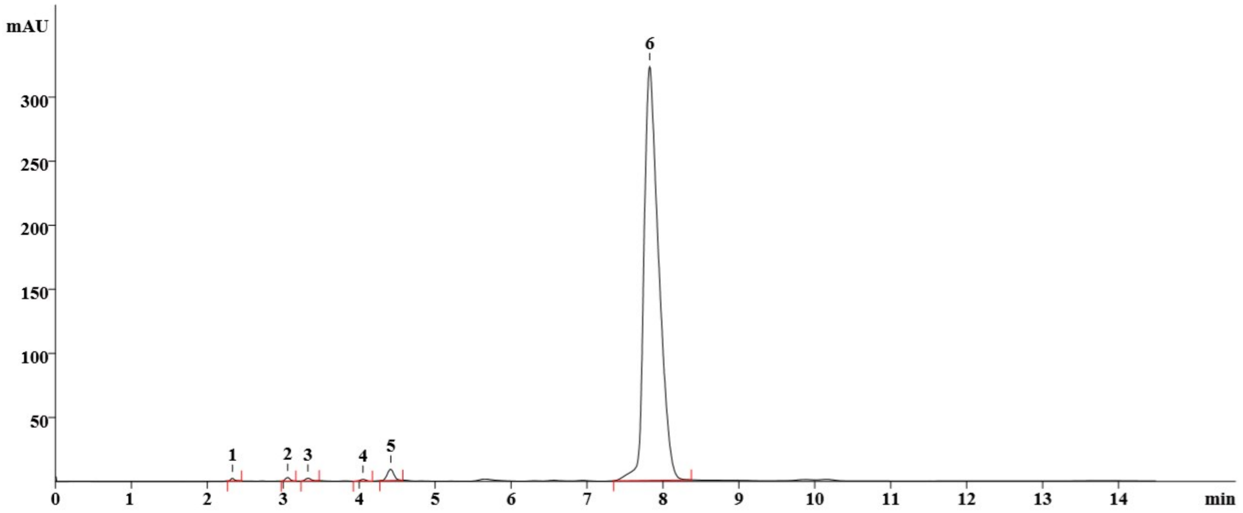

Peak table

Peak	Retention min	Area	Height	Width h/2	Area%	Type
1	2,33	7,286	1,957	0,0516	0,164	BB :
2	3,06	11,655	2,712	0,0677	0,263	BB :
3	3,33	10,483	2,049	0,0802	0,236	BB :
4	4,05	7,135	1,334	0,0845	0,161	BB :
5	4,41	53,017	8,696	0,0938	1,194	BB :
6	7,83	4349,826	322,766	0,209	97,982	BB :
